# Negative-Control Calibration and E-Value Bounding of Cancer-Survivor Mortality Contrasts: A Design-Aware Competing-Risks Analysis

**DOI:** 10.3390/cancers18213544

**Published:** 2026-11-04

**Authors:** Irina Naskinova, Mikhail Kolev, Meglena Lazarova, Hristo Kalinov, Velin Klisurov

**Affiliations:** 1 Department of Mathematics, University of Architecture, Civil Engineering and Geodesy (UACEG), 1 Hristo Smirnenski Blvd., 1164 Sofia, Bulgaria; inaskinova_fte@uacg.bg (I.N.); mikhail.kolev@uwm.edu.pl (M.K.); 2 Department of Applied Computer Science and Mathematical Modelling, Faculty of Mathematics and Computer Science, University of Warmia and Mazury in Olsztyn, Słoneczna 54, 10-710 Olsztyn, Poland; 3 Department of Mathematical Modelling and Numerical Methods, Faculty of Applied Mathematics and Informatics, Technical University of Sofia, 8 “Kliment Ohridski” Blvd., 1000 Sofia, Bulgaria; 4 Faculty of Mathematics and Informatics, Sofia University “St. Kliment Ohridski”, 5 James Bourchier Blvd., 1164 Sofia, Bulgaria; hkalinov@uni-sofia.bg (H.K.); fn62211@g.fmi.uni-sofia.bg (V.K.)

**Keywords:** cancer survivorship, competing risks, Fine–Gray subdistribution hazard, negative control, negative-control exposure, E-value, left truncation, net survival, unmeasured confounding, cardio-oncology

## Abstract

Objective: Cardiovascular death among cancer survivors is widely studied in population surveys, yet the survivor-versus-non-survivor contrast those studies rest on is rarely tested for confounding, and a survey cohort of prevalent survivors is left-truncated by design. The objective of this article is to test that contrast itself. Methods: The methods we used built a nationally representative survivor cohort from the National Health and Nutrition Examination Survey (NHANES) 1999–2018 (3826 survivors among 52,398 examined adults) linked to the National Death Index, with 288 deaths from diseases of the heart and 497 cancer deaths, and paired survey-weighted Fine–Gray competing-risks models with designed probes of what these data cannot support. Results: Our results are that accidental death, a negative-control outcome, was raised among survivors (hazard ratio 1.78, 95% CI: 0.89–3.54) and equally among participants whose only malignancy was a skin cancer (2.01, 0.87–4.66). For cardiovascular death, that negative-control exposure reproduced the survivor contrast (0.87, 0.65–1.18 against 0.84, 0.71–1.00), leaving a calibrated contrast of 0.96 (0.70–1.32). Refitting on the time-since-diagnosis scale with delayed entry left the comorbidity index’s association with cardiovascular death unchanged (1.41, 1.22–1.64 against 1.40, 1.24–1.59). Net survival against internally modeled expected mortality, 0.897 (0.869–0.924) at 10 years, agreed with the recorded cancer-death incidence of 0.115 (0.102–0.127). Conclusions: The conclusions drawn are that what such a survey establishes about survivor mortality is narrower than it appears.

## Simple Summary

Many people who have had cancer die of heart disease rather than of cancer, and large health surveys are often used to study that pattern. This study asks how much such surveys can establish. We built a group of 3826 adults who reported a cancer diagnosis in a national United States survey, linked them to death records, and modeled death from heart disease and death from cancer together. We then tested the comparison itself. People with a cancer history differed from people without one even in accidental death, which no cancer mechanism explains, and the small group whose only cancer was a skin cancer showed a similar difference. Survival measured against the death rates expected for people of the same age and background came close to the recorded cancer deaths. What such a survey can establish about heart-disease death after cancer is narrower than it appears.

## 1. Introduction

Cardiovascular disease has emerged as a leading non-cancer cause of death among people who survive a cancer diagnosis, a consequence of both shared risk factors (age, obesity, inflammation, and smoking) and the cardiotoxic potential of anthracycline chemotherapy, HER2-targeted agents such as trastuzumab, chest or mediastinal radiotherapy, and androgen-deprivation therapy [1,2]. In United States registry data, cardiovascular mortality is raised across most cancer sites relative to the general population, and heart disease accounts for a large share of the non-cancer deaths that follow a cancer diagnosis [3,4]. The European Society of Cardiology’s 2022 cardio-oncology guideline and the American Society of Clinical Oncology’s guideline on cardiac dysfunction in adult cancer survivors both recommend baseline cardiovascular risk stratification before cardiotoxic treatment and structured surveillance afterward [1,5]. As of 2022, an estimated 18.1 million people were living with a history of cancer in the United States, a number projected to exceed 26 million by 2040 [6,7], so the population at risk of this competing cause of death is large and growing.

Most of the evidence base comes from cancer registries or from clinical trial and single-institution cohorts. A retrospective English study of 104,028 adults diagnosed with one of nine common cancers found that cardiovascular death overtook the primary cancer as the leading cause of death between two and eleven years after diagnosis among survivors aged 80 years or older at diagnosis [2]. Registry sources of this kind offer large samples and clinically confirmed diagnoses, often with treatment detail, but they are not population-representative: they under-sample patients who are never referred to a cancer center, over-represent patients engaged with the health system, and, critically for the question of *who* dies of cardiovascular disease rather than cancer, almost never carry the detailed, harmonized cardiometabolic risk-factor panel (blood pressure, lipids, glycemic markers, inflammatory markers, and comorbidity burden) needed to model the competing hazard jointly with cancer-specific covariates. The National Health and Nutrition Examination Survey (NHANES), linked to the National Death Index through its public-use Linked Mortality File (LMF), offers the opposite trade-off: a population-representative, complex-survey sample with a rich examination-based covariate panel, self-reported cancer history, and cause-specific mortality follow-up, at the cost of no registry confirmation of diagnosis and, the central limitation of this study, no record of cancer treatment at all.

A smaller literature uses NHANES itself to study mortality among self-reported cancer survivors. Zhao et al. [8] followed 4366 NHANES 1999–2018 cancer survivors and related a nutrition-based prognostic index to all-cause, cancer-specific, and non-cancer mortality. Moon and Mun [9] document the construction problem this literature shares: NHANES has no single validated cancer-history variable and no laboratory confirmation, so every NHANES-derived survivor cohort, including the present one, rests on self-reporting. Three gaps remain in that literature. No NHANES-based analysis we are aware of models cardiovascular and cancer death among survivors as formally competing risks with a survey-design-aware subdistribution-hazard model, so one cause’s covariate effects cannot be read relative to the other [8,10,11]. Treatment exposure is unobserved in this and in most population surveys, and we found no such study that pairs a labeled indirect probe of treatment-related cardiotoxicity with a quantitative bound on the unmeasured confounding it cannot rule out. We also found none that tests whether the survivor-versus-non-survivor contrast is itself contaminated by general frailty or differential healthcare surveillance, which a negative-control outcome can expose [12,13].

This study pairs survey-weighted Fine–Gray competing-risks models for cardiovascular and cancer death in a nationally representative survivor cohort with four designed probes of what these data cannot support: a negative-control outcome with no plausible cancer mechanism, a negative-control exposure that shares the survivor cohort’s reporting and surveillance pathway without its cancer mortality, an indirect site-level cardiotoxicity contrast carried with an E-value bound, and a comparison of the full covariate panel against age and sex alone. Delayed entry on the time-since-diagnosis scale and an excess-mortality analysis that uses no cause-of-death information address the two ways in which a survey cohort of prevalent survivors can mislead: left truncation and cause-of-death misattribution.

## 2. Materials and Methods

### 2.1. Research Question and Hypotheses

The primary question of this study is which baseline demographic, comorbidity, and laboratory characteristics are associated with the relative subdistribution hazard of cardiovascular versus cancer death among cancer survivors in a nationally representative sample, under a design-aware competing-risks framework. The secondary question is how much of any survivor-related mortality contrast in these data can plausibly reflect unmeasured confounding rather than a cancer- or treatment-specific pathway: confounding by general frailty and surveillance intensity, tested with a negative-control outcome and a negative-control exposure, and confounding by unobserved cancer treatment, tested with an E-value on an indirect site-level cardiotoxicity proxy.

Three hypotheses were fixed before the models were fit. Higher comorbidity burden and older age would be associated with a higher relative hazard of cardiovascular death. A higher number of reported cancer sites would be associated with a higher relative hazard of cancer death. The negative-control probes would reveal non-zero confounding, which would motivate explicit quantification rather than a claim of a clean contrast.

### 2.2. Design of the Analysis

The analysis has five components. The first is a pair of survey-weighted Fine–Gray subdistribution-hazard models, for cardiovascular and for cancer death, with a fully adjusted 25-covariate specification as the primary model and a parsimonious ten-covariate specification as a check, each with design-aware confidence intervals from a delete-one-primary-sampling-unit jackknife applied to the whole transform-plus-regression pipeline. The second is a negative-control outcome, accidental (external-cause) death, which has no plausible mechanistic link to cancer or its treatment, together with a negative-control exposure, the survivors whose only reported malignancy was non-melanoma or unknown-type skin cancer, who share the reporting and surveillance pathway of the analytic cohort with little cancer mortality and no cardiotoxic treatment. The third is an indirect, explicitly labeled site-level cardiotoxicity contrast carrying a VanderWeele–Ding E-value that bounds the unmeasured confounding needed to explain it away. The fourth addresses the two ways a survey cohort of prevalent survivors can mislead: delayed entry on the time-since-diagnosis scale, for left truncation, and an excess-mortality analysis against internally modeled expected mortality, which uses no cause-of-death information. The fifth is a cause-specific gradient-boosted ensemble with SHapley Additive exPlanations (SHAP) attribution, read against what age and sex alone achieve.

The cohort itself is a fully inventoried NHANES 1999–2018 cancer-survivor sample of 3826 adults with harmonized self-reported diagnosis, site, and time-since-diagnosis variables, linked to cause-specific mortality through the 2019 LMF release. Sensitivity analyses address the cardiovascular-outcome definition, listwise deletion, which leaves 2225 of the 3826 survivors covariate-complete, multicollinearity, the ridge penalty, and the exclusion of *C*-reactive protein.

### 2.3. Data Sources and Survivor Cohort Definition

The primary data source was ten pooled cycles of the National Health and Nutrition Examination Survey (1999–2000 through 2017–2018) [14], a complex, multistage probability survey of the civilian, non-institutionalized US population conducted by the National Center for Health Statistics (NCHS), combining a household interview with a mobile examination center (MEC) visit. Mortality follow-up came from the NHANES public-use Linked Mortality File (2019 release) [15], which links NHANES participants to National Death Index records through 31 December 2019 and provides vital status, follow-up time, and an NCHS ten-category leading-cause-of-death recode.

Table S1 gives the full, sequential subsample inventory. Starting from the 101,316 participants across all ten cycles, we retained adults aged 20 years or older (55,081), then the subset with a valid MEC examination weight (52,398; required for the laboratory and anthropometric covariate panel). Among these, 4864 reported ever having been told they had cancer or a malignancy (NHANES item MCQ220), of whom 4830 had a recoverable cancer site from the harmonized MCQ230/MCQ240 (or MCD240) items. Survivors whose only reported cancer was non-melanoma or unknown-type skin cancer (*n* = 1038) were excluded, following the standard cancer-survivorship and SEER-incidence convention that treats this diagnosis as a distinct, much less lethal entity; these participants return in Section 2.6 as a negative-control exposure. No participant had an implausible age at diagnosis (age at diagnosis exceeding age at the MEC visit), so no correction was needed. The resulting analytic survivor cohort comprised 3826 adults. A computable time since diagnosis (age at MEC visit minus a valid age at diagnosis) was available for 3773 of them, with site and/or time-since-diagnosis missing for a documented subset carried forward as missing data rather than as an additional exclusion.

**Figure 1:**
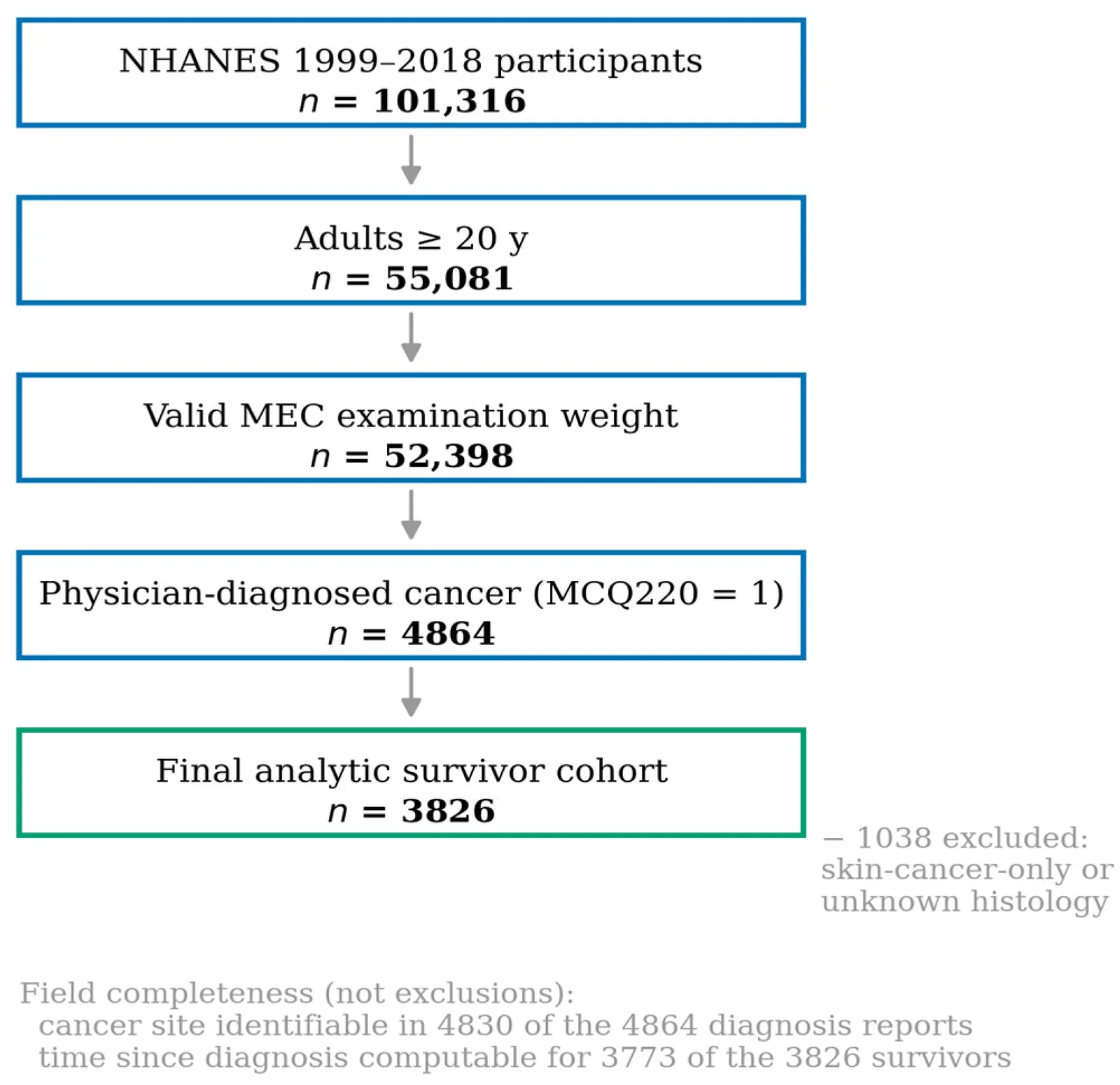
Derivation of the analytic cancer-survivor cohort from pooled NHANES 1999–2018. Each box gives the sample remaining after the stated criterion. Survivors whose only reported malignancy was non-melanoma or unspecified skin cancer were excluded, following the registry convention that treats those as a distinct and much less lethal entity. Arrows run downward from each criterion to the sample that satisfies it, in the order the criteria were applied. The final analytic cohort is outlined in green and the preceding steps in blue. The grey text beside the final box gives the exclusion applied at that step, and the grey text below it reports how complete two fields are within the cohort rather than any further exclusion.

Figure 1 shows the same cascade graphically. The identifiable-cancer-site count (4830) and the computable-time-since-diagnosis count (3773) appear there as annotations rather than as steps, because they describe how complete a field is within the cohort rather than how many people were removed from it.

### 2.4. Outcome Ascertainment

Vital status and cause of death were taken from the LMF’s NCHS ten-category leading-cause-of-death recode (UCOD_LEADING). Two properties of the public-use release govern how that recode can be used. First, to protect participant identity, NCHS substitutes synthetic values for the underlying cause of death or for the follow-up time in a small number of records, while vital status itself is never altered [16]. Second, the ten categories are not populated identically across cycles: in the 2015–2016 and 2017–2018 releases only diseases of the heart (001), malignant neoplasms (002), and the residual “all other causes” category (010) carry any deaths, and the seven intermediate categories, among them cerebrovascular disease (005) and accidents (004), are empty (Table S2).

A single mutually exclusive event indicator was constructed with four levels: 0 (alive or administratively censored at the 2019 follow-up cutoff), 1 (cardiovascular death: UCOD_LEADING code 001, diseases of the heart), 2 (cancer death: code 002, malignant neoplasms), and 3 (other-cause death: any other observed death). Defining cardiovascular death as diseases of the heart alone keeps the outcome coded the same way in all ten cycles. A wider definition that also counts cerebrovascular death is reported as a sensitivity analysis restricted to the eight cycles in which code 005 is populated, because in the last two cycles stroke deaths fall into the residual category and a pooled wider definition would therefore count them as competing events in those cycles only (Table S3). Analyses of accidental death, the negative-control outcome, are restricted to the same eight cycles for the same reason. This is the standard competing-risks coding: for a Fine–Gray model of a given cause, every other observed death code is treated as a competing event, never as ordinary censoring [10,11]. Follow-up time was months from the MEC examination to death or the 2019 administrative censoring date, converted to years.

### 2.5. Covariates, Including the Comorbidity Index

The covariate panel comprised age, sex, race/ethnicity, educational attainment, the family income-to-poverty ratio, body-mass index (BMI), systolic and diastolic blood pressure, total and high-density lipoprotein (HDL) cholesterol, glycated hemoglobin (HbA1c), *C*-reactive protein, the neutrophil-to-lymphocyte ratio, hemoglobin, serum albumin, self-reported hypertension and diabetes diagnoses, smoking status, alcohol use, physical activity, past-12-month healthcare visits, self-rated general health, and the two cancer-history-specific covariates, time since diagnosis and the number of distinct cancer sites reported. Questionnaire answers of “refused” or “don’t know” were set to missing before any recoding, with one deliberate exception: hypertension, diabetes, and the comorbidity index count a condition only when the participant answered “yes”, so that the index equals the sum of its components exactly (Section 2.15). Supplementary File S3 gives the source item for each covariate, the harmonization applied across cycles, and the counts affected by that recoding rule.

The comorbidity index sums seven self-reported condition flags: hypertension (BPQ020), diabetes (DIQ010), and five cardiovascular/cerebrovascular diagnoses (congestive heart failure, coronary heart disease, angina, myocardial infarction, and stroke; MCQ160B–F). Five of its seven components are themselves prior cardiovascular diagnoses, and hypertension and diabetes are also entered as separate covariates in every model, so the comorbidity index is substantially, and not incidentally, a count of prior cardiovascular morbidity, and this shapes how its association with cardiovascular death should be read (Section 4.2).

### 2.6. Cardiotoxicity Proxy, Negative-Control Outcome, and Negative-Control Exposure

NHANES records no cancer treatment: not chemotherapy regimens, not anthracycline or trastuzumab exposure, not chest or mediastinal radiotherapy fields, and not androgen-deprivation therapy. These are the principal documented mechanisms of cardiovascular harm in cardio-oncology [1]. This is the largest unmeasured confounder in this study and cannot be corrected with these data. Two design features address it as directly as the data allow, rather than leaving it as an unquantified caveat.

First, survivors were grouped by the a priori expected cardiotoxicity of standard treatment for their reported primary site, an indirect, site-level, ecological proxy for an individual-level treatment exposure that was never observed. The high-expected-cardiotoxicity group is breast, hematologic/blood, leukemia, or lymphoma, and prostate. The low-expected-cardiotoxicity group is non-melanoma or unknown-type skin, melanoma, thyroid, and cervix. Every other site is moderate/unclassified and is excluded from the high-versus-low contrast, because its treatment-cardiotoxicity profile is genuinely mixed at this level of site granularity. Supplementary File S3 lists the sites in each group with the treatment rationale for the assignment. This contrast is reported as *suggestive*, never confirmatory. Prostate is the weakest assignment in the high group, because androgen-deprivation therapy is common but not universal, and it is flagged again at every point at which its results are discussed (Section 3.7 and Section 4.4).

Second, two negative controls were built in the examined adult population, following the standard negative-control logic [12,13]. The negative-control outcome is accidental (external-cause) death under NCHS code 004, which has no plausible mechanistic link to cancer or its treatment. If survivor status is nonetheless associated with it, that association cannot reflect a cancer-specific pathway and instead signals general frailty, comorbidity, or differential healthcare and surveillance contact contaminating the survivor-versus-non-survivor contrast. The negative-control exposure is the group of 1038 participants whose only reported malignancy was non-melanoma or unknown-type skin cancer, the same group the analytic cohort excludes in Section 2.3. They reach the cancer question by the same self-report pathway as the cohort, with little cancer mortality and no cardiotoxic treatment, so their mortality contrast against participants with no reported cancer estimates what the survivor contrast would show in the absence of a cancer-specific pathway.

Both controls were fit in one model per outcome, with indicators for the analytic survivor cohort and for the skin-cancer-only group entered together and participants with no reported cancer as the reference, in a design-weighted penalized Cox model. The survivor indicator uses the cohort definition of Section 2.3 rather than the broader MCQ220 answer, so the exposure contrasted here is the one the rest of the paper reports. Each outcome was fit twice, adjusted for age and sex alone and adjusted for the demographic, socioeconomic, behavioral, and comorbidity covariates of Section 2.5. The laboratory panel is deliberately excluded from these models, since *C*-reactive protein and the other assays are measured in a subset of cycles and would shrink the control population without bearing on frailty or surveillance confounding (Supplementary File S3).

With both controls in the same fit, the difference between the two log hazard ratios is a calibrated survivor contrast: the part of the survivor-versus-no-cancer association that the skin-cancer-only group does not reproduce. The difference was computed inside every jackknife replicate, so its confidence interval is design-based rather than assembled from two separately estimated intervals (Supplementary File S3). This is the negative-control calibration logic of empirical calibration against control exposures [17], applied here to one control exposure rather than to a distribution of them, and it is reported as an adjustment to the contrast, not as a causal effect (Table S4).

### 2.7. Statistical Models: Fine–Gray Competing-Risks Regression

The Fine–Gray subdistribution hazard was fit by the data-transform route that R’s survival::finegray() followed by coxph() uses, which is mathematically equivalent to Fine and Gray’s original construction [18]: a subject who fails from the *competing* event is kept in the risk set past their own event time, down-weighted by the inverse probability of still being under observation at each later time, estimated from the reverse Kaplan–Meier distribution of censoring times [19]. The resulting long-format risk table was fit with a time-varying Cox model weighted by the product of that inverse-probability-of-censoring weight and the NHANES survey weight. Supplementary File S3 records the package versions and why this route was used. Every model reported in this article was fit in Python, not in R; R names the route because that is where it is documented. Section 2.16 gives the version of Python and of every package used.

Two covariate specifications were fit for each of the two primary outcomes, cardiovascular death and cancer death. Each was modeled with the other cause plus other-cause death collapsed as the competing event, per the subdistribution-hazard convention. The subdistribution hazard describes cumulative incidence rather than the rate among those still alive and event-free, and the two answer different questions about a population in which causes compete [20]. The primary specification is a fully adjusted, 25-covariate model including every covariate listed in Section 2.5. Ten of its covariates are reported as headline covariates: age, sex, time since diagnosis, hypertension, diabetes, *C*-reactive protein, body-mass index, number of cancer sites, the comorbidity index, and smoking status. A leakage pre-flight check (Table S5) confirmed that no retained covariate is algebraically or definitionally downstream of the outcome. Section 3.3 reports the ten headline covariates and Table S6 reports all 32 coefficients of the same model. As a specification check, a parsimonious model containing only the ten headline covariates was fit on the same complete-case sample, so the two specifications differ only in adjustment (Section 3.3).

Every Fine–Gray fit carried a ridge penalty of 0.5 on the internally standardized coefficients, which stabilizes the weighted time-varying fit and shrinks estimates toward the null. Refitting the primary model with a near-zero penalty of 0.01 changed the headline log hazard ratios by at most 0.08, so the shrinkage is small relative to the reported intervals.

The ten headline coefficients of each model and outcome were corrected for multiple comparisons by Holm–Bonferroni, with the adjusted *p*-value reported (Section 3.3).

### 2.8. Delayed Entry on the Time-Since-Diagnosis Scale

Survivors enter this cohort at the MEC examination, a median of eight years after the reported diagnosis, so the cohort is left-truncated: people who died between diagnosis and the examination could not be sampled, and the sample is enriched for indolent disease and for long survival [21,22]. Entering time since diagnosis as a covariate does not correct that selection. The models of Section 2.7 therefore measure time from the examination, which is the interval over which the survey observes anyone, and a second set of models measures time since diagnosis with delayed entry at the examination.

In the delayed-entry models each participant is at risk from their time since diagnosis at the examination until that value plus their observed follow-up. Risk sets contain only participants whose entry time precedes the event time, and the inverse-probability weights of the Fine–Gray construction use the product of the censoring survival function and the entry distribution, both estimated from the left-truncated data, as the Fine–Gray model under left truncation requires [19]. Time since diagnosis is the time scale in these models and so is not also a covariate; every other covariate of the primary specification is retained. Two further analyses restrict the sample to survivors examined within five years of diagnosis, where the truncation window is shortest: the headline covariates for both causes, and the cardiotoxicity contrast of Section 2.6, which is the estimate most exposed to this selection, since the high-cardiotoxicity sites are also those with the most treatment-related mortality soon after diagnosis. Table S7 reports the subsets analyzed and the distribution of entry times, exit times, and weights.

### 2.9. Relative Survival Against Internally Modeled Expected Mortality

Attributing a death to cancer or to cardiovascular disease is error-prone among people with cancer, and the public-use file substitutes synthetic causes for some records (Section 2.4). An excess-mortality analysis uses no cause-of-death information at all, so the two approaches bound each other. Expected mortality was modeled internally in the 47,482 examined adults with no reported cancer rather than taken from an external life table, so that the comparison population comes from the same survey under the same design. It was estimated on the attained-age scale with delayed entry at the age at examination, under two specifications: a demographic model, and one that adds body-mass index, hypertension, and diabetes (Supplementary File S3).

Three quantities follow at 5 and 10 years: observed all-cause survival in the cohort, the ratio of observed to expected survival, and Pohar–Perme net survival, which weights each person’s contribution by the inverse of their expected survival and is the consistent estimator of net survival when censoring by other causes is informative [23,24]. The excess mortality implied by net survival is then read against the cumulative incidence of recorded cancer death at the same horizon, which does use cause of death. Agreement between the two bounds how far the cause-specific results of this paper can be driven by cause-of-death misattribution. Table S8 reports both expected-mortality specifications.

### 2.10. Gradient-Boosted Competing-Risks Ensemble and SHAP

As a second, model-agnostic view of the same question, a cause-specific gradient-boosted ensemble (XGBoost [25], survival:cox objective) was fit separately for cardiovascular and for cancer death on the 25-covariate panel under five-fold cross-validation with a fixed seed. This is a cause-specific hazard model, in which the competing cause is censored at its own event time, and therefore a different estimand from the Fine–Gray subdistribution hazard of Section 2.7; pairing the two is an explicitly labeled triangulation, not a substitution of one for the other [11,26]. Discrimination was summarized by a survey-weighted Harrell’s *C*-index on the out-of-fold risk scores. Global and stratified SHapley Additive exPlanations (SHAP) [27,28] were computed on the model fit to the covariate-complete panel, by cancer site and by time since diagnosis, with every stratum’s cell size reported beside its attribution and a pre-specified minimum of 50 subjects for a stratified decomposition to be reported at all. Hyperparameters, the rank-stability check, and the stratification thresholds are given in Supplementary File S3.

### 2.11. Calibration, Decision Curves, and Simple-Baseline Benchmarks

For the cardiovascular-death gradient-boosted score, calibration and net benefit were assessed at a fixed 10-year horizon on a pseudo-binary outcome: cardiovascular death within 10 years counted as an event, event-free follow-up of at least 10 years as a non-event, and participants censored or removed by a competing event earlier were excluded, since their 10-year status is not knowable without machinery not built here. The score was Platt-recalibrated, binned into deciles for a reliability table, and summarized with the Brier score and a decision-curve analysis of net benefit against treat-all and treat-none strategies across threshold probabilities 0.01 to 0.50 [29,30]. The exclusion rule and the bootstrap settings are given in Supplementary File S3.

The ensemble in Section 2.10 uses the full 25-covariate panel. Age is the dominant determinant of cardiovascular death in any adult population, so a discrimination figure from that panel cannot be interpreted unless it is read against what age alone achieves. We therefore refit the same cause-specific gradient-boosted pipeline on two reduced feature sets, age alone and age with sex, with the same five-fold cross-validation procedure, hyperparameters, and random seed, and compared both with the full panel for each cause.

Two quantities are reported for each cause: each model’s survey-weighted *C*-index with a design-aware jackknife interval, and the paired difference between the full panel and each reduced baseline, which is the inferential comparison of the two. Supplementary File S3 gives the rule that makes the difference a paired quantity on a common estimable sample.

### 2.12. Complex-Survey Design and Design-Aware Confidence Intervals

NHANES is a complex, multistage probability sample, and its design was applied to every estimator reported in this article, including the machine-learning discrimination and calibration metrics and not only the descriptive statistics, using the masked primary-sampling-unit identifier SDMVPSU, the masked stratum identifier SDMVSTRA, and the two-year MEC examination weight WTMEC2YR divided by ten for the pooling of ten cycles, per NCHS pooling guidance. Every regression coefficient, hazard ratio, survival estimate, and *C*-index reported with a design-aware interval used a stratified delete-one-primary-sampling-unit jackknife, in which every masked PSU is dropped once and the remaining PSUs in its stratum are up-weighted by *n*h/(*n*h − 1), with a *t* interval on degrees of freedom equal to the number of jackknife units minus the number of strata. The Fine–Gray inverse-probability-of-censoring weight is itself a function of the survey design, so design-aware Fine–Gray intervals were obtained by refitting the entire transform-plus-regression pipeline once per replicate rather than by reweighting a fixed point estimate. Supplementary File S3 records why replicate percentiles were not used and why no fasting-subsample weight was required.

### 2.13. Model Diagnostics: Leakage Audit, Multicollinearity, and Multiple Comparisons

Every candidate covariate was screened for target leakage, meaning algebraic or definitional downstream-ness relative to the outcome, before any model was fit (Table S5). Age at diagnosis, follow-up time, the mortality-status flag, the raw cause-of-death code, and the two cause-specific death flags were excluded as components or restatements of the outcome, and the fine-grained site variables were reserved for stratification. Variance-inflation factors on the standardized design matrix of the covariate-complete sample reached a maximum of 3.32, under the conventional flag threshold of 5, so multicollinearity was not a material concern for any reported coefficient. Multiple-comparison correction (Holm–Bonferroni) was applied as described in Section 2.7.

### 2.14. Missing Data: Multiple-Imputation Sensitivity Analysis

NHANES did not measure *C*-reactive protein in 2011–2012 or 2013–2014. Every model is complete-case and *C*-reactive protein is in the fully adjusted panel, so no participant from those two cycles enters the fully adjusted models, which therefore cover eight of the ten pooled cycles; Section 3.11 reports a sensitivity analysis that drops *C*-reactive protein and recovers all ten. Listwise deletion on the fully adjusted panel retained 2225 of 3826 survivors. On a compact 11-covariate set used for this check, listwise deletion dropped 33.2% of the cohort, above the 10% threshold set for a multiple-imputation sensitivity analysis, so multiple imputation by chained equations with Rubin’s-rules pooling [31,32,33] was run for a compact model of each cause-specific death indicator and is reported as a check on the *direction and magnitude* of listwise-deletion bias, not as a replacement for the design-weighted primary analysis (Section 3.11). The imputation settings and the covariate list are given in Supplementary File S3.

### 2.15. Decomposition of the Comorbidity Index

Section 2.5 states that five of the comorbidity index’s seven components are themselves prior cardiovascular diagnoses. This section measures that rather than asserting it. We refit the primary Fine–Gray models under three specifications differing only in how comorbidity enters, with every other covariate, the survey design, and the ridge penalty (Section 2.7) held fixed.

The first specification is the primary one, carrying the seven-component index alongside the separate hypertension and diabetes covariates. The second replaces the index with a count of its five cardiovascular components alone, namely congestive heart failure, coronary heart disease, angina, myocardial infarction, and stroke, leaving hypertension and diabetes as separate covariates. The third drops the index entirely, leaving only those two separate covariates.

The second specification isolates what the cardiovascular components carry on their own. The third shows what remains when no cardiovascular count is in the model at all. A deliberate property of this design is that neither of the two new specifications enters hypertension or diabetes twice. The primary specification does, because both sit inside the index and are also entered separately, and Section 4.2 returns to what that implies for reading their coefficients.

### 2.16. Software, Reproducibility, and Pipeline Release

All analyses were implemented in Python 3.13.12 using pandas 2.2.3, numpy 2.4.2, lifelines 0.30.3 (Cox time-varying fitter for the Fine–Gray transform), xgboost 3.2.0 (gradient-boosted competing-risks ensemble), shap 0.48.0 (SHAP attribution), scikit-learn 1.6.1 (Platt calibration, cross-validation folds), statsmodels 0.14.6 (MICE, Holm–Bonferroni), matplotlib 3.10.8 (figures), scipy 1.16.3 (distribution functions), and pyreadstat 1.3.3 (reading the NHANES transport files). No reagents, devices, instruments, or commercial materials were used in this study: every measurement analyzed here was made by NCHS and released in the NHANES public-use files. A fixed random seed (42) was used for every stochastic step: cross-validation fold assignment, the gradient-boosted ensemble, the SHAP bootstrap rank-stability check, and multiple imputation. The full pipeline covers NHANES ingestion and harmonization, cohort and outcome construction, covariate engineering, Fine–Gray and gradient-boosted model fitting, design-aware jackknife confidence intervals, SHAP analysis, the cardiotoxicity and negative-control probes, and table generation. It is released under the MIT license and provided in full, as a formatted source-code listing, in Supplementary File S1.

## 3. Results

### 3.1. Cohort Characteristics

The analytic cohort comprised 3826 cancer survivors (Table S1), 3825 of whom (100.0%) had a matched Linked Mortality File record. Table 1 and Table 2 report design-weighted baseline characteristics stratified by the four-level cause-specific outcome. Participants who later died of heart disease were markedly older at baseline (median 78.0 years, interquartile range 71.0 to 80.0, against 60.0 years, 48.0 to 70.0, among those alive or censored) and carried a higher comorbidity-index burden (2.0, 1.0 to 3.0, against 1.0, 0.0 to 1.0), a higher systolic blood pressure (136.1 mmHg, 121.6 to 153.8, against 123.3 mmHg, 112.7 to 136.0), more hypertension (70.0% against 46.2%) and diabetes (26.3% against 14.2%), and a lower proportion of women (51.4% against 66.3%). Participants who died of cancer were younger than the cardiovascular-death group (median 70.0 years, 62.0 to 79.0), had a shorter median time since diagnosis (6.0 years, 2.0 to 12.3, against 9.0 years, 4.0 to 17.0, among those alive or censored), which is consistent with cancer death concentrating earlier after diagnosis, and reported more distinct cancer sites (upper quartile 2.0 against 1.0, with medians equal). *C*-reactive protein was higher in every death group than among those alive or censored (2.8 mg/L, 1.3 to 6.0, for cardiovascular death, 3.4, 1.5 to 7.0, for cancer death and 3.2, 1.3 to 7.2, for other-cause death, against 2.0, 0.9 to 4.7), the direction expected if systemic inflammation tracks mortality risk. Breast, gynecologic, and prostate cancer were the three largest reported primary-site groups (20.1%, 18.7% and 12.8% of the cohort, design-weighted), with prostate and colorectal cancer over-represented among cardiovascular deaths (19.8% and 14.9%) and hematologic cancer among cancer deaths (10.3% against 5.0% of the cohort).

### 3.2. Cause-Specific Mortality Outcomes

Table 3 summarizes the cause-specific mortality outcome. Of the 3826 survivors, 3825 (100.0%) linked to the Linked Mortality File, and 1416 (37.0%) had died by the end of 2019 follow-up. Heart disease accounted for 288 deaths (7.5% of the cohort), cancer for 497 (13.0%), and other causes for 631 (16.5%), leaving 2410 (63.0%) alive or administratively censored. The lower block of Table 3 shows what the outcome definition does. Counting cerebrovascular death as cardiovascular, as a pooled analysis of all ten cycles would, raises the count from 288 to 351 and lowers other-cause deaths from 631 to 568, but every one of those 63 additional deaths falls in 1999 to 2014, because the last two public-use releases do not code cerebrovascular death separately (Table S2). Restricted to the eight cycles in which both definitions are identifiable, the fully adjusted model gives a comorbidity-index hazard ratio of 1.39 (95% CI: 1.21–1.60) for heart disease alone and 1.38 (1.22–1.56) for heart or cerebrovascular death, and no headline covariate changes direction (Table S3). The wider definition is therefore not wrong in those cycles; it is unavailable in the last two, and pooling it across all ten would make the outcome a function of survey cycle.

**Figure 2:**
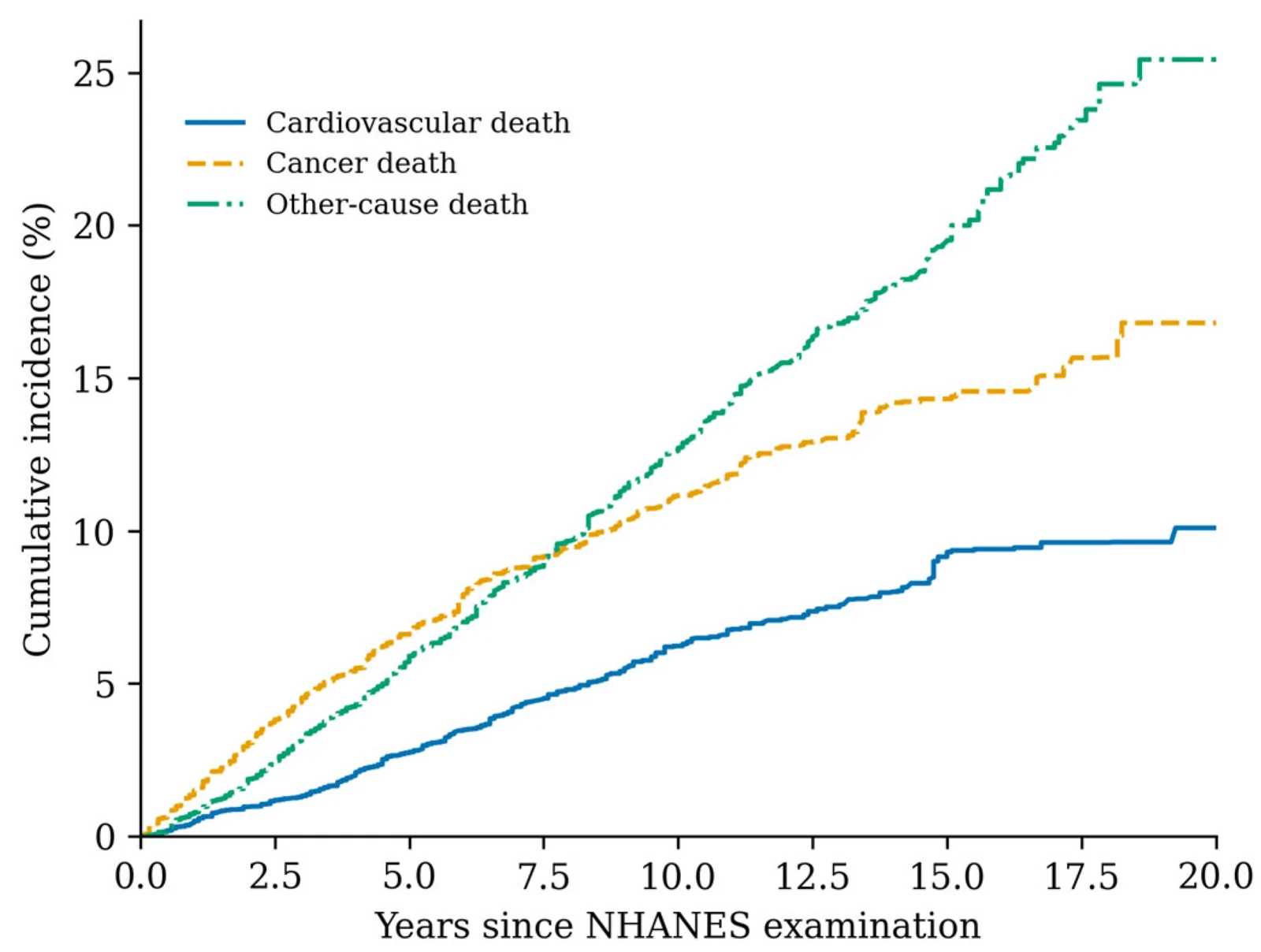
Survey-weighted Aalen–Johansen cumulative incidence of cardiovascular death, cancer death, and other-cause death among cancer survivors, treating the three causes as competing events. Cumulative incidence rather than 1 − Kaplan–Meier: with three mutually exclusive causes the cause-specific complement of the survival function would overstate every curve.

Figure 2 shows the survey-weighted cumulative incidence of each cause over follow-up, estimated by the Aalen–Johansen estimator so that the three causes compete rather than censoring one another. Cancer death accumulated faster than cardiovascular death throughout, and the two curves did not converge across the follow-up window: at 10 years the weighted cumulative incidence was 7.5% for cardiovascular death against 11.1% for cancer death, and at 20 years 12.6% against 16.8%, with other-cause death highest at both horizons (11.4% and 22.9%). This is the descriptive counterpart of the divergent covariate associations in Section 3.3. In this cohort, cancer death did not give way to cardiovascular death within the follow-up window. The registry crossover [2] was confined to survivors aged 80 years or older at diagnosis, and this cohort is younger, so the two findings do not conflict.

### 3.3. Fine–Gray Competing-Risks Regression: Different Predictors for Each Cause

The complete-case sample for both Fine–Gray specifications and for the gradient-boosted ensemble (Section 3.8) was *n* = 2225 (173 cardiovascular, 278 cancer, and 385 other-cause deaths; 1389 censored). Table 4 reports the ten headline covariates of the primary, fully adjusted model, with design-aware jackknife confidence intervals (238 jackknife units, 118 strata, 120 degrees of freedom) and Holm–Bonferroni-adjusted *p*-values for both outcomes, and beside them the same covariates from the parsimonious model. In the primary model two associations survived correction for cardiovascular death: age (HR 1.08, 95% CI: 1.06–1.10; *p* < 0.001) and the comorbidity index (HR 1.40, 95% CI: 1.24–1.59; *p* < 0.001). Four survived correction for cancer death: age (HR 1.03, 95% CI: 1.01–1.05; *p* = 0.012), female sex (HR 0.50, 95% CI: 0.34–0.74; *p* = 0.005), never having smoked (HR 0.48, 95% CI: 0.32–0.72; *p* = 0.004), and the number of reported cancer sites (HR 2.18, 95% CI: 1.54–3.08; *p* < 0.001). The comorbidity index was not associated with cancer death after correction (HR 0.89, 95% CI: 0.80–1.00; *p* = 0.288). The comorbidity index’s association with cardiovascular death is this study’s main substantive result, and Section 4.2 examines what it can and cannot mean, since five of the index’s seven components are themselves prior cardiovascular diagnoses (Section 2.5).

The parsimonious model reproduces the headline pattern (Table 4). The comorbidity index’s cardiovascular-death association is unchanged (HR 1.43, 95% CI: 1.28–1.59), and age, female sex, never having smoked, and the number of cancer sites keep their direction for cancer death. Two estimates move. Female sex is weaker for cancer death without the fuller adjustment (0.64, 95% CI: 0.47–0.85), and *C*-reactive protein reaches significance for cancer death only in the parsimonious model (1.02, 95% CI: 1.01–1.04, against *p* = 0.204 in the primary model), so that association is not robust to adjustment for the fuller covariate panel. Table S6 reports all 32 coefficients of the primary model.

**Figure 3:**
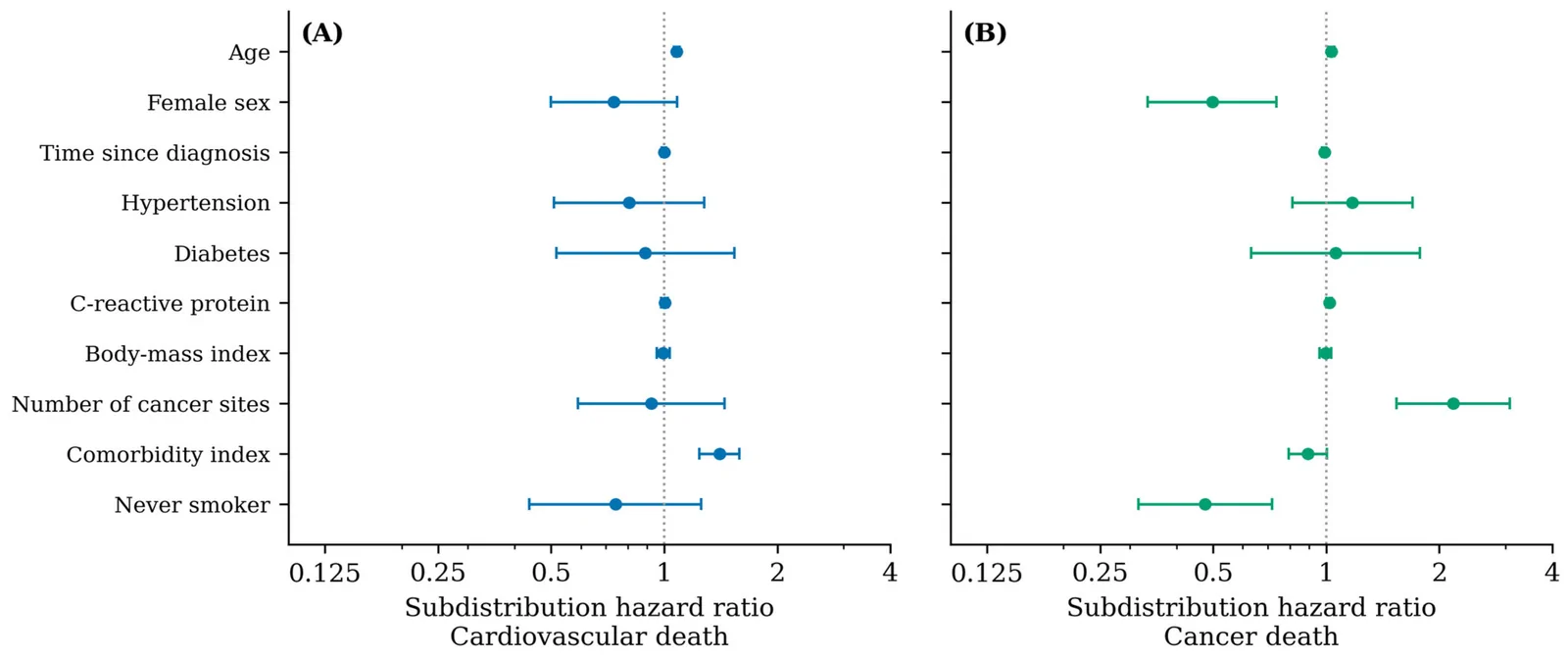
Fine–Gray subdistribution-hazard ratios with 95% design-based confidence intervals from the stratified delete-one-PSU jackknife, for (**A**) cardiovascular death and (**B**) cancer death. The axis is logarithmic, so that reciprocal hazard ratios are equidistant from the null. The dotted vertical reference marks a hazard ratio of 1.

Figure 3 plots the primary model’s headline hazard ratios for both causes side by side on a common logarithmic axis. The comorbidity index lies above the null for cardiovascular death and its interval reaches the null for cancer death. The number of reported cancer sites shows the opposite arrangement, above the null for cancer death only. Most other covariates have intervals that span the null in both panels, as expected in a complete-case sample of 2225 with 173 cardiovascular and 278 cancer deaths.

### 3.4. Delayed Entry on the Time-Since-Diagnosis Scale Leaves the Headline Pattern Intact

A computable time since diagnosis and follow-up after the examination were available for 3771 of the 3773 survivors with a recoverable age at diagnosis, of whom 2224 were covariate-complete (173 cardiovascular, 277 cancer, and 385 other-cause deaths). Their entry times, measured from the reported diagnosis, had a median of 8 years (interquartile range 4 to 16) and ran to 78 years, and their exit times a median of 17.7 years, so the cohort observes a window that opens, on average, eight years after the event that defines membership in it. The inverse-probability weights of the left-truncated Fine–Gray construction had a median of 0.96 and a maximum of 12.1 (Table S7).

Refitting on that time scale leaves the headline pattern intact (Table 5). For cardiovascular death the comorbidity index gives 1.41 (95% CI: 1.22–1.64), against 1.40 (1.24–1.59) on the examination time scale, and age 1.07 (1.05–1.09). For cancer death the number of reported cancer sites gives 2.64 (1.85–3.78), never having smoked 0.51 (0.33–0.78), female sex 0.62 (0.42–0.90) and age 1.03 (1.01–1.05). One estimate strengthens: the comorbidity index for cancer death moves from 0.89 (0.80–1.00) to 0.86 (0.76–0.98), so on the diagnosis time scale a higher comorbidity count is associated with a lower subdistribution hazard of cancer death. The substantive reading of the paper does not depend on which time scale is used, which is the question delayed entry was fitted to answer.

Restricting to the 813 survivors examined within five years of diagnosis, where the truncation window is shortest (68 cardiovascular and 120 cancer deaths), strengthens rather than weakens the headline results: the comorbidity index gives 1.66 (95% CI: 1.23–2.24) for cardiovascular death and the number of cancer sites 2.99 (1.53–5.84) for cancer death, with never having smoked at 0.48 (0.26–0.91). The cardiotoxicity contrast behaves differently. Under delayed entry on the full sample it is 0.46 (0.27–0.77), further from the null than the 0.55 (0.33–0.91) of the examination time scale, and in the 520 recently diagnosed survivors of high- or low-cardiotoxicity sites, with 45 cardiovascular deaths, it is 0.66 (0.31–1.42). The point estimate stays below one in every specification and the restricted interval is too wide to decide anything, so left truncation does not account for the direction of that contrast, and the recently diagnosed subset cannot settle its size (Section 4.4).

### 3.5. Relative Survival and Cause-of-Death Attribution

Expected mortality was estimated in the 43,025 examined adults with no reported cancer who were complete on the demographic covariate set, among whom 5826 died during follow-up. Applied to the 3473 survivors complete on the same set, it gives the comparison in Table 6. At 5 years observed all-cause survival was 0.849 (95% CI: 0.835–0.863) against an expected 0.896 (0.892–0.901), a relative survival of 0.947 (0.933–0.961). At 10 years observed survival was 0.701 (0.682–0.720) against an expected 0.771 (0.761–0.780), a relative survival of 0.909 (0.889–0.930).

Pohar–Perme net survival, which weights each person by the inverse of their own expected survival, was 0.945 (0.930–0.960) at 5 years and 0.897 (0.869–0.924) at 10. The implied excess mortality at 10 years is therefore 0.103, and the cumulative incidence of recorded cancer death at the same horizon is 0.115 (0.102–0.127). Those two quantities are estimated from different information: the first uses vital status and the expected rates of people with the same demographics, while the second uses the cause written on the death record. They agree to within about one percentage point, which bounds how much the cause-of-death results of this paper can be driven by misattribution or by the synthetic substitutions in the public-use file. Adding body-mass index, hypertension, and diabetes to the expected-mortality model widens the 10-year net survival to 0.947 (0.877–1.016), which is the expected consequence of conditioning expected mortality on the covariates that also predict excess mortality; the demographic model is reported as primary for that reason (Table S8).

### 3.6. Negative-Control Outcome and Negative-Control Exposure

Any survivor-versus-non-survivor mortality contrast in these data can be confounded by general frailty, by comorbidity unrelated to cancer, or by differential healthcare contact, rather than reflecting a cancer- or treatment-specific pathway (Section 2.6). Two controls measure that. In the examined adult population of 52,398, the analytic survivor cohort (3826), the skin-cancer-only group (1038) and the 47,482 participants reporting no cancer were entered in one model per outcome, with the no-cancer group as the reference (Table 7, full table in Table S4).

For accidental death, the negative-control outcome was restricted to the eight cycles that code it separately (*n* = 34,141 covariate-complete, 199 accidental deaths), and survivors had a higher hazard than participants with no reported cancer (HR 1.78, 95% CI: 0.89–3.54); so did the skin-cancer-only group (HR 2.01, 95% CI: 0.87–4.66). Neither interval excludes 1 once the full covariate set is adjusted for; under age and sex alone the survivor estimate is 1.77 (0.98–3.19) and the skin-cancer-only estimate 1.59 (0.72–3.49). The point estimates are what matter here: an outcome that no cancer mechanism can produce is raised by about three-quarters in both cancer-reporting groups, which is the signature of frailty or surveillance confounding rather than of cancer.

For cardiovascular death (*n* = 42,728 covariate-complete, 1700 deaths from heart disease), survivors had a *lower* adjusted hazard than participants with no reported cancer (HR 0.84, 95% CI: 0.71–1.00), and the skin-cancer-only group was almost identical (HR 0.87, 95% CI: 0.65–1.18). The calibrated contrast, the difference of the two log hazard ratios computed inside every jackknife replicate, is 0.96 (95% CI: 0.70–1.32). A negative-control exposure that shares the reporting and surveillance pathway of the cohort, with little cancer mortality and no cardiotoxic treatment, therefore reproduces the survivor contrast almost exactly, and what remains after calibration is indistinguishable from no contrast at all. The same calibration for accidental death leaves 0.88 (0.33–2.33). Taken together, the two controls say that the survivor-versus-no-cancer comparison in these data is not measuring a cancer-specific pathway, and that the part of it which is cancer-specific cannot be separated from selection and surveillance with a survey of this design.

### 3.7. Cardiotoxicity Site-Level Contrast

Among the 2368 survivors whose reported primary site fell into the high- or low-expected-cardiotoxicity groups (Section 2.6), 1403 had complete data on the cardiotoxicity-contrast covariate set (930 high-expected-cardiotoxicity, 473 low-expected-cardiotoxicity; 105 cardiovascular deaths in this subset). The design-aware Fine–Gray hazard ratio for high- versus low-expected cardiotoxicity was 0.55 (95% CI: 0.33–0.91; 228 jackknife units, 113 strata, 115 degrees of freedom). It points in the opposite direction from the treatment-cardiotoxicity hypothesis: survivors of high-expected-cardiotoxicity sites had a lower, not higher, relative hazard of cardiovascular death. A time-since-diagnosis interaction probe found no evidence of a delayed-onset signal emerging with longer follow-up (interaction hazard ratio 1.00, 95% CI: 0.96–1.03), and in that model the main contrast was 0.57 (0.28–1.14). Table 8 reports both models together with the VanderWeele–Ding E-value [34,35] for the main contrast: 3.04 at the point estimate and 1.44 at the confidence bound closer to the null. A confounder associated with both site group and cardiovascular death by a risk ratio of 1.44 on each count would be enough to move the interval across the null. This contrast compares cancer sites, not treatments, and the sites differ in age, sex and baseline cardiovascular risk (Section 4.4); it is not evidence that cardiotoxic treatment protects the heart. Prostate cancer’s inclusion in the high-cardiotoxicity group is the weakest of its three members (Section 2.6), so any interpretation should further discount the prostate-driven share of it. Section 3.4 reports the same contrast under delayed entry.

### 3.8. Ensemble Discrimination, Calibration, and Simple Baselines

On the same *n* = 2225 covariate-complete panel, the survey-weighted gradient-boosted cause-specific ensemble discriminated cardiovascular death better (*C*-index 0.859, 95% CI: 0.830–0.889) than cancer death (*C*-index 0.746, 95% CI: 0.717–0.775). Most of that discrimination is carried by age and sex, as the baseline comparison below shows. For the 10-year cardiovascular-death horizon the Platt-recalibrated risk score tracked observed risk in the top decile (mean predicted 0.400, observed proportion 0.467, *n* = 90) and overestimated it in the lowest three (mean predicted about 0.07 against observed proportions of 0.000 to 0.011), summarized by a Brier score of 0.077 (95% CI: 0.063–0.091, from an independent-row bootstrap that ignores the survey design and so understates uncertainty). Net benefit exceeded both default strategies from a threshold probability of 0.08 upward, and equaled treat-all below it, because every risk score in this cohort lies above those low thresholds. Table S9 reports calibration by decile and net benefit at selected thresholds.

**Figure 4:**
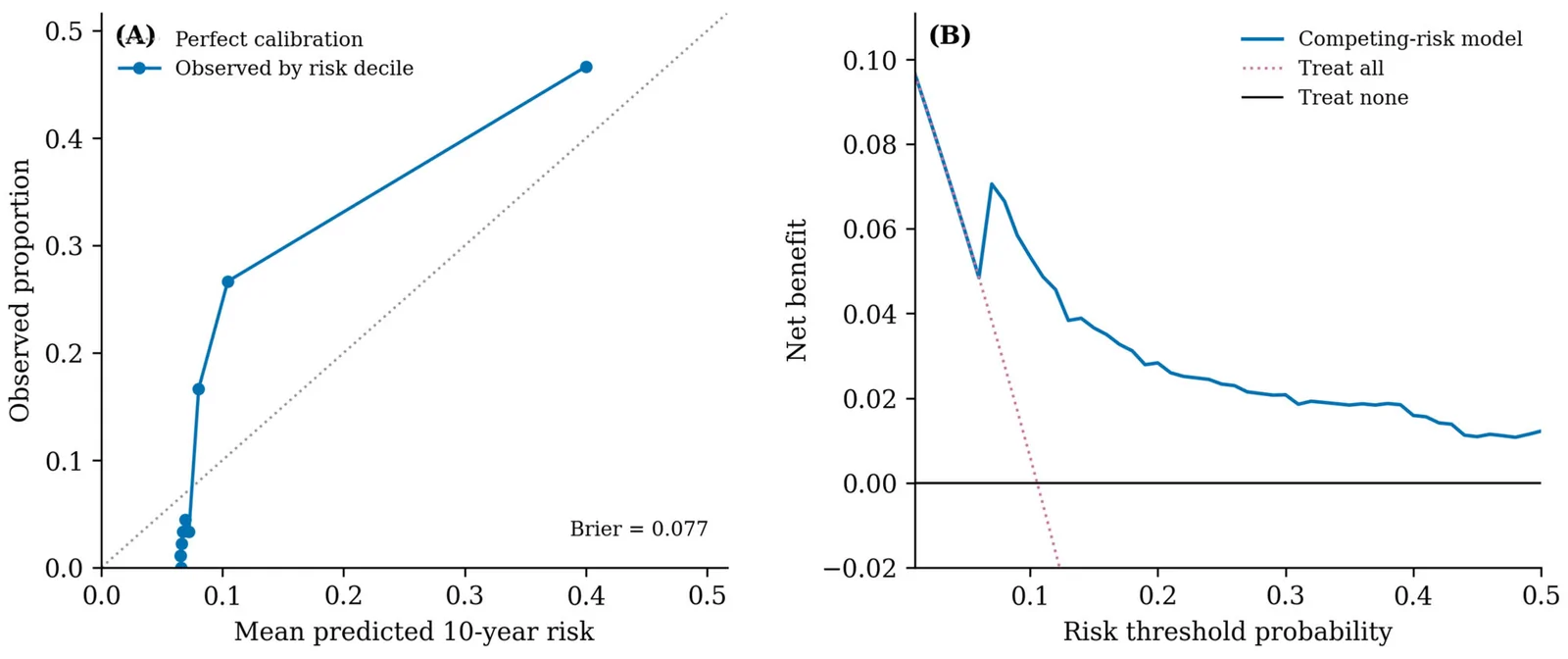
(**A**) Calibration of the 10-year cardiovascular-death model by risk decile, showing observed by risk decile against mean predicted risk, with the perfect calibration diagonal for reference and the Brier score inset. (**B**) Decision-curve analysis: Net benefit of the competing-risk model against the treat-all and treat-none default strategies across risk thresholds.

Figure 4 shows both diagnostics. Panel (A) makes the calibration pattern legible: the model tracks the diagonal closely only in the top decile and overestimates risk systematically in the bottom three, which is the limitation a reader should carry into any applied use of the score. Panel (B) shows the corresponding net-benefit crossover.

Read against what age alone achieves, the ensemble’s discrimination is a modest increment. On the 2225 subjects estimable under all three models, age alone gave a survey-weighted *C*-index of 0.818 (95% CI: 0.781–0.855) for cardiovascular death and age with sex 0.824 (95% CI: 0.789–0.859), against 0.859 (95% CI: 0.830–0.889) for the full 25-covariate ensemble. For cancer death the same three models gave 0.662 (95% CI: 0.627–0.696), 0.689 (95% CI: 0.654–0.724) and 0.746 (95% CI: 0.717–0.775). The paired differences, which are the inferential comparison, were 0.042 (95% CI: 0.018–0.065) against age alone and 0.036 (95% CI: 0.013–0.059) against age with sex for cardiovascular death, and 0.085 (95% CI: 0.052–0.117) and 0.057 (95% CI: 0.025–0.089) for cancer death. All four intervals exclude zero, so the covariate panel carries information about both causes beyond demography, and all four increments are small beside the discrimination that age alone already provides.

### 3.9. SHAP Attribution

SHAP feature-ranking stability across 30 bootstrap resamples was high for both cause-specific models (Spearman *ρ* = 0.999 for both causes), above the 0.95 threshold set in advance. Table S10 reports the ten globally most influential features by mean absolute SHAP value for each cause. Age dominated both models (mean absolute SHAP 1.159 for cardiovascular death, 0.511 for cancer death), consistent with its role in the Fine–Gray models. For cardiovascular death the comorbidity index ranked second (0.318), followed by the income-to-poverty ratio (0.261), race or ethnicity (0.235), and the neutrophil-to-lymphocyte ratio (0.182). For cancer death the ranking after age was female sex (0.235), *C*-reactive protein (0.191), smoking status (0.190) and the neutrophil-to-lymphocyte ratio (0.171).

**Figure 5:**
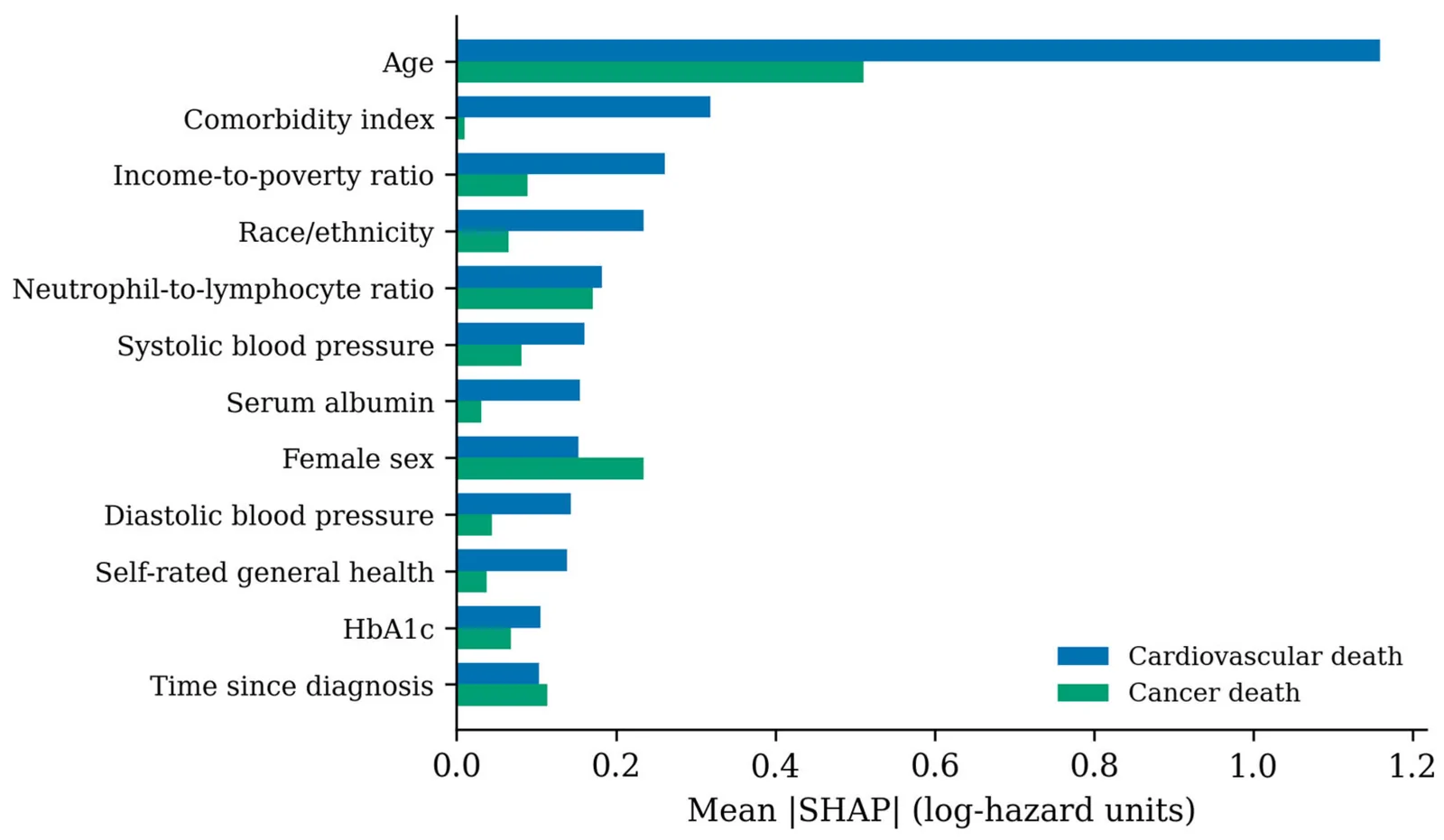
Mean absolute SHAP value per predictor in the gradient-boosted competing-risk models for cardiovascular death and cancer death, for the twelve predictors ranked highest by the cardiovascular model. Both series share that ordering so the two outcomes are read against each other rather than each against its own ranking.

Figure 5 shows the two rankings against each other on a shared ordering. The comorbidity index ranks second for cardiovascular death and near the bottom for cancer death. Mean absolute SHAP values carry no sign, so this says the index carries little information about cancer death, not that its association reverses, and it agrees with the Fine–Gray models from an entirely separate model class.

Stratified SHAP was run by cancer site and by time-since-diagnosis category. Table S11 reports every stratum’s full cell size beside the smaller subset that met the pre-specified minimum of 50 covariate-complete cases, and the two counts differ substantially: the breast-cancer stratum holds 714 survivors, of whom 388 are covariate-complete, and two site strata fall below the threshold and are reported without a decomposition.

### 3.10. The Comorbidity Index Is Its Cardiovascular Components

For cardiovascular death, the primary seven-component index gave a hazard ratio of 1.404 (95% CI: 1.243–1.586). Replacing it with a count of its five cardiovascular components alone gave 1.421 (95% CI: 1.251–1.615), with every other covariate unchanged. For cancer death, the primary index gave 0.894 (95% CI: 0.796–1.004) and the five-component count 0.890 (95% CI: 0.788–1.004).

The five cardiovascular components reproduce the full index. Dropping the two non-cardiovascular components changes the estimate by less than the width of either interval, for both causes. The association reported in Section 3.3 is therefore carried by prior cardiovascular diagnoses rather than by comorbidity burden in general, which is what Section 4.2 argues on construction grounds and what this specification measures directly.

The third specification exposes a second consequence. In the primary model hypertension and diabetes are entered twice, once inside the index and once separately, and their separate coefficients for cardiovascular death are 0.807 (95% CI: 0.510–1.279) and 0.891 (95% CI: 0.517–1.536), both below unity. With the index removed and no double entry, the same two covariates give 1.247 (95% CI: 0.830–1.873) and 1.329 (95% CI: 0.794–2.222). The direction reverses for both. Neither coefficient’s interval excludes unity, so no claim in this paper rests on them, but a reader scanning the coefficient table would otherwise be invited to read a protective association that the specification, rather than the cohort, produced.

### 3.11. Missing Data, Diagnostics, and the Analysis Without C-Reactive Protein

Listwise deletion on the compact 11-covariate imputation set dropped 33.2% of the survivor cohort (complete-case *n* = 2554 of 3826), well above the 10% threshold set for a multiple-imputation sensitivity check. Table S12 compares complete-case and MICE/Rubin-pooled cause-specific coefficients for both causes. Two coefficients changed sign under imputation: the number of cancer sites for cardiovascular death (*β* = −0.010 against 0.019) and systolic blood pressure for cancer death (0.001 against −0.001). Both are close to zero with standard errors several times their size in either fit. The coefficients that are clearly non-zero kept their direction and size: age for both causes, and female sex, *C*-reactive protein and the number of cancer sites for cancer death (for the number of sites, complete-case *β* = 0.662 against MI-pooled 0.720). The comorbidity index is not in this compact set, so the imputation check says nothing about it. MICE assumes data missing at random, so this comparison supports, without proving, that listwise deletion does not overturn the direction of the clearly non-zero associations, even though it reduces the sample available to the primary design-weighted models (Section 3.3).

All design-based intervals in this article use the full delete-one-primary-sampling-unit jackknife, in which every eligible unit is deleted once; estimating the variance from a subset of strata understates it. The maximum variance-inflation factor across the covariate-complete design matrix (*n* = 2225) was 3.32, for one of the race or ethnicity indicators, well under the conventional threshold of 5 (Section 2.13, Table S5), so multicollinearity does not materially affect the interpretation of any reported hazard ratio.

NHANES did not measure *C*-reactive protein in 2011–2012 or 2013–2014, and the fully adjusted models are complete-case, so the analytic sample of Section 3.3, Section 3.4, Section 3.5 and Section 3.6 contains no participant from those two cycles (Section 2.14). Refitting the same specification without *C*-reactive protein restores them. The complete-case sample rises from 2225 to 2793 and covers all ten cycles, gaining 259 participants from 2011 to 2012, 305 from 2013 to 2014, and four from the last two cycles, in which *C*-reactive protein was missing for a few participants who are otherwise covariate-complete, and the design gains units: 299 jackknife units across 147 strata, against 238 across 118.

The headline association does not depend on that restriction. Table S13 reports the ten headline covariates under both specifications. For cardiovascular death the comorbidity index gives 1.41 (95% CI: 1.26–1.58; Holm-adjusted *p* < 0.001) without *C*-reactive protein, against 1.40 (1.24–1.59) with it. For cancer death it remains non-significant, 0.91 (0.81–1.02) against 0.89 (0.80–1.00). Age, female sex, the number of reported cancer sites, and never having smoked keep their direction and their significance for cancer death. One estimate moves: female sex for cardiovascular death goes from 0.74 (0.50–1.08) to 0.66 (0.46–0.94), which still does not survive multiple-comparison correction (*p* = 0.145).

## 4. Discussion

### 4.1. Principal Findings

Among a nationally representative cohort of 3826 United States cancer survivors followed for cause-specific mortality, this study’s first result is a calibration rather than an association. Accidental death, an outcome with no plausible link to cancer or its treatment, was raised in the survivor cohort (HR 1.78, 95% CI: 0.89–3.54) and equally in the group whose only reported malignancy was a skin cancer (2.01, 0.87–4.66). For cardiovascular death the survivor contrast against participants with no reported cancer, 0.84 (0.71–1.00), is reproduced by that same control group, 0.87 (0.65–1.18), and the calibrated contrast is 0.96 (0.70–1.32). A survey comparison of survivors with people reporting no cancer therefore measures the reporting and surveillance pathway at least as much as anything cancer-specific. An indirect site-level cardiotoxicity proxy pointed the unexpected way, with a lower hazard of cardiovascular death for high-expected-cardiotoxicity sites (0.55, 0.33–0.91), and an E-value of 1.44 at the confidence bound leaves it fragile to modest confounding. These are first-order findings, and they leave the survivor-versus-non-survivor contrast indistinguishable from none. What these data do support is a comparison within the survivor cohort. A higher comorbidity index carries a higher subdistribution hazard of cardiovascular death, 1.40 (95% CI: 1.24–1.59), and that is the association this cohort is positioned to measure. Every association reported below is to be read against the calibration above.

Two design checks bound the two ways this cohort could mislead. Entry at the examination, a median of eight years after diagnosis, left-truncates the sample, but refitting on the time-since-diagnosis scale with delayed entry leaves the headline estimates where they were: the comorbidity index gives 1.41 (1.22–1.64) for cardiovascular death against 1.40 (1.24–1.59) on the examination scale, and the estimate rises to 1.66 (1.23–2.24) among survivors examined within five years of diagnosis. Cause of death is error-prone in this population and partly synthetic in the public-use file, but an excess-mortality analysis that uses no cause-of-death information agrees with the recorded cancer deaths, as Section 4.5 sets out.

The substantive associations follow. The comorbidity index was associated with a higher subdistribution hazard of cardiovascular death (HR 1.40, 95% CI: 1.24–1.59), surviving Holm–Bonferroni correction across the ten headline covariates, and a direct decomposition shows that association is carried by its five cardiovascular components rather than by comorbidity burden in general (Section 3.10). Its association with cancer death was not significant after correction (0.89, 0.80–1.00). Older age was associated with a higher hazard of both causes. Female sex and never having smoked were associated with a lower hazard of cancer death, and a greater number of reported cancer sites with a higher one. A gradient-boosted ensemble, triangulating the Fine–Gray result with a different estimand and model class, discriminated cardiovascular death better (*C*-index 0.859) than cancer death (0.746), and most of that discrimination is carried by age and sex (Section 3.8).

### 4.2. The Comorbidity Index Is Substantially a Cardiovascular-Diagnosis Count

Before the main substantive result is read as evidence that comorbidity burden in some general sense raises cardiovascular mortality, the index’s construction has to be explicit. Five of its seven components (congestive heart failure, coronary heart disease, angina, myocardial infarction, and stroke) are themselves prior cardiovascular diagnoses, and hypertension and diabetes, the remaining two, are also entered separately in every model (Section 2.5). A survivor with a high comorbidity index is, to a substantial degree, a survivor who already carries cardiovascular disease, so an association between the index and *subsequent* cardiovascular death is in part a statement that cardiovascular disease precedes cardiovascular death. Section 3.10 measures how much of it is exactly that.

Two things follow. A count of the five cardiovascular components alone reproduces the index’s hazard ratio for both causes with every other covariate held fixed (Section 3.10), so the narrower claim is the one these data support and the broader one is unnecessary rather than merely unproven. The primary specification also enters hypertension and diabetes twice, once inside the index and once as separate covariates, and that double entry pushes their separate coefficients below unity for cardiovascular death; removing the index reverses their direction. Neither coefficient’s design-aware interval excludes unity, so no claim in this paper rests on them, but a reader scanning the coefficient table would otherwise be invited to read a protective association for diabetes against cardiovascular death that is a property of the specification and not of the cohort.

### 4.3. What the Two Negative Controls Leave of the Survivor Contrast

The two control analyses of Section 3.6 are not peripheral robustness checks. Accidental death has no biological or treatment pathway connecting it to a cancer diagnosis, yet both cancer-reporting groups had a raised hazard of it: 1.78 (95% CI: 0.89–3.54) for the survivor cohort and 2.01 (0.87–4.66) for participants whose only reported malignancy was a skin cancer. The plausible explanations are general frailty, differential healthcare and surveillance contact, and residual socioeconomic or behavioral confounding. None of them implicates cancer mechanistically, which is the point of a negative control.

The negative-control exposure sharpens that into a measurement. The skin-cancer-only group reaches the cancer question by the same self-report route as the cohort, carries little cancer mortality, and receives no cardiotoxic treatment, so whatever their mortality contrast against participants with no reported cancer shows is what the survivor contrast would show in the absence of a cancer-specific pathway. For cardiovascular death the two contrasts are almost the same: 0.84 (0.71–1.00) for survivors and 0.87 (0.65–1.18) for the skin-cancer-only group. The calibrated contrast, 0.96 (0.70–1.32), is what remains of the survivor comparison once the shared pathway is removed, and it is indistinguishable from no contrast at all.

Two readings follow, and the paper commits to the narrower one. The survivor cohort’s lower adjusted hazard of cardiovascular death relative to people reporting no cancer is not evidence of protection; it is what a prevalent-survivor sample looks like once selection into the sample, reporting, and surveillance are not separable from the exposure. The within-survivor comparisons of Section 3.3 and Section 3.7 are not exposed to this structure, since they never involve participants without cancer, and they are where the paper’s substantive claims sit. For the survivor-versus-no-cancer contrast itself, the honest statement is a bound rather than an estimate: a control exposure that should carry none of the cancer-specific signal carries essentially all of the observed contrast.

### 4.4. Unmeasured Treatment, Left Truncation, and the Cardiotoxicity Proxy

NHANES records no cancer treatment: not chemotherapy regimens, not anthracycline or trastuzumab exposure, not chest or mediastinal radiotherapy fields, and not androgen-deprivation therapy. These are the principal documented mechanisms of cardiovascular harm in cardio-oncology [1,5]. This is the largest unmeasured confounder in the study and it cannot be resolved with these data, which is why the site-level cardiotoxicity proxy of Section 3.7 is built, reported, and read as *suggestive* rather than confirmatory. Its result runs against the treatment hypothesis: survivors of high-expected-cardiotoxicity sites had a lower, not higher, subdistribution hazard of cardiovascular death (0.55, 95% CI: 0.33–0.91). It should not be read the other way either, as cardioprotection. The contrast compares cancer sites, not treatments, and the high- and low-expected groups differ in age, sex, stage mix, and baseline cardiovascular risk in ways the covariate set captures only partly. The E-value at the confidence bound, 1.44 (Table 8), means a modest confounder on both counts would be enough to move the interval across the null.

Left truncation is the other explanation this contrast invites, and it was tested rather than assumed. Survivors enter the cohort a median of eight years after diagnosis, so people who died of treatment-related cardiotoxicity soon after diagnosis are absent by construction, and the sites with the most early treatment-related mortality are the ones in the high-expected group. If that selection produced the inverse contrast, then moving to the time-since-diagnosis scale with delayed entry and restricting to survivors examined soon after diagnosis should pull it toward the null. Neither does. On the diagnosis time scale the contrast is 0.46 (0.27–0.77), further from the null than on the examination scale, and the restricted analysis is reported in Section 3.4. Left truncation is therefore not what produces the inverse direction here, which leaves site-mix confounding, the coarseness of a site-level proxy for an individual treatment, and a genuine inverse association among the explanations this design cannot separate.

### 4.5. Cause of Death, Self-Report, and What the Excess-Mortality Analysis Adds

Cancer diagnosis, site, and age at diagnosis are all self-reported NHANES interview items, with no registry confirmation of diagnosis, site, stage, or histology [9]. Cause of death carries two further problems. Attributing a death to cancer or to cardiovascular disease is error-prone in people with a cancer history, and the public-use Linked Mortality File substitutes synthetic values for the underlying cause or the follow-up time in some records while leaving vital status intact [16]. A third problem is specific to this release: seven of the ten leading-cause categories carry no deaths in the last two cycles, which is why the cardiovascular outcome here is deaths from diseases of the heart, coded alike in every cycle, with the wider definition reported only where it is identifiable (Section 3.2).

The excess-mortality analysis is the check that does not depend on any of this. Net survival at 10 years, 0.897 (95% CI: 0.869–0.924), implies excess mortality of 0.103 against expected rates estimated in the examined adults with no reported cancer, and the recorded cancer-death cumulative incidence at the same horizon is 0.115 (0.102–0.127). Agreement to about one percentage point does not prove that individual causes of death are correctly assigned. What it does is bound the aggregate: if cause-of-death error or synthetic substitution were distorting the cancer-death results materially, the two estimates would not line up this closely, because only one of them reads the cause from the death record. Site-level misclassification of self-reported diagnosis remains, and it compounds the ecological imprecision of the cardiotoxicity proxy (Section 4.4): survivors assigned to the wrong cardiotoxicity group bias that contrast toward the null relative to a treatment-confirmed one.

### 4.6. Comparison with Prior Work

This study’s finding that cardiovascular disease competes meaningfully with cancer as a cause of death among survivors is directionally consistent with registry-based evidence, including the English national cohort in which cardiovascular mortality overtook the primary cancer within a few years of diagnosis among older survivors [2], though that cohort’s registry-confirmed diagnoses and larger sample (104,028 adults) come at the cost of the population-representativeness and harmonized examination-based covariate panel this NHANES-based analysis offers instead. Among NHANES-specific cancer-survivor mortality studies, Zhao et al.’s nutrition-index analysis of a similarly sized cohort (4366 survivors, NHANES 1999–2018) [8] did not model cardiovascular and cancer death as formally competing risks of each other and did not include a negative-control check. United States registry analyses of cardiovascular mortality after cancer report raised standardized mortality for most sites and a large share of non-cancer deaths from heart disease [3,4]; those cohorts carry registry-confirmed diagnoses and treatment detail that NHANES lacks, and they do not carry the harmonized examination-based risk-factor panel used here. The decomposition of the comorbidity index into its cardiovascular components, the negative-control exposure, and the excess-mortality comparison are, to our knowledge, new to this NHANES cancer-survivor literature.

### 4.7. Strengths and Limitations

This study’s principal strength is that each of the three ways a treatment-blind population survey can mislead is measured rather than acknowledged. Frailty and surveillance confounding are measured with a negative-control outcome and a negative-control exposure, and the survivor contrast is calibrated against the latter. Unmeasured treatment is bounded with an E-value on an explicitly ecological site-level proxy. Left truncation is addressed by refitting on the time-since-diagnosis scale with delayed entry, and cause-of-death misattribution by an excess-mortality analysis that uses no cause-of-death information. The dual parsimonious and fully adjusted Fine–Gray specification, triangulated against a gradient-boosted cause-specific ensemble with SHAP attribution, gives two methodologically distinct views that agree on the headline pattern. Every reported confidence interval accounts for the NHANES complex-survey design, including the machine-learning discrimination metric, and every stratum’s cell size, including the smaller subset eligible for a SHAP decomposition, is reported beside its estimate.

Several limitations bound what this study can support, beyond the control and treatment-confounding discussions of Section 4.3 and Section 4.4. NHANES cancer diagnosis, site, and age at diagnosis are self-reported with no registry confirmation (Section 4.5). Cause of death comes from a public-use file in which NCHS substitutes synthetic values for the underlying cause or the follow-up time in some records, and in which seven of the ten leading-cause categories are unavailable in the last two cycles, which is why the cardiovascular outcome is restricted to diseases of the heart (Section 2.4). The fully adjusted models exclude both cycles in which *C*-reactive protein was not measured (2011–2014), so they describe eight of the ten pooled cycles; dropping that one covariate restores all ten and leaves the headline association in place (Section 3.11). Listwise deletion retained 2225 of 3826 survivors (58.2%) for the covariate-complete models and dropped 33.2% of the cohort on the compact imputation set. The imputation comparison (Table S12) reversed only coefficients close to zero in both fits, but MICE assumes data missing at random, which cannot be tested and may fail if sicker survivors were less likely to complete every laboratory component of the examination.

The two control analyses are themselves imprecise. Accidental death is a rare outcome, 199 deaths in the covariate-complete control population, so both control estimates have intervals that include the null, and the calibrated cardiovascular contrast is correspondingly wide. What the controls establish is the direction and approximate size of the shared pathway, not its exact magnitude. The delayed-entry models rest on the assumption that entry time is independent of the cause-specific hazard given the covariates, and their inverse-probability weights reach 12 at the extreme, so late risk sets are carried by few people. Expected mortality in the excess-mortality analysis is modeled in the same survey rather than taken from a national life table, which keeps the comparison population comparable but makes the expected rates conditional on the covariates in that model; the intervals treat them as known. The cardiotoxicity site-level proxy (Section 2.6, Section 3.7 and Section 4.4) is ecological by design and cannot observe individual treatment, and an E-value of 1.44 at the confidence bound means a modest confounder could remove its contrast. Prostate cancer is the weakest member of its high-cardiotoxicity group. The ensemble’s cause-specific hazard is not the Fine–Gray subdistribution hazard it is triangulated against (Section 2.10), so their agreement is directional corroboration rather than a formal equivalence test. Calibration and decision-curve analysis were restricted to subjects with a determinable 10-year status (Section 2.11), which excludes those censored or removed by a competing event earlier and is a documented simplification.

### 4.8. Future Work

Three extensions follow from these limitations. The first is external validation on a treatment-annotated cohort, such as a SEER-Medicare or comparable claims-linked resource carrying chemotherapy regimen, radiotherapy field, and androgen-deprivation-therapy administration codes, which would test the cardiotoxicity contrast and its E-value bound against observed individual treatment rather than a site-level substitute. The second is empirical calibration against a distribution of negative controls rather than the single control exposure used here [17]: a panel of exposures and outcomes with no plausible cancer pathway would give an estimated null distribution, and therefore a calibrated interval rather than a calibrated point contrast. The third is a registry-linked diagnosis date. Time since diagnosis is self-reported here, and it is both the time scale of the delayed-entry models and the variable that defines the recently diagnosed subset, so a confirmed date would sharpen the one analysis that addresses left truncation. Extending mortality follow-up past the 2019 release would accrue events for all three.

## 5. Conclusions

Among 3826 United States cancer survivors linked to cause-specific mortality through 2019, a higher comorbidity index was associated with a higher subdistribution hazard of death from diseases of the heart (hazard ratio 1.40, 95% CI: 1.24–1.59), and a direct decomposition shows that association is carried by the index’s five prior-cardiovascular-diagnosis components rather than by comorbidity burden in general. The estimate is unchanged when the models are refitted on the time-since-diagnosis scale with delayed entry (1.41, 1.22–1.64). Older age was associated with both causes of death, and female sex, never having smoked, and the number of reported cancer sites with cancer death. The survivor-versus-no-cancer comparison is a different matter: a negative-control outcome is raised in survivors, a negative-control exposure with no cancer-specific pathway reproduces the survivor contrast almost exactly, and what survives calibration against it is indistinguishable from no contrast. Excess mortality estimated without any cause-of-death information agrees with the recorded cancer deaths, so cause attribution is not what limits these data. Survivor mortality contrasts drawn from a treatment-blind population survey should be read as bounded by selection and surveillance, and within-survivor comparisons are where such a survey is informative.

## Figures and Tables

**Table 1:** Participant and clinical characteristics at baseline, stratified by cause-specific outcome: design-weighted median (interquartile range) for continuous variables and design-weighted per cent for binary and categorical variables.

Variable	Alive/Censored (*n* = 2410)	CVD Death (*n* = 288)	Cancer Death (*n* = 497)	Other-Cause Death (*n* = 631)	Overall (*n* = 3826)
Age (y)	60.0 (48.0, 70.0)	78.0 (71.0, 80.0)	70.0 (62.0, 79.0)	76.0 (68.0, 80.0)	65.0 (52.0, 75.0)
Female, %	66.3%	51.4%	46.8%	54.8%	62.0%
BMI (kg/m^2^)	28.2 (24.5, 33.1)	27.8 (24.7, 31.6)	26.9 (23.7, 31.1)	26.4 (23.2, 30.6)	27.8 (24.2, 32.5)
Systolic BP (mmHg)	123.3 (112.7, 136.0)	136.1 (121.6, 153.8)	127.1 (114.0, 144.7)	134.0 (118.0, 151.3)	125.3 (114.0, 140.0)
Diastolic BP (mmHg)	70.7 (64.0, 77.3)	64.0 (55.8, 73.3)	68.7 (60.0, 75.3)	66.1 (58.0, 74.0)	70.0 (62.0, 76.7)
Income-to-poverty ratio	3.4 (1.8, 5.0)	2.3 (1.2, 3.3)	2.4 (1.3, 4.4)	2.0 (1.2, 3.5)	3.1 (1.6, 5.0)
Comorbidity index	1.0 (0.0, 1.0)	2.0 (1.0, 3.0)	1.0 (0.0, 2.0)	1.0 (0.0, 2.0)	1.0 (0.0, 2.0)
Time since diagnosis (y)	9.0 (4.0, 17.0)	8.0 (2.0, 16.0)	6.0 (2.0, 12.3)	10.0 (4.0, 18.0)	8.0 (3.0, 16.0)
Number of cancer sites	1.0 (1.0, 1.0)	1.0 (1.0, 1.0)	1.0 (1.0, 2.0)	1.0 (1.0, 1.0)	1.0 (1.0, 1.0)
Hypertension, %	46.2%	70.0%	55.4%	63.5%	50.6%
Diabetes, %	14.2%	26.3%	16.1%	21.2%	15.9%
Alcohol, ≥12 drinks in any year, %	72.4%	52.8%	64.9%	59.4%	69.0%
Physically active, %	43.5%	19.5%	21.5%	17.2%	36.7%
Smoking: current, %	19.2%	11.7%	20.8%	14.3%	18.3%
Smoking: former, %	33.7%	46.7%	48.7%	47.3%	37.7%
Smoking: never, %	47.0%	41.6%	30.5%	38.3%	43.9%

**Table 2:** Laboratory measurements at baseline, stratified by cause-specific outcome: design-weighted median (interquartile range).

Variable	Alive/Censored (*n* = 2410)	CVD Death (*n* = 288)	Cancer Death (*n* = 497)	Other-Cause Death (*n* = 631)	Overall (*n* = 3826)
Total cholesterol (mg/dL)	196.0 (169.0, 226.0)	190.5 (158.0, 216.0)	193.0 (166.0, 223.3)	194.0 (168.0, 219.9)	195.0 (168.0, 224.0)
HDL cholesterol (mg/dL)	52.0 (42.8, 64.0)	48.0 (40.0, 59.0)	52.0 (42.0, 65.0)	53.2 (44.0, 66.0)	52.0 (42.0, 64.0)
HbA1c (%)	5.6 (5.3, 5.9)	5.8 (5.5, 6.3)	5.6 (5.3, 5.9)	5.6 (5.4, 6.0)	5.6 (5.3, 5.9)
*C*-reactive protein (mg/L)	2.0 (0.9, 4.7)	2.8 (1.3, 6.0)	3.4 (1.5, 7.0)	3.2 (1.3, 7.2)	2.3 (1.0, 5.5)
Hemoglobin (g/dL)	14.0 (13.2, 14.8)	13.7 (12.8, 14.7)	13.9 (12.8, 14.8)	13.8 (12.6, 14.8)	13.9 (13.1, 14.8)
Serum albumin (g/dL)	4.2 (4.0, 4.4)	4.1 (3.9, 4.4)	4.2 (3.9, 4.4)	4.1 (3.9, 4.3)	4.2 (4.0, 4.4)

**Table 3:** Cause-specific mortality outcome summary, NHANES 1999–2018 survivor cohort linked to the 2019 Linked Mortality File.

Outcome	*n*	% of Cohort
Linked to LMF (MORTSTAT non-missing)	3825	100.0%
Any death by 2019	1416	37.0%
Cardiovascular death (event = 1)	288	7.5%
Cancer death (event = 2)	497	13.0%
Other-cause death (event = 3)	631	16.5%
Alive/censored at 2019 (event = 0)	2410	63.0%
Cardiovascular death, diseases of the heart, cycles 1999–2014	270	of 2953 survivors
Cardiovascular death, heart or cerebrovascular, cycles 1999–2014	333	of 2953 survivors

**Table 4:** Headline covariates of the survey-weighted Fine–Gray subdistribution-hazard models for cardiovascular death and cancer death: the primary, fully adjusted (25-covariate) model with Holm–Bonferroni-adjusted *p*-values, and the parsimonious (ten-covariate) model fit on the same subjects. Design-aware jackknife 95% confidence intervals (238 jackknife units, 118 strata, 120 degrees of freedom; complete-case *n* = 2225).

Covariate	CVD Death HR (95% CI), Fully Adjusted	CVD *p*adj	CVD Death HR (95% CI), Parsimonious	Cancer-Death HR (95% CI), Fully Adjusted	Cancer *p*adj	Cancer-Death HR (95% CI), Parsimonious
Age (per year)	1.08 (1.06–1.10)	*p* < 0.001	1.10 (1.08–1.11)	1.03 (1.01–1.05)	*p* = 0.012	1.04 (1.02–1.05)
Female sex	0.74 (0.50–1.08)	*p =* 0.947	0.87 (0.63–1.19)	0.50 (0.34–0.74)	*p* = 0.005	0.64 (0.47–0.85)
Time since diagnosis (per year)	1.00 (0.99–1.01)	*p* = 1.000	1.00 (0.99–1.01)	0.99 (0.98–1.00)	*p* = 0.288	0.98 (0.97–1.00)
Hypertension	0.81 (0.51–1.28)	*p* = 1.000	0.82 (0.55–1.23)	1.17 (0.81–1.70)	*p* = 1.000	1.23 (0.87–1.74)
Diabetes	0.89 (0.52–1.54)	*p* = 1.000	0.86 (0.56–1.34)	1.06 (0.63–1.78)	*p* = 1.000	1.14 (0.74–1.78)
*C*-reactive protein (per mg/L)	1.00 (0.98–1.02)	*p* = 1.000	1.01 (0.99–1.03)	1.02 (1.00–1.04)	*p* = 0.204	1.02 (1.01–1.04)
BMI	0.99 (0.95–1.03)	*p* = 1.000	1.00 (0.96–1.03)	1.00 (0.96–1.03)	*p* = 1.000	0.99 (0.96–1.03)
Number of cancer sites	0.92 (0.59–1.45)	*p* = 1.000	0.87 (0.57–1.32)	2.18 (1.54–3.08)	*p* < 0.001	2.15 (1.58–2.92)
Comorbidity index	1.40 (1.24–1.59)	*p* < 0.001	1.43 (1.28–1.59)	0.89 (0.80–1.00)	*p* = 0.288	0.92 (0.82–1.03)
Smoking: never (ref. current)	0.74 (0.44–1.26)	*p* = 1.000	0.76 (0.44–1.31)	0.48 (0.32–0.72)	*p* = 0.004	0.49 (0.32–0.75)

**Table 5:** Headline covariates and the cardiotoxicity contrast on the time-since-diagnosis scale with delayed entry at the examination, and in the subset examined within five years of diagnosis.

Covariate	CVD Death HR (95% CI), Delayed Entry	CVD Death HR (95% CI), Examined Within 5 Years	Cancer-Death HR (95% CI), Delayed Entry	Cancer-Death HR (95% CI), Examined Within 5 Years
Age (per year)	1.07 (1.05–1.09)	1.07 (1.05–1.10)	1.03 (1.01–1.05)	1.03 (1.00–1.06)
Female sex	0.97 (0.64–1.47)	0.71 (0.38–1.33)	0.62 (0.42–0.90)	0.59 (0.29–1.18)
Hypertension	0.83 (0.52–1.33)	0.48 (0.23–0.99)	1.23 (0.85–1.80)	0.98 (0.54–1.75)
Diabetes	1.09 (0.63–1.89)	0.96 (0.30–3.04)	1.23 (0.75–2.04)	2.05 (1.02–4.11)
*C*-reactive protein (per mg/L)	0.99 (0.97–1.02)	1.00 (0.96–1.04)	1.02 (1.00–1.04)	1.02 (1.00–1.05)
BMI	0.99 (0.95–1.04)	0.99 (0.91–1.07)	1.00 (0.97–1.04)	1.02 (0.97–1.08)
Number of cancer sites	1.16 (0.73–1.83)	1.06 (0.40–2.80)	2.64 (1.85–3.78)	2.99 (1.53–5.84)
Comorbidity index	1.41 (1.22–1.64)	1.66 (1.23–2.24)	0.86 (0.76–0.98)	0.71 (0.55–0.91)
Smoking: never (ref. current)	0.75 (0.44–1.28)	0.82 (0.45–1.51)	0.51 (0.33–0.78)	0.48 (0.26–0.91)
High- vs. low-expected cardiotoxicity	0.46 (0.27–0.77)	0.66 (0.31–1.42)	—	—

**Table 6:** Observed, expected, and net survival among survivors at 5 and 10 years, against expected mortality modeled internally in the examined adults with no reported cancer.

Quantity	5 Years	10 Years
Observed all-cause survival	0.849 (0.835–0.863)	0.701 (0.682–0.720)
Expected survival, no reported cancer	0.896 (0.892–0.901)	0.771 (0.761–0.780)
Relative survival (observed/expected)	0.947 (0.933–0.961)	0.909 (0.889–0.930)
Net survival (Pohar–Perme)	0.945 (0.930–0.960)	0.897 (0.869–0.924)
Recorded cancer-death cumulative incidence	0.068 (0.059–0.077)	0.115 (0.102–0.127)

**Table 7:** Negative-control outcome, negative-control exposure, and the calibrated survivor contrast, adjusted for the full covariate set.

Outcome	Contrast	HR (95% CI)	Analyzed *n*	Events
Cardiovascular death	Survivor cohort vs. no reported cancer	0.84 (0.71–1.00)	42,728	1700
Cardiovascular death	Skin cancer only vs. no reported cancer	0.87 (0.65–1.18)	42,728	1700
Cardiovascular death	Calibrated: survivor minus skin cancer only	0.96 (0.70–1.32)	42,728	1700
Accidental death	Survivor cohort vs. no reported cancer	1.78 (0.89–3.54)	34,141	199
Accidental death	Skin cancer only vs. no reported cancer	2.01 (0.87–4.66)	34,141	199
Accidental death	Calibrated: survivor minus skin cancer only	0.88 (0.33–2.33)	34,141	199

**Table 8:** Cardiotoxicity site-level contrast (high- versus low-expected cardiotoxicity), the time-since-diagnosis interaction probe, and the VanderWeele–Ding E-value, cardiovascular death, and design-aware Fine–Gray model (complete-case *n* = 1403; 228 jackknife units, 113 strata, 115 degrees of freedom).

Model Term	Value
High- vs. low-expected cardiotoxicity	0.55 (0.33–0.91)
High- vs. low-expected cardiotoxicity, interaction model	0.57 (0.28–1.14)
Cardiotoxicity × time-since-diagnosis interaction	1.00 (0.96–1.03)
E-value at the point estimate	3.04
E-value at the confidence bound closer to the null	1.44

## Data Availability

The data presented in this study are available in the NHANES public-use files at https://wwwn.cdc.gov/nchs/nhanes/ (accessed on 16 October 2026) and the NHANES Linked Mortality File at https://ftp.cdc.gov/pub/Health_Statistics/NCHS/datalinkage/linked_mortality/ (accessed on 27 October 2026); both are released in the public domain by the U.S. federal government, without an additional third-party license restriction. These data were derived from the following NHANES components, pooled across the ten two-year cycles 1999–2000 through 2017–2018: Demographics (DEMO), Medical Conditions (MCQ), Body Measures (BMX), Blood Pressure (BPX), Standard Biochemistry Profile (BIOPRO), Complete Blood Count (CBC), C-Reactive Protein (cycle-appropriate conventional or high-sensitivity assay files), Plasma Fasting Glucose (GLU), Glycohemoglobin (GHB), Blood Pressure & Cholesterol Questionnaire (BPQ), Diabetes Questionnaire (DIQ), Smoking Questionnaire (SMQ), Total Cholesterol (TCHOL), HDL Cholesterol (HDL), Triglycerides (TRIGLY), Hospital Utilization and Access to Care (HUQ), Alcohol Use (ALQ), Physical Activity (PAQ), and the 2019 public-use NHANES Linked Mortality File (LMF). The full analysis pipeline (cohort construction, outcome ascertainment, survey-weighted Fine–Gray and gradient-boosted competing-risks models, SHAP analysis, the cardiotoxicity and negative-control probes, multiple-imputation sensitivity analysis, and table generation) is provided in full, as a formatted source-code listing, in Supplementary File S1 accompanying this article, released under the MIT license. The NHANES public-use files and the Linked Mortality File analyzed here are openly available and have also been used by this research group to address other, unrelated research questions; no participant-level result, table, or figure is shared between this paper and any other analysis.

## Supplementary Materials

The following supporting information can be downloaded at: https://www.mdpi.com/article/10.3390/cancers18213544/s1. Supplementary File S1: Reproducible Analysis Code, a formatted listing of the complete analysis pipeline (NHANES ingestion and harmonization, cohort and outcome construction, covariate engineering, Fine–Gray and gradient-boosted competing-risks model fitting, the delayed-entry and relative-survival analyses, the negative-control models, design-aware jackknife confidence intervals, SHAP analysis, and table generation), released under the MIT license; Supplementary File S2: Supplementary Tables, comprising Table S1: Sequential subsample inventory for the NHANES 1999–2018 cancer-survivor cohort; Table S2: NCHS leading-cause-of-death recode by survey cycle among decedents of the survivor cohort; Table S3: Effect of the cardiovascular-death definition: event counts under each definition, and the headline covariates of the fully adjusted model under both definitions on the eight cycles in which cerebrovascular death is coded separately; Table S4: Negative-control outcome and negative-control exposure in the examined adult population: both exposure indicators entered together with participants reporting no cancer as the reference, under two adjustment sets; Table S5: Target-leakage pre-flight audit: every covariate screened for an algebraic or definitional relationship to the outcome, with the decision and its reason; Table S6: Fully adjusted survey-weighted Fine–Gray subdistribution-hazard model for cardiovascular death and cancer death (32 coefficients; race/ethnicity and education entered as indicator variables), with design-aware jackknife 95% confidence intervals (238 jackknife units, 118 strata, 120 degrees of freedom; complete-case *n* = 2225); Table S7: Delayed-entry analysis on the time-since-diagnosis scale: subsets analyzed, and the distribution of entry times, exit times, and inverse-probability weights; Table S8: Observed, expected, and net survival among survivors against expected mortality modeled internally in the examined adults with no reported cancer (*n* = 43,025), survivor cohort *n* = 3473; Table S9: Calibration by risk decile and decision-curve net benefit for the 10-year cardiovascular-death score; Table S10: Ten globally most influential features by mean absolute SHAP value in the cause-specific gradient-boosted ensembles (complete-case *n* = 2225); Table S11: Stratum sample sizes for cancer site and time since diagnosis: full stratum *n* against the smaller subset eligible for SHAP decomposition (minimum 50 covariate-complete cases); Table S12: Complete-case against multiple-imputation (MICE, ten imputations, Rubin’s rules) cause-specific Cox log-hazard ratios for cardiovascular death and cancer death, 11-covariate sensitivity set; Table S13: Headline covariates with and without C-reactive protein; Supplementary File S3: Extended Methods, the implementation detail of each analysis.
