# Supplementary material for: Negative-Control Calibration and E-Value Bounding of Cancer-Survivor Mortality Contrasts: A Design-Aware Competing-Risks Analysis
Source: Cancers (Basel). 2026 Nov 4;18(21):3544. doi: 10.3390/cancers18213544 (PMC13762343; doi:10.3390/cancers18213544)
Supplement: Supplementary file 1 [file cancers-18-03544-s001.zip › Supplementary File S1.pdf]

Negative-Control and E-Value Calibration of Cancer-Survivor Mortality Contrasts: A Design-Aware Competing-Risks Analysis

Supplementary File S1: Reproducible Analysis Code

This document contains the complete, formatted source code accompanying the article above, organized by file. Each listing is reproduced verbatim from the reproducible analysis pipeline; line numbers are added for reference only.

Licence. This code is released under the MIT licence, as stated in the article's Data Availability Statement.

Contents

1 `src/config.py` 2

2 `src/nhanes_files.py` 4

3 `src/nhanes_loader.py` 5

4 `src/lmf_loader.py` 7

5 `src/cancer_items.py` 8

6 `src/cohort.py` 10

7 `src/outcomes.py` 13

8 `src/covariates.py` 15

9 `src/cardiotoxicity.py` 18

10 `src/stratify.py` 19

11 `src/survey.py` 20

12 `src/table1.py` 24

13 `src/audits.py` 26

14 `src/finegray.py` 28

15 `src/finegray_truncated.py` 33

16 `src/left_truncation.py` 37

17 `src/negative_control.py` 42

18 `src/relative_survival.py` 46

19 `src/gbm_competing_risk.py` 51

20 `src/calibration_dca.py` 53

21 `src/shap_analysis.py` 55

22 `src/baseline_models.py` 56

23 `src/mi_sensitivity.py` 60

24 `src/crp_sensitivity.py` 62

25 src/design_based_intervals.py **63**

26 src/condition_answer_codes.py **66**

27 src/fmt.py **67**

28 src/viz.py **69**

29 src/figures.py **71**

30 src/make_figures.py **77**

31 src/pipeline.py **78**

32 scripts/run_all.py **89**

1 src/config.py

```python
from __future__ import annotations

import logging
import os
from pathlib import Path

RANDOM_SEED: int = int(os.environ.get("RANDOM_SEED", "42"))

ROOT = Path(__file__).resolve().parents[1]
ARTICLES = ROOT.parent
DATA_RAW = ROOT / "data" / "raw"
DATA_INTERIM = ROOT / "data" / "interim"
DATA_PROCESSED = ROOT / "data" / "processed"
RESULTS = ROOT / "results"
RESULTS_FIGURES = RESULTS / "figures"
RESULTS_TABLES = RESULTS / "tables"
RESULTS_MODELS = RESULTS / "models"
RESULTS_METRICS = RESULTS / "metrics"
ARTICLE_DIR = ROOT / "article"

SHARED_RAW = Path(
    os.environ.get("NHANES_RAW_DIR") or (ARTICLES / "_nhanes_raw")
)
SHARED_CACHE = ARTICLES / "_nhanes_cache"

for _p in (
    DATA_INTERIM,
    DATA_PROCESSED,
    RESULTS_FIGURES,
    RESULTS_TABLES,
    RESULTS_MODELS,
    RESULTS_METRICS,
    ARTICLE_DIR,
):
    _p.mkdir(parents=True, exist_ok=True)

NHANES_CYCLES: dict[str, tuple[str, str]] = {
    "1999-2000": ("1999", ""),
    "2001-2002": ("2001", "_B"),
    "2003-2004": ("2003", "_C"),
    "2005-2006": ("2005", "_D"),
    "2007-2008": ("2007", "_E"),
    "2009-2010": ("2009", "_F"),
    "2011-2012": ("2011", "_G"),
    "2013-2014": ("2013", "_H"),
    "2015-2016": ("2015", "_I"),
    "2017-2018": ("2017", "_J"),
}
N_CYCLES = 10

LEADING_CAUSE_DETAILED_CYCLES: list[str] = [
    "1999-2000",
    "2001-2002",
    "2003-2004",
    "2005-2006",
    "2007-2008",
    "2009-2010",
    "2011-2012",
    "2013-2014",
]

RAW_COMPONENT_FILES: dict[str, list[str]] = {
    "DEMO": ["DEMO"],
    "MCQ": ["MCQ"],
    "BMX": ["BMX"],
    "BPX": ["BPX"],
    "BIOPRO": ["BIOPRO", "LAB18", "L40"],
    "CBC": ["CBC", "LAB25", "L25"],
    "ALB_CR": ["ALB_CR", "L16"],
    "CRP": ["LAB11", "L11", "L11_2", "CRP", "HSCRP"],
    "GLU": ["GLU", "LAB10AM", "L10AM"],
    "GHB": ["GHB", "LAB10", "L10"],
    "BPQ": ["BPQ"],
    "DIQ": ["DIQ"],
```

```
    "KIQ_U": ["KIQ_U", "KIQ"],
    "SMQ": ["SMQ"],
}

SHARED_COMPONENTS = ["TCHOL", "HDL", "TRIGLY", "HUQ", "ALQ", "PAQ"]

NEEDED_COLUMNS: dict[str, list[str]] = {
    "DEMO": [
        "RIDAGEYR",
        "RIAGENDR",
        "RIDRETH1",
        "DMDEDUC2",
        "INDFMPIR",
        "SDMVPSU",
        "SDMVSTRA",
        "WTMEC2YR",
        "WTINT2YR",
        "SDDSRVYR",
    ],
    "MCQ": [
        "MCQ220",
        "MCQ230A",
        "MCQ230B",
        "MCQ230C",
        "MCQ230D",
        "MCD240A",
        "MCD240B",
        "MCD240C",
        "MCQ160B",
        "MCQ160C",
        "MCQ160D",
        "MCQ160E",
        "MCQ160F",
    ]
    + [
        f"MCQ240{L}"
        for L in list("ABCDEFGHIJKLMNOPQRSTUVWXYZ") + ["AA", "BB", "CC", "DD"]
    ],
    "BMX": ["BMXBMI"],
    "BPX": [
        "BPXSY1",
        "BPXSY2",
        "BPXSY3",
        "BPXSY4",
        "BPXDI1",
        "BPXDI2",
        "BPXDI3",
        "BPXDI4",
    ],
    "BIOPRO": ["LBXSAL"],
    "CBC": ["LBXHGB", "LBDLYMNO", "LBDNENO"],
    "ALB_CR": [],
    "CRP": ["LBXCRP", "LB2CRP", "LBXHSCRP"],
    "GLU": ["LBXGLU"],
    "GHB": ["LBXGH"],
    "BPQ": ["BPQ020"],
    "DIQ": ["DIQ010"],
    "KIQ_U": [],
    "SMQ": ["SMQ020", "SMQ040"],
    "TCHOL": ["LBXTC"],
    "HDL": ["LBDHDL", "LBDHDD", "LBXHDD"],
    "TRIGLY": ["LBXTR"],
    "HUQ": ["HUQ010", "HUQ050", "HUQ051"],
    "ALQ": ["ALQ100", "ALD100", "ALQ101", "ALQ111"],
    "PAQ": ["PAQ180", "PAQ650", "PAQ665"],
}

LMF_PER_CYCLE = {
    "1999-2000": "NHANES_1999_2000_MORT_2019_PUBLIC.dat",
    "2001-2002": "NHANES_2001_2002_MORT_2019_PUBLIC.dat",
    "2003-2004": "NHANES_2003_2004_MORT_2019_PUBLIC.dat",
    "2005-2006": "NHANES_2005_2006_MORT_2019_PUBLIC.dat",
    "2007-2008": "NHANES_2007_2008_MORT_2019_PUBLIC.dat",
    "2009-2010": "NHANES_2009_2010_MORT_2019_PUBLIC.dat",
    "2011-2012": "NHANES_2011_2012_MORT_2019_PUBLIC.dat",
    "2013-2014": "NHANES_2013_2014_MORT_2019_PUBLIC.dat",
```

```python
    "2015-2016": "NHANES_2015_2016_MORT_2019_PUBLIC.dat",
    "2017-2018": "NHANES_2017_2018_MORT_2019_PUBLIC.dat",
}

FOLLOWUP_YEARS_MAX: float = 20.0

N_BOOTSTRAP = 1000
N_JACKKNIFE_MAX_UNITS = None
SHAP_MIN_STRATUM_N = 50

COLORS = {
    "blue": "#0072B2",
    "orange": "#E69F00",
    "green": "#009E73",
    "pink": "#CC79A7",
    "red": "#D55E00",
    "sky": "#56B4E9",
    "yellow": "#F0E442",
    "grey": "#999999",
}

CANDIDATE_FEATURES: list[str] = [
    "age",
    "sex",
    "race_eth",
    "education",
    "pir",
    "bmi",
    "sbp",
    "dbp",
    "total_chol",
    "hdl",
    "triglycerides",
    "hba1c",
    "fasting_glucose",
    "crp",
    "nlr",
    "hemoglobin",
    "serum_albumin",
    "hypertension",
    "diabetes",
    "smoking_status",
    "alcohol_use",
    "physical_activity_met",
    "healthcare_visits_12mo",
    "functional_limitation",
    "time_since_diagnosis",
    "age_at_diagnosis",
    "n_cancer_sites",
]


def configure_logging(level: int = logging.INFO) -> None:
    logging.basicConfig(
        level=level,
        format="%(asctime)s  %(levelname)-7s  %(name)s  %(message)s",
        datefmt="%H:%M:%S",
        force=True,
    )
```

2 src/nhanes_files.py

```python
from __future__ import annotations

import os
from pathlib import Path

ARTICLES = Path(__file__).resolve().parents[2]
CACHE = ARTICLES / "_nhanes_cache"
SHARED_RAW = Path(
    os.environ.get("NHANES_RAW_DIR") or (ARTICLES / "_nhanes_raw")
)
CACHE.mkdir(parents=True, exist_ok=True)
```

```
CYCLES: dict[str, tuple[str, str]] = {
    "1999-2000": ("1999", ""),
    "2001-2002": ("2001", "_B"),
    "2003-2004": ("2003", "_C"),
    "2005-2006": ("2005", "_D"),
    "2007-2008": ("2007", "_E"),
    "2009-2010": ("2009", "_F"),
    "2011-2012": ("2011", "_G"),
    "2013-2014": ("2013", "_H"),
    "2015-2016": ("2015", "_I"),
    "2017-2018": ("2017", "_J"),
}
COMPONENTS: dict[str, list[str]] = {
    "DR1TOT": ["DR1TOT", "DRXTOT"],
    "DR2TOT": ["DR2TOT"],
    "DSQTOT": ["DSQTOT", "DSQ1", "DSQ2"],
    "DBQ": ["DBQ"],
    "ALQ": ["ALQ"],
    "PAQ": ["PAQ"],
    "TCHOL": ["TCHOL", "L13", "LAB13"],
    "HDL": ["HDL", "L13", "LAB13"],
    "TRIGLY": ["TRIGLY", "L13AM", "LAB13AM"],
    "HUQ": ["HUQ"],
    "IMQ": ["IMQ"],
    "PFQ": ["PFQ"],
    "SLQ": ["SLQ"],
    "VIC": ["VIC", "L06VIT", "VITAEC", "L06"],
}
MIN_BYTES = 1024


def local_path(component: str, cycle: str, stem: str) -> Path:
    return (
        CACHE
        / f"{component}_{cycle.replace('-', '')}_{stem}{CYCLES[cycle][1]}.xpt"
    )


def resolve(component: str, cycle: str) -> Path | None:
    suffix = CYCLES[cycle][1]
    tag = cycle.replace("-", "")
    for stem in COMPONENTS.get(component, [component]):
        cached = local_path(component, cycle, stem)
        if cached.exists() and cached.stat().st_size > MIN_BYTES:
            return cached
        legacy = SHARED_RAW / f"{component}_{tag}_{stem}{suffix}.xpt"
        if legacy.exists() and legacy.stat().st_size > MIN_BYTES:
            return legacy
    return None
```

3 src/nhanes_loader.py

```
from __future__ import annotations

from pathlib import Path

import pandas as pd
import pyreadstat

from src import nhanes_files as _shared

from .config import (
    DATA_INTERIM,
    NEEDED_COLUMNS,
    NHANES_CYCLES,
    RAW_COMPONENT_FILES,
    SHARED_COMPONENTS,
    SHARED_RAW,
)


def resolve_component(component: str, cycle: str) -> Path | None:
    if component in RAW_COMPONENT_FILES:
```

```python
        suffix = NHANES_CYCLES[cycle][1]
        tag = cycle.replace("-", "")
        for stem in RAW_COMPONENT_FILES[component]:
            fname = f"{stem}{suffix}.xpt"
            candidate = SHARED_RAW / f"{component}_{tag}_{fname}"
            if candidate.exists() and candidate.stat().st_size > 1024:
                return candidate
        return None
    if component in SHARED_COMPONENTS:
        return _shared.resolve(component, cycle)
    raise KeyError(f"{component!r} is not a known CAN-paper component")


def read_xpt(path: Path) -> pd.DataFrame:
    try:
        df, _ = pyreadstat.read_xport(str(path))
    except UnicodeDecodeError:
        df, _ = pyreadstat.read_xport(str(path), encoding="latin1")
    df.columns = [c.upper() for c in df.columns]
    return df


def pool_component(component: str, cache: bool = True) -> pd.DataFrame:
    cache_path = DATA_INTERIM / f"{component}.parquet"
    if cache and cache_path.exists():
        return pd.read_parquet(cache_path)
    keep = NEEDED_COLUMNS.get(component)
    frames: list[pd.DataFrame] = []
    for cycle in NHANES_CYCLES:
        path = resolve_component(component, cycle)
        if path is None:
            continue
        df = read_xpt(path)
        if keep is not None:
            present = [c for c in keep if c in df.columns]
            df = df[["SEQN"] + present].copy()
        df["CYCLE"] = cycle
        frames.append(df)
    if not frames:
        return pd.DataFrame()
    pooled = pd.concat(frames, axis=0, ignore_index=True, sort=False)
    pooled.to_parquet(cache_path, index=False)
    return pooled


def component_coverage() -> pd.DataFrame:
    all_components = list(RAW_COMPONENT_FILES) + list(SHARED_COMPONENTS)
    rows = []
    for component in all_components:
        got = [
            cycle
            for cycle in NHANES_CYCLES
            if resolve_component(component, cycle) is not None
        ]
        rows.append(
            {
                "component": component,
                "n_cycles": len(got),
                "cycles": ", ".join(got),
                "missing": ", ".join(c for c in NHANES_CYCLES if c not in got),
            }
        )
    return pd.DataFrame(rows)


def _coalesce_join(base: pd.DataFrame, df: pd.DataFrame) -> pd.DataFrame:
    df = df.drop(
        columns=[c for c in df.columns if c == "CYCLE"], errors="ignore"
    )
    df = df[~df.index.duplicated(keep="first")]
    overlap = base.columns.intersection(df.columns)
    for col in overlap:
        base[col] = base[col].where(base[col].notna(), df[col])
    df = df.drop(columns=overlap, errors="ignore")
    return base.join(df, how="left")

```

```python

def base_population() -> pd.DataFrame:
    demo = pool_component("DEMO").set_index("SEQN")
    mcq = pool_component("MCQ")
    mcq_idx = mcq.set_index("SEQN")
    merged = _coalesce_join(demo, mcq_idx)
    return merged.reset_index()


def attach_covariates(base: pd.DataFrame) -> pd.DataFrame:
    covariate_components = [
        c
        for c in list(RAW_COMPONENT_FILES) + list(SHARED_COMPONENTS)
        if c not in ("DEMO", "MCQ") and NEEDED_COLUMNS.get(c) != []
    ]
    id_col = "seqn" if "seqn" in base.columns else "SEQN"
    out = base.set_index(id_col)
    out.index.name = "SEQN"
    for component in covariate_components:
        df = pool_component(component)
        if df.empty:
            continue
        df = df.set_index("SEQN")
        df = df.reindex(out.index.intersection(df.index))
        out = _coalesce_join(out, df)
    out = out.reset_index()
    out = out.rename(columns={"SEQN": id_col})
    return out
```

4 src/lmf_loader.py

```python
from __future__ import annotations

from pathlib import Path

import pandas as pd

from .config import DATA_INTERIM, LMF_PER_CYCLE, SHARED_RAW

LMF_LAYOUT = [
    ("SEQN", 0, 6, int),
    ("ELIGSTAT", 14, 15, int),
    ("MORTSTAT", 15, 16, "Int8"),
    ("UCOD_LEADING", 16, 19, str),
    ("DIABETES", 19, 20, "Int8"),
    ("HYPERTEN", 20, 21, "Int8"),
    ("PERMTH_INT", 42, 45, "Int16"),
    ("PERMTH_EXM", 45, 48, "Int16"),
]


def _parse_dat(path: Path) -> list[dict]:
    rows = []
    with path.open("r", encoding="latin-1") as fh:
        for line in fh:
            if not line.strip():
                continue
            row = {}
            for name, start, end, dtype in LMF_LAYOUT:
                raw = line[start:end].strip()
                if raw == "" or set(raw) == {"."}:
                    row[name] = pd.NA
                elif dtype is str:
                    row[name] = raw
                else:
                    try:
                        row[name] = int(raw)
                    except ValueError:
                        row[name] = pd.NA
            rows.append(row)
    return rows


```

```python
def load_lmf(cache: bool = True) -> pd.DataFrame:
    cache_path = DATA_INTERIM / "lmf.parquet"
    if cache and cache_path.exists():
        return derive_cause_flags(pd.read_parquet(cache_path))

    rows: list[dict] = []
    found_cycles: list[str] = []
    for cycle, fname in LMF_PER_CYCLE.items():
        path = SHARED_RAW / fname
        if not path.exists() or path.stat().st_size < 100:
            continue
        found_cycles.append(cycle)
        rows.extend(_parse_dat(path))
    if len(found_cycles) != 10:
        missing = [c for c in LMF_PER_CYCLE if c not in found_cycles]
        raise RuntimeError(f"LMF files missing for cycles: {missing}")

    df = pd.DataFrame(rows)
    df = df[df["ELIGSTAT"] == 1].copy()
    df = derive_cause_flags(df)
    df.to_parquet(cache_path, index=False)
    return df


def derive_cause_flags(df: pd.DataFrame) -> pd.DataFrame:
    out = df.copy()
    ucod = out["UCOD_LEADING"].astype(str).str.strip()
    out["cv_death"] = ucod.isin({"001", "1"})
    out["cv_death_broad"] = ucod.isin({"001", "005", "1", "5"})
    out["cancer_death"] = ucod.isin({"002", "2"})
    out["followup_months"] = pd.to_numeric(
        out["PERMTH_EXM"], errors="coerce"
    ).fillna(pd.to_numeric(out["PERMTH_INT"], errors="coerce"))
    return out
```

5 src/cancer_items.py

```python
from __future__ import annotations

import pandas as pd

SITE_CODES: dict[int, tuple[str, str]] = {
    10: ("Bladder", "Genitourinary (other)"),
    11: ("Blood", "Hematologic"),
    12: ("Bone", "Other/rare"),
    13: ("Brain", "Other/rare"),
    14: ("Breast", "Breast"),
    15: ("Cervix", "Gynecologic"),
    16: ("Colon", "Colorectal"),
    17: ("Esophagus", "Digestive (other)"),
    18: ("Gallbladder", "Digestive (other)"),
    19: ("Kidney", "Genitourinary (other)"),
    20: ("Larynx", "Respiratory (other)"),
    21: ("Leukemia", "Hematologic"),
    22: ("Liver", "Digestive (other)"),
    23: ("Lung", "Respiratory (other)"),
    24: ("Lymphoma", "Hematologic"),
    25: ("Melanoma", "Skin (melanoma)"),
    26: ("Mouth/tongue/lip", "Other/rare"),
    27: ("Nervous system", "Other/rare"),
    28: ("Ovary", "Gynecologic"),
    29: ("Pancreas", "Digestive (other)"),
    30: ("Prostate", "Prostate"),
    31: ("Rectum", "Colorectal"),
    32: ("Skin, non-melanoma", "Skin (non-melanoma/unknown)"),
    33: ("Skin, unknown type", "Skin (non-melanoma/unknown)"),
    34: ("Soft tissue", "Other/rare"),
    35: ("Stomach", "Digestive (other)"),
    36: ("Testis", "Genitourinary (other)"),
    37: ("Thyroid", "Other/rare"),
    38: ("Uterus", "Gynecologic"),
    39: ("Other", "Other/rare"),
}
```

```python

LETTER_TO_CODE: dict[str, int] = {
    "A": 10,
    "B": 11,
    "C": 12,
    "D": 13,
    "E": 14,
    "F": 15,
    "G": 16,
    "H": 17,
    "I": 18,
    "J": 19,
    "K": 20,
    "L": 21,
    "M": 22,
    "N": 23,
    "O": 24,
    "P": 25,
    "Q": 26,
    "R": 27,
    "S": 28,
    "T": 29,
    "U": 30,
    "V": 31,
    "W": 32,
    "X": 33,
    "Y": 34,
    "Z": 35,
    "AA": 36,
    "BB": 37,
    "CC": 38,
    "DD": 39,
}

NON_MELANOMA_SKIN_GROUP = "Skin (non-melanoma/unknown)"

_MIN_AGE, _MAX_AGE = 0, 120


def _clean_age(s: pd.Series) -> pd.Series:
    s = pd.to_numeric(s, errors="coerce")
    return s.where((s >= _MIN_AGE) & (s <= _MAX_AGE))


def _legacy_long(mcq: pd.DataFrame) -> pd.DataFrame:
    rows = []
    for letter, code in LETTER_TO_CODE.items():
        col = f"MCQ240{letter}"
        if col not in mcq.columns:
            continue
        raw = pd.to_numeric(mcq[col], errors="coerce")
        age = _clean_age(raw)
        hit = raw.notna()
        if not hit.any():
            continue
        name, group = SITE_CODES[code]
        rows.append(
            pd.DataFrame(
                {
                    "seqn": mcq.loc[hit, "SEQN"].to_numpy(),
                    "cycle": mcq.loc[hit, "CYCLE"].to_numpy(),
                    "site_code": code,
                    "site_name": name,
                    "site_group": group,
                    "age_at_diagnosis": age.loc[hit].to_numpy(),
                }
            )
        )
    if not rows:
        return pd.DataFrame(
            columns=[
                "seqn",
                "cycle",
                "site_code",
                "site_name",
                "site_group",
```

```
                "age_at_diagnosis",
            ]
        )
    return pd.concat(rows, ignore_index=True)


def _redesign_long(mcq: pd.DataFrame) -> pd.DataFrame:
    rows = []
    for site_col, age_col in (
        ("MCQ230A", "MCD240A"),
        ("MCQ230B", "MCD240B"),
        ("MCQ230C", "MCD240C"),
    ):
        if site_col not in mcq.columns or age_col not in mcq.columns:
            continue
        code = pd.to_numeric(mcq[site_col], errors="coerce")
        age = _clean_age(mcq[age_col])
        hit = code.isin(SITE_CODES.keys())
        if not hit.any():
            continue
        codes_hit = code.loc[hit].astype(int)
        names = codes_hit.map(lambda c: SITE_CODES[c][0])
        groups = codes_hit.map(lambda c: SITE_CODES[c][1])
        rows.append(
            pd.DataFrame(
                {
                    "seqn": mcq.loc[hit, "SEQN"].to_numpy(),
                    "cycle": mcq.loc[hit, "CYCLE"].to_numpy(),
                    "site_code": codes_hit.to_numpy(),
                    "site_name": names.to_numpy(),
                    "site_group": groups.to_numpy(),
                    "age_at_diagnosis": age.loc[hit].to_numpy(),
                }
            )
        )
    if not rows:
        return pd.DataFrame(
            columns=[
                "seqn",
                "cycle",
                "site_code",
                "site_name",
                "site_group",
                "age_at_diagnosis",
            ]
        )
    return pd.concat(rows, ignore_index=True)


def cancer_events_long(mcq: pd.DataFrame) -> pd.DataFrame:
    legacy = mcq[mcq["CYCLE"] != "2017-2018"]
    redesign = mcq[mcq["CYCLE"] == "2017-2018"]
    out = pd.concat(
        [_legacy_long(legacy), _redesign_long(redesign)], ignore_index=True
    )
    out["seqn"] = out["seqn"].astype(int)
    return out
```

6 src/cohort.py

```
from __future__ import annotations

import numpy as np
import pandas as pd

from .cancer_items import NON_MELANOMA_SKIN_GROUP, cancer_events_long
from .config import DATA_INTERIM, RESULTS_TABLES
from .nhanes_loader import attach_covariates, base_population, pool_component


def _primary_site(events: pd.DataFrame) -> pd.DataFrame:
    events = events.copy()
    events["_has_age"] = events["age_at_diagnosis"].notna()
```

```python
    events = events.sort_values(
        ["seqn", "_has_age", "age_at_diagnosis"], ascending=[True, False, True]
    )
    n_sites = events.groupby("seqn").size().rename("n_cancer_sites")
    any_melanoma_or_other = events.groupby("seqn")["site_group"].apply(
        lambda g: bool((g != NON_MELANOMA_SKIN_GROUP).any())
    )
    primary = events.groupby("seqn", as_index=True).first()[
        ["site_code", "site_name", "site_group", "age_at_diagnosis"]
    ]
    primary = primary.join(n_sites).join(
        any_melanoma_or_other.rename("has_non_skin_cancer")
    )
    return primary.reset_index()


def build_cohort() -> tuple[pd.DataFrame, pd.DataFrame]:
    inventory: list[tuple[str, int, str]] = []

    merged = base_population()
    inventory.append(
        (
            "NHANES 1999-2018, DEMO merged with MCQ on SEQN",
            merged["SEQN"].nunique(),
            "full population, both sexes, all ages",
        )
    )

    merged["RIDAGEYR"] = pd.to_numeric(merged["RIDAGEYR"], errors="coerce")
    adults = merged[merged["RIDAGEYR"] >= 20].copy()
    inventory.append(
        (
            "Adults >=20 y (all cycles)",
            len(adults),
            "RIDAGEYR >= 20, interview-eligible",
        )
    )

    mec_eligible = adults[
        pd.to_numeric(adults["WTMEC2YR"], errors="coerce") > 0
    ].copy()
    inventory.append(
        (
            "Adults >=20 y with a valid MEC exam weight",
            len(mec_eligible),
            "WTMEC2YR > 0 (examined subsample; required for the lab/anthropometric panel)",
        )
    )

    mcq = pool_component("MCQ")
    cohort = mec_eligible.rename(columns={"SEQN": "seqn"})
    cohort["seqn"] = cohort["seqn"].astype(int)

    reported_cancer = cohort[cohort["MCQ220"] == 1].copy()
    inventory.append(
        (
            "... reporting a physician diagnosis of cancer (MCQ220 = 1)",
            len(reported_cancer),
            "self-reported 'ever told you had cancer or malignancy'",
        )
    )

    events = cancer_events_long(mcq)
    primary = _primary_site(events)
    survivors = reported_cancer.merge(primary, on="seqn", how="left")

    has_site_info = survivors["site_group"].notna()
    inventory.append(
        (
            "... with an identifiable cancer site (MCQ230/MCQ240 items)",
            int(has_site_info.sum()),
            "site recoverable from the harmonized MCQ230/MCQ240 (or MCD240) items",
        )
    )

    skin_only = has_site_info & (
```

```python
        ~survivors["has_non_skin_cancer"].astype("boolean").fillna(False)
    )
    survivors_excl_skin = survivors[~skin_only].copy()
    inventory.append(
        (
            "Excluding survivors whose only reported cancer is skin, "
            "non-melanoma or unknown histology (MCQ240W/X or code 32/33)",
            int(skin_only.sum()),
            "removed: registry convention treats "
            "non-melanoma/unspecified skin cancer as a distinct, much less "
            "lethal entity that is excluded from most cancer-survivorship "
            "cohorts and from SEER incidence counts",
        )
    )

    survivors_excl_skin["age_at_diagnosis_valid"] = survivors_excl_skin[
        "age_at_diagnosis"
    ].notna() & (
        survivors_excl_skin["age_at_diagnosis"]
        <= survivors_excl_skin["RIDAGEYR"]
    )
    bad_age = (
        survivors_excl_skin["age_at_diagnosis"].notna()
        & ~survivors_excl_skin["age_at_diagnosis_valid"]
    )
    inventory.append(
        (
            "... with an implausible age at diagnosis (> current age; dropped to missing)",
            int(bad_age.sum()),
            "age_at_diagnosis > RIDAGEYR at the same or a later cycle; "
            "set to missing rather than excluding the person",
        )
    )
    survivors_excl_skin.loc[
        ~survivors_excl_skin["age_at_diagnosis_valid"], "age_at_diagnosis"
    ] = np.nan

    survivors_excl_skin["time_since_diagnosis_years"] = (
        survivors_excl_skin["RIDAGEYR"]
        - survivors_excl_skin["age_at_diagnosis"]
    )

    has_time = survivors_excl_skin["time_since_diagnosis_years"].notna()
    inventory.append(
        (
            "Survivor cohort with a computable time since diagnosis",
            int(has_time.sum()),
            "age at MEC exam minus valid age at diagnosis",
        )
    )

    inventory.append(
        (
            "FINAL analytic survivor cohort (n_survivor)",
            len(survivors_excl_skin),
            "all cancer-diagnosis-reporting, MEC-examined adults >=20y, "
            "skin-cancer-only cases excluded; site and/or time-since-diagnosis "
            "may be missing for a documented subset",
        )
    )

    survivors_full = attach_covariates(survivors_excl_skin)

    out = DATA_INTERIM / "survivor_cohort_pre_outcomes.parquet"
    survivors_full.to_parquet(out, index=False)

    inv_df = pd.DataFrame(inventory, columns=["label", "n", "description"])
    inv_df.to_csv(RESULTS_TABLES / "subsample_inventory.csv", index=False)

    return survivors_full, inv_df
```

7 src/outcomes.py

```
from __future__ import annotations

import pandas as pd

from .config import DATA_PROCESSED, LEADING_CAUSE_DETAILED_CYCLES
from .lmf_loader import load_lmf


def attach_outcomes(cohort: pd.DataFrame) -> pd.DataFrame:
    df = cohort.copy()
    lmf = load_lmf()
    lmf_cols = [
        "SEQN",
        "MORTSTAT",
        "UCOD_LEADING",
        "cv_death",
        "cv_death_broad",
        "cancer_death",
        "followup_months",
    ]
    lmf_use = lmf[lmf_cols].rename(columns={"SEQN": "seqn"})
    df = df.merge(lmf_use, on="seqn", how="left")

    df["followup_years"] = (
        pd.to_numeric(df["followup_months"], errors="coerce") / 12.0
    )

    dead = df["MORTSTAT"] == 1
    event = pd.Series(0, index=df.index, dtype="int8")
    event = event.where(~(dead & df["cv_death"]), 1)
    event = event.where(~(dead & df["cancer_death"]), 2)
    other_death = (
        dead
        & ~df["cv_death"].astype("boolean").fillna(False)
        & ~df["cancer_death"].astype("boolean").fillna(False)
    )
    event = event.where(~other_death, 3)
    df["event"] = event
    df["cvd_death"] = (df["event"] == 1).astype("Int8")
    df["cancer_death"] = (df["event"] == 2).astype("Int8")
    df["other_death"] = (df["event"] == 3).astype("Int8")
    df["any_death"] = (df["MORTSTAT"] == 1).astype("Int8")

    event_broad = pd.Series(0, index=df.index, dtype="int8")
    event_broad = event_broad.where(~(dead & df["cv_death_broad"]), 1)
    event_broad = event_broad.where(~(dead & (df["event"] == 2)), 2)
    other_broad = (
        dead
        & ~df["cv_death_broad"].astype("boolean").fillna(False)
        & (df["event"] != 2)
    )
    event_broad = event_broad.where(~other_broad, 3)
    df["event_broad_cvd"] = event_broad
    df["leading_cause_detailed_cycle"] = df["CYCLE"].isin(
        LEADING_CAUSE_DETAILED_CYCLES
    )

    out = DATA_PROCESSED / "survivor_cohort_outcomes.parquet"
    df.to_parquet(out, index=False)
    return df


def outcome_summary(df: pd.DataFrame) -> pd.DataFrame:
    n = len(df)
    n_matched = df["MORTSTAT"].notna().sum()
    rows = [
        {
            "outcome": "linked to LMF (MORTSTAT non-missing)",
            "n": int(n_matched),
            "pct_of_cohort": round(100 * n_matched / n, 1),
        },
        {
            "outcome": "any death by 2019",
            "n": int((df["event"] > 0).sum()),
```

```
            "pct_of_cohort": round(100 * (df["event"] > 0).mean(), 1),
        },
        {
            "outcome": "cardiovascular death (event=1)",
            "n": int((df["event"] == 1).sum()),
            "pct_of_cohort": round(100 * (df["event"] == 1).mean(), 1),
        },
        {
            "outcome": "cancer death (event=2)",
            "n": int((df["event"] == 2).sum()),
            "pct_of_cohort": round(100 * (df["event"] == 2).mean(), 1),
        },
        {
            "outcome": "other-cause death (event=3)",
            "n": int((df["event"] == 3).sum()),
            "pct_of_cohort": round(100 * (df["event"] == 3).mean(), 1),
        },
        {
            "outcome": "alive / censored at 2019 (event=0)",
            "n": int((df["event"] == 0).sum()),
            "pct_of_cohort": round(100 * (df["event"] == 0).mean(), 1),
        },
    ]
    return pd.DataFrame(rows)


def leading_cause_coding_by_cycle(df: pd.DataFrame) -> pd.DataFrame:
    ucod = df["UCOD_LEADING"].astype(str).str.strip().str.zfill(3)
    dead = df["MORTSTAT"] == 1
    rows = []
    for cycle, group in df.assign(ucod=ucod)[dead].groupby("CYCLE"):
        codes = group["ucod"]
        rows.append(
            {
                "cycle": cycle,
                "deaths": int(len(group)),
                "heart_001": int((codes == "001").sum()),
                "cerebrovascular_005": int((codes == "005").sum()),
                "cancer_002": int((codes == "002").sum()),
                "accident_004": int((codes == "004").sum()),
                "residual_010": int((codes == "010").sum()),
                "detailed_codes_present": bool(
                    (
                        codes.isin(["003", "005", "006", "007", "008", "009"])
                    ).any()
                ),
            }
        )
    return pd.DataFrame(rows).sort_values("cycle").reset_index(drop=True)


def broad_cvd_outcome_summary(df: pd.DataFrame) -> pd.DataFrame:
    detailed = df[df["leading_cause_detailed_cycle"]]
    rows = []
    for label, frame, column in (
        ("all ten cycles, diseases of heart", df, "event"),
        ("all ten cycles, heart or cerebrovascular", df, "event_broad_cvd"),
        ("cycles 1999-2014, diseases of heart", detailed, "event"),
        (
            "cycles 1999-2014, heart or cerebrovascular",
            detailed,
            "event_broad_cvd",
        ),
    ):
        rows.append(
            {
                "definition": label,
                "n_cohort": int(len(frame)),
                "cardiovascular_deaths": int((frame[column] == 1).sum()),
                "cancer_deaths": int((frame[column] == 2).sum()),
                "other_deaths": int((frame[column] == 3).sum()),
                "censored": int((frame[column] == 0).sum()),
            }
        )
    return pd.DataFrame(rows)
```

8 src/covariates.py

```python
from __future__ import annotations

import numpy as np
import pandas as pd

from .config import DATA_PROCESSED


def _avg_bp(df: pd.DataFrame, prefix: str) -> pd.Series:
    cols = [c for c in df.columns if c.startswith(prefix)]
    return (
        df[cols]
        .apply(pd.to_numeric, errors="coerce")
        .mean(axis=1, skipna=True)
    )


CRP_MGDL_TO_MGL = 10.0


def _harmonise_crp(df: pd.DataFrame) -> pd.Series:
    conventional = pd.to_numeric(df.get("LBXCRP", np.nan), errors="coerce")
    conventional = conventional.fillna(
        pd.to_numeric(df.get("LB2CRP", np.nan), errors="coerce")
    )
    crp_mgl = conventional * CRP_MGDL_TO_MGL
    hscrp_mgl = pd.to_numeric(df.get("LBXHSCRP", np.nan), errors="coerce")
    harmonised = crp_mgl.fillna(hscrp_mgl)
    observed = harmonised.dropna()
    if len(observed):
        median = float(observed.median())
        if not 0.5 < median < 10.0:
            raise AssertionError(
                f"Pooled CRP median {median:.2f} mg/L is outside the published NHANES "
                "adult range; check for a unit-conversion or assay-pooling error."
            )
    return harmonised


def _nlr(df: pd.DataFrame) -> pd.Series:
    neut = pd.to_numeric(df.get("LBDNENO", np.nan), errors="coerce")
    lymp = pd.to_numeric(df.get("LBDLYMNO", np.nan), errors="coerce")
    with np.errstate(divide="ignore", invalid="ignore"):
        ratio = np.where(lymp > 0, neut / lymp, np.nan)
    return pd.Series(ratio, index=df.index)


def _smoking_status(df: pd.DataFrame) -> pd.Series:
    ever = pd.to_numeric(df.get("SMQ020", np.nan), errors="coerce")
    now = pd.to_numeric(df.get("SMQ040", np.nan), errors="coerce")
    status = pd.Series(pd.NA, index=df.index, dtype="object")
    status = status.mask(ever == 2, "never")
    status = status.mask((ever == 1) & now.isin([1, 2]), "current")
    status = status.mask((ever == 1) & (now == 3), "former")
    return status


def _alcohol_ever(df: pd.DataFrame) -> pd.Series:
    a = pd.to_numeric(df.get("ALQ100", np.nan), errors="coerce")
    b = pd.to_numeric(df.get("ALD100", np.nan), errors="coerce")
    c = pd.to_numeric(df.get("ALQ101", np.nan), errors="coerce")
    d = pd.to_numeric(df.get("ALQ111", np.nan), errors="coerce")
    combined = a.fillna(b).fillna(c).fillna(d)
    combined = combined.where(combined.isin([1, 2]))
    return (combined == 1).where(combined.notna())


def _physical_activity_active(df: pd.DataFrame) -> pd.Series:
    pre2007 = pd.to_numeric(df.get("PAQ180", np.nan), errors="coerce")
    pre2007 = pre2007.where(pre2007.isin([1, 2, 3, 4]))
    pre2007_active = (pre2007 >= 3).where(pre2007.notna())
    mod = pd.to_numeric(df.get("PAQ665", np.nan), errors="coerce")
    vig = pd.to_numeric(df.get("PAQ650", np.nan), errors="coerce")
    mod, vig = mod.where(mod.isin([1, 2])), vig.where(vig.isin([1, 2]))
```

```python
    post2007_active = ((mod == 1) | (vig == 1)).where(
        mod.notna() | vig.notna()
    )
    return pre2007_active.where(pre2007_active.notna(), post2007_active)


HUQ051_TO_HUQ050 = {0: 0, 1: 1, 2: 2, 3: 3, 4: 3, 5: 3, 6: 4, 7: 5, 8: 5}


def _healthcare_visits(df: pd.DataFrame) -> pd.Series:
    a = pd.to_numeric(df.get("HUQ050", np.nan), errors="coerce")
    a = a.where(a.isin(range(6)))
    b = pd.to_numeric(df.get("HUQ051", np.nan), errors="coerce").map(
        HUQ051_TO_HUQ050
    )
    return a.fillna(b)


def _comorbidity_index(df: pd.DataFrame) -> pd.Series:
    flags = [
        "BPQ020",
        "DIQ010",
        "MCQ160B",
        "MCQ160C",
        "MCQ160D",
        "MCQ160E",
        "MCQ160F",
    ]
    out = pd.Series(0.0, index=df.index)
    for col in flags:
        if col in df.columns:
            s = pd.to_numeric(df[col], errors="coerce")
            out = out + (s == 1).astype(int)
    return out


RACE_LABELS = {
    1: "1 Mexican American",
    2: "2 Other Hispanic",
    3: "3 Non-Hispanic White",
    4: "4 Non-Hispanic Black",
    5: "5 Other or multiracial",
}
EDUCATION_LABELS = {
    1: "1 Less than 9th grade",
    2: "2 9th-11th grade",
    3: "3 High school or GED",
    4: "4 Some college",
    5: "5 College graduate or above",
}


def winsorise(
    s: pd.Series, lower: float = 0.01, upper: float = 0.99
) -> pd.Series:
    if s.dropna().empty:
        return s
    lo, hi = s.quantile([lower, upper])
    return s.clip(lower=lo, upper=hi)


def build_features(
    cohort: pd.DataFrame, out_name: str = "feature_panel.parquet"
) -> pd.DataFrame:
    df = cohort.copy()
    panel = pd.DataFrame(index=df.index)

    panel["seqn"] = df["seqn"]
    panel["cycle"] = df["CYCLE"]
    panel["weight_mec"] = pd.to_numeric(df["WTMEC2YR"], errors="coerce")
    panel["psu"] = pd.to_numeric(df["SDMVPSU"], errors="coerce")
    panel["strata"] = pd.to_numeric(df["SDMVSTRA"], errors="coerce")

    panel["age"] = pd.to_numeric(df["RIDAGEYR"], errors="coerce")
    panel["sex_female"] = (
        pd.to_numeric(df["RIAGENDR"], errors="coerce") == 2
```

```python
    ).astype("Int8")
    panel["race_eth"] = pd.to_numeric(df["RIDRETH1"], errors="coerce").map(
        RACE_LABELS
    )
    panel["education"] = pd.to_numeric(df["DMDEDUC2"], errors="coerce").map(
        EDUCATION_LABELS
    )
    panel["pir"] = winsorise(pd.to_numeric(df["INDFMPIR"], errors="coerce"))

    panel["bmi"] = winsorise(pd.to_numeric(df["BMXBMI"], errors="coerce"))
    panel["sbp"] = winsorise(_avg_bp(df, "BPXSY"))
    panel["dbp"] = winsorise(_avg_bp(df, "BPXDI"))

    panel["total_chol"] = winsorise(
        pd.to_numeric(df["LBXTC"], errors="coerce")
    )
    hdl = pd.to_numeric(df.get("LBDHDL", np.nan), errors="coerce")
    hdl = hdl.fillna(pd.to_numeric(df.get("LBDHDD", np.nan), errors="coerce"))
    hdl = hdl.fillna(pd.to_numeric(df.get("LBXHDD", np.nan), errors="coerce"))
    panel["hdl"] = winsorise(hdl)
    panel["triglycerides"] = winsorise(
        pd.to_numeric(df["LBXTR"], errors="coerce")
    )
    panel["hba1c"] = winsorise(pd.to_numeric(df["LBXGH"], errors="coerce"))
    panel["fasting_glucose"] = winsorise(
        pd.to_numeric(df["LBXGLU"], errors="coerce")
    )
    panel["crp"] = winsorise(_harmonise_crp(df))
    panel["nlr"] = winsorise(_nlr(df))
    panel["hemoglobin"] = winsorise(
        pd.to_numeric(df["LBXHGB"], errors="coerce")
    )
    panel["serum_albumin"] = winsorise(
        pd.to_numeric(df["LBXSAL"], errors="coerce")
    )

    panel["hypertension"] = (
        pd.to_numeric(df["BPQ020"], errors="coerce") == 1
    ).astype("Int8")
    panel["diabetes"] = (
        pd.to_numeric(df["DIQ010"], errors="coerce") == 1
    ).astype("Int8")
    panel["comorbidity_index"] = _comorbidity_index(df)

    panel["smoking_status"] = _smoking_status(df)
    panel["alcohol_ever_heavy"] = _alcohol_ever(df).astype("boolean")
    panel["physically_active"] = _physical_activity_active(df).astype(
        "boolean"
    )
    panel["healthcare_visits_12mo"] = _healthcare_visits(df)
    ghr = pd.to_numeric(df["HUQ010"], errors="coerce")
    panel["general_health_rating"] = ghr.where(ghr.isin([1, 2, 3, 4, 5]))

    panel["site_group"] = df["site_group"]
    panel["site_name"] = df["site_name"]
    panel["n_cancer_sites"] = pd.to_numeric(
        df["n_cancer_sites"], errors="coerce"
    )
    panel["age_at_diagnosis"] = pd.to_numeric(
        df["age_at_diagnosis"], errors="coerce"
    )
    panel["time_since_diagnosis_years"] = pd.to_numeric(
        df["time_since_diagnosis_years"], errors="coerce"
    )

    for col in (
        "event",
        "cvd_death",
        "cancer_death",
        "other_death",
        "any_death",
        "followup_years",
        "MORTSTAT",
        "event_broad_cvd",
        "leading_cause_detailed_cycle",
    ):
```

```
        if col in df.columns:
            panel[col] = df[col]

    out = DATA_PROCESSED / out_name
    panel.to_parquet(out, index=False)
    return panel
```

9 src/cardiotoxicity.py

```
from __future__ import annotations

import numpy as np
import pandas as pd

HIGH_CARDIOTOXICITY_SITES = {
    "Breast",
    "Blood",
    "Leukemia",
    "Lymphoma",
    "Prostate",
}
LOW_CARDIOTOXICITY_SITES = {
    "Skin, non-melanoma",
    "Skin, unknown type",
    "Melanoma",
    "Thyroid",
    "Cervix",
}


def cardiotoxicity_group(
    panel: pd.DataFrame, site_col: str = "site_name"
) -> pd.Series:
    site = panel[site_col]
    out = pd.Series("Moderate/unclassified", index=panel.index, dtype="object")
    out = out.mask(
        site.isin(HIGH_CARDIOTOXICITY_SITES), "High expected cardiotoxicity"
    )
    out = out.mask(
        site.isin(LOW_CARDIOTOXICITY_SITES), "Low expected cardiotoxicity"
    )
    out = out.mask(site.isna(), np.nan)
    return out


def e_value(estimate: float, ci_bound: float | None = None) -> dict:
    def _ev(rr: float) -> float:
        if rr is None or not np.isfinite(rr):
            return float("nan")
        r = rr if rr >= 1 else 1.0 / rr
        return r + np.sqrt(r * (r - 1))

    out = {"estimate": estimate, "e_value_point": round(_ev(estimate), 2)}
    if ci_bound is not None:
        out["ci_bound"] = ci_bound
        out["e_value_ci"] = round(_ev(ci_bound), 2)
    return out


def interpret_e_value(ev_point: float, ev_ci: float | None, hr: float) -> str:
    direction = "increases" if hr >= 1 else "decreases"
    txt = (
        f"An unmeasured confounder (such as anthracycline, trastuzumab, chest "
        f"radiotherapy, or androgen-deprivation-therapy exposure) that both "
        f"{direction} the risk of cardiovascular death and is associated with "
        f"high-cardiotoxicity cancer sites by a risk-ratio of at least "
        f"{ev_point:.2f}-fold, on both counts, could fully explain away the "
        f"observed association; a weaker confounder could not."
    )
    if ev_ci is not None and np.isfinite(ev_ci):
        txt += (
            f" Using the confidence bound closer to the null instead of the "
            f"point estimate, a confounder of at least {ev_ci:.2f}-fold on "
```

```
            f"both counts would be needed -- a level of confounding that "
            f"documented cardio-oncology treatment effects (e.g. anthracycline "
            f"or trastuzumab cardiotoxicity) plausibly could reach, which is "
            f"exactly why treatment exposure, not measured here, remains the "
            f"leading alternative explanation for this contrast."
        )
    return txt
```

10 src/stratify.py

```
from __future__ import annotations

import numpy as np
import pandas as pd

from src import survey

from .config import RESULTS_TABLES

STRATUM_MIN_N_DESCRIPTIVE = 30
STRATUM_MIN_N_MODEL = 100

TIME_SINCE_DX_BINS = [0, 2, 5, 10, np.inf]
TIME_SINCE_DX_LABELS = ["under2y", "2to5y", "5to10y", "10y_or_more"]
TIME_SINCE_DX_DISPLAY = {
    "under2y": "<2 y",
    "2to5y": "2-<5 y",
    "5to10y": "5-<10 y",
    "10y_or_more": ">=10 y",
}


def time_since_dx_strata(panel: pd.DataFrame) -> dict[str, pd.Series]:
    cut = pd.cut(
        panel["time_since_diagnosis_years"],
        TIME_SINCE_DX_BINS,
        labels=TIME_SINCE_DX_LABELS,
        right=False,
    )
    return {lab: (cut == lab) for lab in TIME_SINCE_DX_LABELS}


def site_strata(panel: pd.DataFrame) -> dict[str, pd.Series]:
    sites = panel["site_group"].dropna().unique().tolist()
    return {s: (panel["site_group"] == s) for s in sorted(sites)}


def stratum_report(
    panel: pd.DataFrame,
    strata: dict[str, pd.Series],
    label_col: str,
    n_cycles: int = 10,
    shap_features: list[str] | None = None,
) -> pd.DataFrame:
    rows = []
    total_n = len(panel)
    total_mask_sum = 0
    for label, mask in strata.items():
        mask = mask.reindex(panel.index).fillna(False).astype(bool)
        n = int(mask.sum())
        total_mask_sum += n
        if n == 0:
            rows.append(
                {
                    "stratum": label,
                    "n": 0,
                    "n_shap_eligible": 0,
                    "weighted_pct_of_cohort": 0.0,
                    "n_cvd_death": 0,
                    "n_cancer_death": 0,
                    "n_other_death": 0,
                    "supports_descriptive": False,
                    "supports_model": False,
```

```
                    "supports_shap": False,
                }
            )
            continue
        sub = panel.loc[mask]
        frame = pd.DataFrame(
            {
                "SDMVPSU": panel["psu"],
                "SDMVSTRA": panel["strata"],
                "WTMEC2YR": panel["weight_mec"],
            },
            index=panel.index,
        )
        d_full = survey.build(frame, "mec", n_cycles=n_cycles)
        in_stratum_weighted = (
            d_full.weight.reindex(mask.index).where(mask, 0).sum()
        )
        weighted_pct = 100 * in_stratum_weighted / d_full.weight.sum()
        if shap_features:
            n_shap = int(sub[shap_features].notna().all(axis=1).sum())
        else:
            n_shap = n
        rows.append(
            {
                "stratum": label,
                "n": n,
                "n_shap_eligible": n_shap,
                "weighted_pct_of_cohort": round(float(weighted_pct), 1),
                "n_cvd_death": int((sub["event"] == 1).sum()),
                "n_cancer_death": int((sub["event"] == 2).sum()),
                "n_other_death": int((sub["event"] == 3).sum()),
                "supports_descriptive": n >= STRATUM_MIN_N_DESCRIPTIVE,
                "supports_model": n >= STRATUM_MIN_N_MODEL,
                "supports_shap": n_shap >= 50,
            }
        )
    out = pd.DataFrame(rows)
    if total_mask_sum > total_n:
        raise AssertionError(
            f"{label_col} stratum n's sum to {total_mask_sum}, exceeding cohort n={total_n} "
            "-- strata are not mutually exclusive, check the definition"
        )
    out.to_csv(RESULTS_TABLES / f"stratum_report_{label_col}.csv", index=False)
    return out
```

11 src/survey.py

```
from __future__ import annotations

from dataclasses import dataclass

import numpy as np
import pandas as pd

PSU = "SDMVPSU"
STRATUM = "SDMVSTRA"

WEIGHTS = {
    "mec": "WTMEC2YR",
    "interview": "WTINT2YR",
    "fasting": "WTSAF2YR",
    "diet_day1": "WTDRD1",
    "diet_2day": "WTDR2D",
}


@dataclass(frozen=True)
class Design:
    weight: pd.Series
    psu: pd.Series
    stratum: pd.Series
    n_cycles: int
    weight_name: str
```

```python

    def __post_init__(self) -> None:
        if not (self.weight > 0).all():
            raise ValueError(
                "non-positive survey weights present; drop those rows first"
            )

    @property
    def n(self) -> int:
        return len(self.weight)

    def methods_sentence(
        self, variance: str = "the stratified delete-one-PSU jackknife"
    ) -> str:
        return (
            f"All estimates account for the complex, multistage probability sampling design of "
            f"NHANES. Analyses used the {self.weight_name} sampling weight divided by "
            f"{self.n_cycles} to reflect the pooling of {self.n_cycles} two-year cycles, with "
            f"{STRATUM} as the stratum identifier and {PSU} as the primary sampling unit. "
            f"Variance estimation used {variance}, and the design was applied to every "
            f"estimator reported, including the machine-learning models and their confidence "
            f"intervals, not to descriptive statistics alone."
        )


def build(df: pd.DataFrame, kind: str, n_cycles: int) -> Design:
    if kind not in WEIGHTS:
        raise KeyError(
            f"unknown weight kind {kind!r}; choose from {sorted(WEIGHTS)}"
        )
    col = WEIGHTS[kind]
    for required in (col, PSU, STRATUM):
        if required not in df.columns:
            raise KeyError(
                f"{required} missing from frame; it is required for design-based analysis"
            )
    keep = df[col].notna() & (df[col] > 0)
    sub = df.loc[keep]
    return Design(
        weight=sub[col] / n_cycles,
        psu=sub[PSU],
        stratum=sub[STRATUM],
        n_cycles=n_cycles,
        weight_name=col,
    )


def wmean(x: pd.Series, d: Design) -> float:
    x, w = _align(x, d)
    return float(np.sum(w * x) / np.sum(w))


def wquantile(x: pd.Series, d: Design, q: float) -> float:
    x, w = _align(x, d)
    order = np.argsort(x)
    x, w = x[order], w[order]
    cw = np.cumsum(w) - 0.5 * w
    cw /= np.sum(w)
    return float(np.interp(q, cw, x))


def wprop(mask: pd.Series, d: Design) -> float:
    return wmean(mask.astype(float), d)


def _align(x: pd.Series, d: Design) -> tuple[np.ndarray, np.ndarray]:
    idx = x.index.intersection(d.weight.index)
    xv = x.loc[idx].astype(float)
    wv = d.weight.loc[idx].astype(float)
    ok = xv.notna()
    return xv[ok].to_numpy(), wv[ok].to_numpy()


def jackknife_replicates(d: Design) -> tuple[np.ndarray, list[tuple]]:
    frame = pd.DataFrame({"w": d.weight, "psu": d.psu, "stratum": d.stratum})
    units = sorted(
```

```
        map(tuple, frame[["stratum", "psu"]].drop_duplicates().to_numpy())
    )
    n_psu_by_stratum = frame.groupby("stratum")["psu"].nunique().to_dict()

    stratum_arr = frame["stratum"].to_numpy()
    psu_arr = frame["psu"].to_numpy()
    base_w = frame["w"].to_numpy()

    keep: list[tuple] = []
    rows: list[np.ndarray] = []
    for stratum, psu in units:
        n_h = n_psu_by_stratum[stratum]
        if n_h < 2:
            continue
        w = base_w.copy()
        in_stratum = stratum_arr == stratum
        dropped = in_stratum & (psu_arr == psu)
        w[dropped] = 0.0
        w[in_stratum & ~dropped] *= n_h / (n_h - 1)
        rows.append(w)
        keep.append((stratum, psu))
    if not rows:
        raise ValueError(
            "no stratum has >=2 PSUs; design-based variance is not estimable"
        )
    return np.vstack(rows), keep


def _subset_strata(
    reps: np.ndarray, units: list[tuple], max_units: int | None
) -> tuple[np.ndarray, list[tuple], float]:
    if max_units is None or len(units) <= max_units:
        return reps, units, 1.0
    strata_order: list = []
    for stratum, _psu in units:
        if stratum not in strata_order:
            strata_order.append(stratum)
    sizes = {s: sum(1 for st, _ in units if st == s) for s in strata_order}
    mean_size = max(sum(sizes.values()) / len(sizes), 1.0)
    n_keep = max(int(max_units // mean_size), 2)
    if n_keep >= len(strata_order):
        return reps, units, 1.0
    step = len(strata_order) / n_keep
    chosen = {
        strata_order[min(int(i * step), len(strata_order) - 1)]
        for i in range(n_keep)
    }
    idx = [i for i, (s, _) in enumerate(units) if s in chosen]
    if len(idx) < 2:
        return reps, units, 1.0
    return reps[idx], [units[i] for i in idx], len(strata_order) / len(chosen)


def _warn_failed(n_failed: int, n_total: int, label: str = "") -> None:
    if n_failed:
        import warnings as _w

        _w.warn(
            f"design jackknife{(' ' + label) if label else ''}: {n_failed} of {n_total} "
            "replicate refits failed and were dropped; report this count",
            RuntimeWarning,
            stacklevel=3,
        )


def design_jackknife_ci(
    statistic,
    d: Design,
    *,
    alpha: float = 0.05,
    max_units: int | None = None,
) -> tuple[float, float, float]:
    from scipy import stats as _st

    point = float(statistic(d.weight.to_numpy()))
    reps, units = jackknife_replicates(d)
```

```python
    reps, units, scale = _subset_strata(reps, units, max_units)

    theta = np.array(
        [statistic(reps[r]) for r in range(len(reps))], dtype=float
    )
    ok = np.isfinite(theta)
    _warn_failed(int((~ok).sum()), len(theta))
    theta, units = theta[ok], [u for u, k in zip(units, ok, strict=False) if k]
    if len(theta) < 2:
        return point, float("nan"), float("nan")

    by_stratum: dict = {}
    for value, (stratum, _psu) in zip(theta, units, strict=False):
        by_stratum.setdefault(stratum, []).append(value)

    variance = 0.0
    n_units = 0
    for values in by_stratum.values():
        n_h = len(values)
        if n_h < 2:
            continue
        arr = np.asarray(values, dtype=float)
        variance += (n_h - 1) / n_h * float(np.sum((arr - arr.mean()) ** 2))
        n_units += n_h
    df = max(n_units - len(by_stratum), 1)
    half = float(_st.t.ppf(1 - alpha / 2, df)) * float(
        np.sqrt(variance * scale)
    )
    return point, point - half, point + half


def design_jackknife_ci_multi(
    statistic,
    d: Design,
    *,
    alpha: float = 0.05,
    max_units: int | None = None,
) -> dict[str, tuple[float, float, float]]:
    from scipy import stats as _st

    point = statistic(d.weight.to_numpy())
    if not isinstance(point, dict):
        raise TypeError("statistic must return a dict of {name: value}")
    reps, units = jackknife_replicates(d)
    reps, units, scale = _subset_strata(reps, units, max_units)

    rep_values = [statistic(reps[r]) for r in range(len(reps))]

    out: dict[str, tuple[float, float, float]] = {}
    for key, pt in point.items():
        theta = np.array(
            [
                (
                    rv.get(key, float("nan"))
                    if isinstance(rv, dict)
                    else float("nan")
                )
                for rv in rep_values
            ],
            dtype=float,
        )
        ok = np.isfinite(theta)
        _warn_failed(int((~ok).sum()), len(theta), label=str(key))
        vals, us = theta[ok], [u for u, k in zip(units, ok, strict=False) if k]
        if len(vals) < 2:
            out[key] = (float(pt), float("nan"), float("nan"))
            continue
        by_stratum: dict = {}
        for value, (stratum, _psu) in zip(vals, us, strict=False):
            by_stratum.setdefault(stratum, []).append(value)
        variance = 0.0
        n_units = 0
        for values in by_stratum.values():
            n_h = len(values)
            if n_h < 2:
                continue
```

```
            arr = np.asarray(values, dtype=float)
            variance += (
                (n_h - 1) / n_h * float(np.sum((arr - arr.mean()) ** 2))
            )
            n_units += n_h
        df = max(n_units - len(by_stratum), 1)
        half = float(_st.t.ppf(1 - alpha / 2, df)) * float(
            np.sqrt(variance * scale)
        )
        out[key] = (float(pt), float(pt) - half, float(pt) + half)
    return out


def subsample_inventory(rows: list[tuple[str, int, str]]) -> str:
    lines = ["| Label | *n* | Definition |", "|---|---|---|"]
    for label, n, desc in rows:
        lines.append(
            f"| {label} | {n:,} | {desc}|"
            if n >= 10000
            else f"| {label} | {n} | {desc} |"
        )
    return "\n".join(lines)
```

12 src/table1.py

```
from __future__ import annotations

import pandas as pd

from src import survey

from .config import RESULTS_TABLES

GROUP_LABELS = {
    0: "Alive/censored",
    1: "CVD death",
    2: "Cancer death",
    3: "Other-cause death",
}

CONTINUOUS = [
    "age",
    "bmi",
    "sbp",
    "dbp",
    "total_chol",
    "hdl",
    "hba1c",
    "crp",
    "hemoglobin",
    "serum_albumin",
    "pir",
    "comorbidity_index",
    "time_since_diagnosis_years",
    "n_cancer_sites",
]
BINARY = [
    "sex_female",
    "hypertension",
    "diabetes",
    "alcohol_ever_heavy",
    "physically_active",
]
CATEGORICAL = ["smoking_status", "site_group"]


def _design_for(panel: pd.DataFrame, n_cycles: int = 10) -> survey.Design:
    frame = pd.DataFrame(
        {
            "SDMVPSU": panel["psu"],
            "SDMVSTRA": panel["strata"],
            "WTMEC2YR": panel["weight_mec"],
        },
```

```python
        index=panel.index,
    )
    return survey.build(frame, "mec", n_cycles=n_cycles)


def build_table1(panel: pd.DataFrame, n_cycles: int = 10) -> pd.DataFrame:
    rows = []
    groups = {"Overall": panel.index}
    for code, label in GROUP_LABELS.items():
        groups[label] = panel.index[panel["event"] == code]

    n_check = sum(len(idx) for k, idx in groups.items() if k != "Overall")
    if n_check != len(panel):
        raise AssertionError(
            f"Table 1 subgroup n's ({n_check}) do not sum to total ({len(panel)})"
        )

    for col in CONTINUOUS:
        row = {"variable": col, "type": "continuous"}
        for label, idx in groups.items():
            sub = panel.loc[idx]
            if sub[col].notna().sum() == 0:
                row[f"{label}_n"] = int(sub[col].notna().sum())
                row[f"{label}_stat"] = "NA"
                continue
            d = _design_for(sub, n_cycles)
            m = survey.wquantile(sub[col], d, 0.50)
            lo = survey.wquantile(sub[col], d, 0.25)
            hi = survey.wquantile(sub[col], d, 0.75)
            if not (lo <= m <= hi):
                raise AssertionError(
                    f"Table 1 centre for '{col}' ({label}): median {m:.3f} lies "
                    f"outside its IQR [{lo:.3f}, {hi:.3f}]."
                )
            row[f"{label}_n"] = int(sub[col].notna().sum())
            row[f"{label}_stat"] = f"{m:.1f} [{lo:.1f}, {hi:.1f}]"
        rows.append(row)

    for col in BINARY:
        row = {"variable": col, "type": "binary"}
        for label, idx in groups.items():
            sub = panel.loc[idx]
            s = sub[col]
            if str(s.dtype) == "boolean":
                s = s.astype("float")
            valid = s.notna().sum()
            if valid == 0:
                row[f"{label}_n"] = 0
                row[f"{label}_stat"] = "NA"
                continue
            d = _design_for(sub, n_cycles)
            p = survey.wprop(s.fillna(False).astype(bool), d)
            row[f"{label}_n"] = int(valid)
            row[f"{label}_stat"] = f"{100 * p:.1f}%"
        rows.append(row)

    for col in CATEGORICAL:
        cats = panel[col].dropna().unique().tolist()
        for cat in sorted(cats, key=str):
            row = {"variable": f"{col} = {cat}", "type": "categorical"}
            for label, idx in groups.items():
                sub = panel.loc[idx]
                valid = sub[col].notna().sum()
                if valid == 0:
                    row[f"{label}_n"] = 0
                    row[f"{label}_stat"] = "NA"
                    continue
                d = _design_for(sub, n_cycles)
                p = survey.wprop(sub[col] == cat, d)
                row[f"{label}_n"] = int((sub[col] == cat).sum())
                row[f"{label}_stat"] = f"{100 * p:.1f}%"
            rows.append(row)

    n_row = {"variable": "Unweighted n", "type": "n"}
    for label, idx in groups.items():
        n_row[f"{label}_n"] = len(idx)
```

```
        n_row[f"{label}_stat"] = str(len(idx))
    rows.insert(0, n_row)

    out = pd.DataFrame(rows)
    out.to_csv(RESULTS_TABLES / "table1_design_weighted.csv", index=False)
    return out
```

13 src/audits.py

```
from __future__ import annotations

import pandas as pd
from statsmodels.stats.outliers_influence import variance_inflation_factor

from .config import RESULTS_TABLES

MODEL_FEATURES: list[str] = [
    "age",
    "sex_female",
    "race_eth",
    "education",
    "pir",
    "bmi",
    "sbp",
    "dbp",
    "total_chol",
    "hdl",
    "hba1c",
    "crp",
    "nlr",
    "hemoglobin",
    "serum_albumin",
    "hypertension",
    "diabetes",
    "comorbidity_index",
    "smoking_status",
    "alcohol_ever_heavy",
    "physically_active",
    "healthcare_visits_12mo",
    "general_health_rating",
    "time_since_diagnosis_years",
    "n_cancer_sites",
]

LEAKAGE_EXCLUDED: dict[str, str] = {
    "age_at_diagnosis": "algebraically redundant with age and time_since_diagnosis_years "
    "(age = age_at_diagnosis + time_since_diagnosis_years exactly); "
    "including all three gives infinite VIF",
    "followup_years": "the survival TIME variable itself -- using it as a predictor of "
    "the event that defines it is direct leakage",
    "MORTSTAT": "identical information to the event/outcome columns",
    "UCOD_LEADING": "the raw cause-of-death code the outcome is derived from",
    "cv_death": "component of the outcome definition",
    "cancer_death_flag_raw": "component of the outcome definition (note: 'cancer_death' "
    "the LMF flag and 'cancer_death' the outcome column share a "
    "name pre-merge in outcomes.py; the LMF flag is dropped, only "
    "the outcome column survives under that name)",
    "site_group": "categorical version of the site information; used for STRATIFICATION, "
    "not as a model feature, to avoid a 10+ level one-hot block dominating "
    "a cohort of n<4000",
    "site_name": "same reason as site_group, at even higher cardinality",
    "n_cancer_sites": "kept -- reviewed and retained: a count of reported diagnoses is not "
    "definitionally downstream of which cause a person eventually dies of",
}


def leakage_audit() -> pd.DataFrame:
    rows = [{"feature": f, "kept": True, "note": ""} for f in MODEL_FEATURES]
    rows += [
        {"feature": f, "kept": False, "note": note}
        for f, note in LEAKAGE_EXCLUDED.items()
        if f != "n_cancer_sites"
    ]
```

```python
    out = pd.DataFrame(rows)
    out.to_csv(RESULTS_TABLES / "leakage_audit.csv", index=False)
    return out


def vif_table(
    panel: pd.DataFrame, features: list[str] | None = None
) -> pd.DataFrame:
    features = features or MODEL_FEATURES
    X = panel[features].copy()
    X = X.dropna()
    for col in X.columns:
        if X[col].dtype == "object" or str(X[col].dtype) == "boolean":
            X[col] = X[col].astype("category")
    X = pd.get_dummies(X, drop_first=True)
    X = X.apply(pd.to_numeric, errors="coerce")
    X = X.dropna()
    X.insert(0, "const", 1.0)
    vifs = []
    for i, col in enumerate(X.columns):
        if col == "const":
            continue
        try:
            v = variance_inflation_factor(X.to_numpy(dtype=float), i)
        except Exception:
            v = float("nan")
        vifs.append({"feature": col, "vif": round(v, 2), "n_complete": len(X)})
    out = (
        pd.DataFrame(vifs)
        .sort_values("vif", ascending=False)
        .reset_index(drop=True)
    )
    out.to_csv(RESULTS_TABLES / "vif_table.csv", index=False)
    return out


def table1_sanity(
    panel: pd.DataFrame,
    group_col: str = "event",
    clinical_ranges: dict[str, tuple[float, float]] | None = None,
) -> tuple[pd.DataFrame, list[str]]:
    clinical_ranges = clinical_ranges or {
        "age": (20, 90),
        "bmi": (12, 70),
        "sbp": (70, 250),
        "dbp": (30, 150),
        "total_chol": (50, 500),
        "hdl": (10, 150),
        "hba1c": (3, 18),
        "crp": (0, 200),
        "hemoglobin": (5, 22),
        "serum_albumin": (1, 6),
    }
    problems: list[str] = []
    rows = []

    n_total = len(panel)
    group_counts = panel[group_col].value_counts(dropna=False)
    if group_counts.sum() != n_total:
        problems.append(
            f"subgroup counts ({group_counts.sum()}) do not sum to total ({n_total})"
        )

    binaries = [
        "sex_female",
        "hypertension",
        "diabetes",
        "alcohol_ever_heavy",
        "physically_active",
        "cvd_death",
        "cancer_death",
        "other_death",
        "any_death",
    ]
    for col in binaries:
        if col not in panel.columns:
```

```python
            continue
        s = pd.to_numeric(panel[col], errors="coerce").dropna()
        if s.empty:
            continue
        p = s.mean()
        rows.append(
            {"check": "binary_prevalence", "column": col, "value": round(p, 4)}
        )
        if p <= 0.0 or p >= 1.0:
            problems.append(
                f"{col} is {p:.1%} -- 0%/100% binary, looks like a pipeline bug"
            )

    numeric_candidates = [
        "age",
        "bmi",
        "sbp",
        "dbp",
        "total_chol",
        "hdl",
        "hba1c",
        "crp",
        "nlr",
        "hemoglobin",
        "serum_albumin",
        "pir",
        "comorbidity_index",
        "healthcare_visits_12mo",
        "time_since_diagnosis_years",
        "n_cancer_sites",
    ]
    for col in numeric_candidates:
        if col not in panel.columns:
            continue
        s = pd.to_numeric(panel[col], errors="coerce").dropna()
        if s.empty:
            continue
        var = s.var()
        rows.append(
            {"check": "variance", "column": col, "value": round(float(var), 4)}
        )
        if var == 0:
            problems.append(f"{col} has zero variance")
        if col in clinical_ranges:
            lo, hi = clinical_ranges[col]
            m = s.mean()
            rows.append(
                {
                    "check": "mean_in_range",
                    "column": col,
                    "value": round(float(m), 2),
                }
            )
            if not (lo <= m <= hi):
                problems.append(
                    f"{col} mean {m:.2f} outside clinical range [{lo}, {hi}]"
                )

    out = pd.DataFrame(rows)
    out.to_csv(RESULTS_TABLES / "table1_sanity_checks.csv", index=False)
    (RESULTS_TABLES / "table1_sanity_problems.txt").write_text(
        "\n".join(problems) if problems else "no problems found"
    )
    return out, problems
```

14 src/finegray.py

```python
from __future__ import annotations

import numpy as np
import pandas as pd
from lifelines import CoxTimeVaryingFitter

```

```python
from src import survey


def _censoring_survival(times: np.ndarray, is_censored: np.ndarray):
    grid = np.unique(times)
    n_risk = np.array([(times >= t).sum() for t in grid], dtype=float)
    d_cens = np.array(
        [((times == t) & is_censored).sum() for t in grid], dtype=float
    )
    with np.errstate(divide="ignore", invalid="ignore"):
        factor = np.where(n_risk > 0, 1.0 - d_cens / n_risk, 1.0)
    g = np.cumprod(factor)
    return grid, g


def _g_lookup(grid: np.ndarray, g: np.ndarray, t):
    idx = np.searchsorted(grid, t, side="right") - 1
    if np.isscalar(t):
        return 1.0 if idx < 0 else float(g[idx])
    out = np.where(idx < 0, 1.0, g[np.clip(idx, 0, len(g) - 1)])
    return out


def finegray_transform(
    df: pd.DataFrame,
    time_col: str,
    event_col: str,
    cause: int,
    id_col: str = "seqn",
) -> pd.DataFrame:
    d = df[[id_col, time_col, event_col]].dropna().copy()
    d[time_col] = d[time_col].astype(float)
    times = d[time_col].to_numpy()
    events = d[event_col].to_numpy()
    ids = d[id_col].to_numpy()

    is_censored = events == 0
    grid, g = _censoring_survival(times, is_censored)
    cause_times = np.unique(times[events == cause])

    rows = []
    for t_i, e_i, id_i in zip(times, events, ids, strict=False):
        if e_i == cause:
            rows.append((id_i, 0.0, t_i, 1, 1.0))
        elif e_i == 0:
            rows.append((id_i, 0.0, t_i, 0, 1.0))
        else:
            rows.append((id_i, 0.0, t_i, 0, 1.0))
            g_di = _g_lookup(grid, g, t_i)
            if g_di <= 0:
                continue
            ext = cause_times[cause_times > t_i]
            prev = t_i
            for t_j in ext:
                w = _g_lookup(grid, g, t_j) / g_di
                if w <= 1e-6:
                    break
                rows.append((id_i, prev, t_j, 0, w))
                prev = t_j
    long_df = pd.DataFrame(
        rows, columns=[id_col, "tstart", "tstop", "fgstatus", "fgwt"]
    )
    long_df = long_df[long_df["tstart"] < long_df["tstop"]]
    return long_df.reset_index(drop=True)


def fit_finegray_cox(
    panel: pd.DataFrame,
    features: list[str],
    time_col: str,
    event_col: str,
    cause: int,
    design: survey.Design,
    id_col: str = "seqn",
    penalizer: float = 0.1,
) -> tuple[CoxTimeVaryingFitter, pd.DataFrame]:
```

```
    d = panel.dropna(subset=features + [time_col, event_col, id_col]).copy()
    long_df = finegray_transform(d, time_col, event_col, cause, id_col=id_col)

    survey_w = pd.Series(
        design.weight.to_numpy(),
        index=panel.loc[design.weight.index, id_col].to_numpy(),
    )
    long_df["design_w"] = long_df[id_col].map(survey_w)
    long_df = long_df.dropna(subset=["design_w"])
    long_df["row_weight"] = long_df["fgwt"] * long_df["design_w"]

    feat = d.set_index(id_col)[features]
    feat_encoded = pd.get_dummies(
        feat,
        columns=[
            c
            for c in features
            if feat[c].dtype == "object" or str(feat[c].dtype) == "boolean"
        ],
        drop_first=True,
    )
    feat_encoded = feat_encoded.apply(pd.to_numeric, errors="coerce")
    long_df = long_df.join(feat_encoded, on=id_col)
    long_df = long_df.dropna(subset=list(feat_encoded.columns))

    model_cols = [id_col, "tstart", "tstop", "fgstatus", "row_weight"] + list(
        feat_encoded.columns
    )
    fit_df = long_df[model_cols]

    ctv = CoxTimeVaryingFitter(penalizer=penalizer)
    ctv.fit(
        fit_df,
        event_col="fgstatus",
        start_col="tstart",
        stop_col="tstop",
        weights_col="row_weight",
        id_col=id_col,
        robust=False,
        show_progress=False,
    )
    return ctv, long_df


def hr_table(ctv: CoxTimeVaryingFitter) -> pd.DataFrame:
    s = ctv.summary.copy()
    s["HR"] = np.exp(s["coef"])
    s["HR_lo"] = np.exp(s["coef lower 95%"])
    s["HR_hi"] = np.exp(s["coef upper 95%"])
    s["ci_note"] = (
        "Model-based interval from the weighted Cox fit, which treats the "
        "survey weights as frequencies and so understates the variance. "
        "The interval reported in the manuscript is the design-aware "
        "delete-one-PSU jackknife interval in the companion "
        "*_design_ci.csv file."
    )
    return (
        s[["coef", "se(coef)", "p", "HR", "HR_lo", "HR_hi", "ci_note"]]
        .reset_index()
        .rename(columns={"index": "feature", "covariate": "feature"})
    )


def design_ci_for_coefficients(
    panel: pd.DataFrame,
    features: list[str],
    time_col: str,
    event_col: str,
    cause: int,
    design: survey.Design,
    coefficients: list[str],
    id_col: str = "seqn",
    max_units: int = 40,
    penalizer: float = 0.1,
) -> pd.DataFrame:
    import gc
```

```python

    from scipy import stats as _st

    d = panel.dropna(subset=features + [time_col, event_col, id_col]).copy()
    long_df_base = finegray_transform(
        d, time_col, event_col, cause, id_col=id_col
    )

    feat = d.set_index(id_col)[features]
    feat_encoded = pd.get_dummies(
        feat,
        columns=[
            c
            for c in features
            if feat[c].dtype == "object" or str(feat[c].dtype) == "boolean"
        ],
        drop_first=True,
    )
    feat_encoded = feat_encoded.apply(pd.to_numeric, errors="coerce")
    long_df_base = long_df_base.join(feat_encoded, on=id_col)
    long_df_base = long_df_base.dropna(
        subset=list(feat_encoded.columns)
    ).reset_index(drop=True)
    feature_cols = list(feat_encoded.columns)

    id_arr = long_df_base[id_col].to_numpy()
    tstart_arr = long_df_base["tstart"].to_numpy(dtype="float32")
    tstop_arr = long_df_base["tstop"].to_numpy(dtype="float32")
    status_arr = long_df_base["fgstatus"].to_numpy(dtype="int8")
    fgwt_arr = long_df_base["fgwt"].to_numpy(dtype="float32")
    feat_mat = long_df_base[feature_cols].to_numpy(dtype="float32")
    del long_df_base
    gc.collect()

    seqn_order = d.loc[design.weight.index, id_col].to_numpy()
    seqn_to_pos = {s: i for i, s in enumerate(seqn_order)}
    pos_arr = np.array([seqn_to_pos.get(s, -1) for s in id_arr])
    valid_id = pos_arr >= 0
    pos_safe = np.clip(pos_arr, 0, None)

    def _fit_once(w_vec: np.ndarray) -> dict[str, float]:
        design_w = np.where(valid_id, w_vec[pos_safe], 0.0)
        keep = valid_id & (design_w > 0)
        row_weight = (fgwt_arr[keep] * design_w[keep]).astype("float32")
        data = {
            id_col: id_arr[keep],
            "tstart": tstart_arr[keep],
            "tstop": tstop_arr[keep],
            "fgstatus": status_arr[keep],
            "row_weight": row_weight,
        }
        for j, c in enumerate(feature_cols):
            data[c] = feat_mat[keep, j]
        ldf = pd.DataFrame(data)
        ctv = CoxTimeVaryingFitter(penalizer=penalizer)
        ctv.fit(
            ldf,
            event_col="fgstatus",
            start_col="tstart",
            stop_col="tstop",
            weights_col="row_weight",
            id_col=id_col,
            robust=False,
            show_progress=False,
        )
        return {
            c: (
                float(ctv.summary.loc[c, "coef"])
                if c in ctv.summary.index
                else float("nan")
            )
            for c in coefficients
        }

    def _fit_coefs(w_vec: np.ndarray, retries: int = 3) -> dict[str, float]:
        for _attempt in range(retries):
```

```
            try:
                return _fit_once(w_vec)
            except MemoryError:
                gc.collect()
                continue
            except Exception:
                return {c: float("nan") for c in coefficients}
        return {c: float("nan") for c in coefficients}

    point_est = _fit_coefs(design.weight.to_numpy())
    reps, units = survey.jackknife_replicates(design)
    reps, units, scale = survey._subset_strata(reps, units, max_units)

    rep_coefs = []
    for r, rep in enumerate(reps):
        rep_coefs.append(_fit_coefs(rep))
        if r % 5 == 0:
            gc.collect()

    rows = []
    for c in coefficients:
        theta = np.array([rc[c] for rc in rep_coefs], dtype=float)
        ok = np.isfinite(theta)
        theta_ok = theta[ok]
        units_ok = [u for u, k in zip(units, ok, strict=False) if k]
        by_stratum: dict = {}
        for value, (stratum, _psu) in zip(theta_ok, units_ok, strict=False):
            by_stratum.setdefault(stratum, []).append(value)
        variance = 0.0
        n_units = 0
        for values in by_stratum.values():
            n_h = len(values)
            if n_h < 2:
                continue
            arr = np.asarray(values, dtype=float)
            variance += (
                (n_h - 1) / n_h * float(np.sum((arr - arr.mean()) ** 2))
            )
            n_units += n_h
        df = max(n_units - len(by_stratum), 1)
        point = point_est[c]
        if len(theta_ok) < 2 or not np.isfinite(point):
            rows.append(
                {
                    "feature": c,
                    "coef": point,
                    "coef_lo": float("nan"),
                    "coef_hi": float("nan"),
                    "n_jackknife_units": len(theta_ok),
                    "n_failed_replicates": int((~ok).sum()),
                    "n_strata": len(by_stratum),
                    "variance_scale": scale,
                    "df": df,
                }
            )
            continue
        half = float(_st.t.ppf(0.975, df)) * float(np.sqrt(variance * scale))
        rows.append(
            {
                "feature": c,
                "coef": point,
                "coef_lo": point - half,
                "coef_hi": point + half,
                "n_jackknife_units": len(theta_ok),
                "n_failed_replicates": int((~ok).sum()),
                "n_strata": len(by_stratum),
                "variance_scale": scale,
                "df": df,
            }
        )
    out = pd.DataFrame(rows)
    out["HR"] = np.exp(out["coef"])
    out["HR_lo"] = np.exp(out["coef_lo"])
    out["HR_hi"] = np.exp(out["coef_hi"])
    return out
```

15 src/finegray_truncated.py

```python
from __future__ import annotations

import gc

import numpy as np
import pandas as pd
from lifelines import CoxTimeVaryingFitter
from scipy import stats as _st

from src import survey


def observation_probability(
    entry: np.ndarray, exit_time: np.ndarray, is_censored: np.ndarray
) -> tuple[np.ndarray, np.ndarray]:
    grid = np.unique(np.concatenate([entry, exit_time]))
    at_risk_open = np.array(
        [((entry < t) & (exit_time >= t)).sum() for t in grid], dtype=float
    )
    at_risk_closed = np.array(
        [((entry <= t) & (exit_time >= t)).sum() for t in grid], dtype=float
    )
    censored_at = np.array(
        [((exit_time == t) & is_censored).sum() for t in grid], dtype=float
    )
    entered_at = np.array([(entry == t).sum() for t in grid], dtype=float)

    with np.errstate(divide="ignore", invalid="ignore"):
        censoring_factor = np.where(
            at_risk_open > 0, 1.0 - censored_at / at_risk_open, 1.0
        )
        entry_factor = np.where(
            at_risk_closed > 0, 1.0 - entered_at / at_risk_closed, 1.0
        )
    censoring_survival = np.cumprod(censoring_factor)
    entry_distribution = np.cumprod(entry_factor[::-1])[::-1]
    return grid, censoring_survival * entry_distribution


def _lookup(grid: np.ndarray, values: np.ndarray, time) -> float:
    position = np.searchsorted(grid, time, side="right") - 1
    if position < 0:
        return float(values[0])
    return float(values[min(position, len(values) - 1)])


def finegray_transform_truncated(
    frame: pd.DataFrame,
    entry_col: str,
    exit_col: str,
    event_col: str,
    cause: int,
    id_col: str = "seqn",
) -> pd.DataFrame:
    columns = [id_col, entry_col, exit_col, event_col]
    data = frame[columns].dropna().copy()
    data[entry_col] = data[entry_col].astype(float)
    data[exit_col] = data[exit_col].astype(float)
    data = data[data[exit_col] > data[entry_col]]

    entry = data[entry_col].to_numpy()
    exit_time = data[exit_col].to_numpy()
    events = data[event_col].to_numpy()
    identifiers = data[id_col].to_numpy()

    grid, observed = observation_probability(entry, exit_time, events == 0)
    cause_times = np.unique(exit_time[events == cause])

    rows = []
    for start, stop, event, identifier in zip(
        entry, exit_time, events, identifiers, strict=False
    ):
        if event == cause:
            rows.append((identifier, start, stop, 1, 1.0))
```

```
            continue
        rows.append((identifier, start, stop, 0, 1.0))
        if event == 0:
            continue
        observed_at_exit = _lookup(grid, observed, stop)
        if observed_at_exit <= 0:
            continue
        previous = stop
        for later in cause_times[cause_times > stop]:
            weight = _lookup(grid, observed, later) / observed_at_exit
            if weight <= 1e-6:
                break
            rows.append((identifier, previous, later, 0, weight))
            previous = later

    long_frame = pd.DataFrame(
        rows, columns=[id_col, "tstart", "tstop", "fgstatus", "fgwt"]
    )
    long_frame = long_frame[long_frame["tstart"] < long_frame["tstop"]]
    return long_frame.reset_index(drop=True)


def _encode_features(
    frame: pd.DataFrame, features: list[str], id_col: str
) -> pd.DataFrame:
    selected = frame.set_index(id_col)[features]
    categorical = [
        name
        for name in features
        if selected[name].dtype == "object"
        or str(selected[name].dtype) == "boolean"
    ]
    encoded = pd.get_dummies(selected, columns=categorical, drop_first=True)
    return encoded.apply(pd.to_numeric, errors="coerce")


def fit_finegray_truncated(
    panel: pd.DataFrame,
    features: list[str],
    entry_col: str,
    exit_col: str,
    event_col: str,
    cause: int,
    design: survey.Design,
    id_col: str = "seqn",
    penalizer: float = 0.5,
) -> tuple[CoxTimeVaryingFitter, pd.DataFrame]:
    needed = features + [entry_col, exit_col, event_col, id_col]
    complete = panel.dropna(subset=needed).copy()
    long_frame = finegray_transform_truncated(
        complete, entry_col, exit_col, event_col, cause, id_col=id_col
    )

    weights = pd.Series(
        design.weight.to_numpy(),
        index=panel.loc[design.weight.index, id_col].to_numpy(),
    )
    long_frame["design_w"] = long_frame[id_col].map(weights)
    long_frame = long_frame.dropna(subset=["design_w"])
    long_frame["row_weight"] = long_frame["fgwt"] * long_frame["design_w"]

    encoded = _encode_features(complete, features, id_col)
    long_frame = long_frame.join(encoded, on=id_col)
    long_frame = long_frame.dropna(subset=list(encoded.columns))

    model_columns = [
        id_col,
        "tstart",
        "tstop",
        "fgstatus",
        "row_weight",
    ] + list(encoded.columns)
    fitter = CoxTimeVaryingFitter(penalizer=penalizer)
    fitter.fit(
        long_frame[model_columns],
        event_col="fgstatus",
```

```python
        start_col="tstart",
        stop_col="tstop",
        weights_col="row_weight",
        id_col=id_col,
        robust=False,
        show_progress=False,
    )
    return fitter, long_frame


def design_ci_truncated(
    panel: pd.DataFrame,
    features: list[str],
    entry_col: str,
    exit_col: str,
    event_col: str,
    cause: int,
    design: survey.Design,
    coefficients: list[str],
    id_col: str = "seqn",
    max_units: int | None = None,
    penalizer: float = 0.5,
) -> pd.DataFrame:
    needed = features + [entry_col, exit_col, event_col, id_col]
    complete = panel.dropna(subset=needed).copy()
    long_frame = finegray_transform_truncated(
        complete, entry_col, exit_col, event_col, cause, id_col=id_col
    )
    encoded = _encode_features(complete, features, id_col)
    long_frame = long_frame.join(encoded, on=id_col)
    long_frame = long_frame.dropna(subset=list(encoded.columns)).reset_index(
        drop=True
    )
    feature_columns = list(encoded.columns)

    identifiers = long_frame[id_col].to_numpy()
    tstart = long_frame["tstart"].to_numpy(dtype="float32")
    tstop = long_frame["tstop"].to_numpy(dtype="float32")
    status = long_frame["fgstatus"].to_numpy(dtype="int8")
    transform_weight = long_frame["fgwt"].to_numpy(dtype="float32")
    feature_matrix = long_frame[feature_columns].to_numpy(dtype="float32")
    del long_frame
    gc.collect()

    order = complete.loc[design.weight.index, id_col].to_numpy()
    position_of = {identifier: index for index, identifier in enumerate(order)}
    positions = np.array(
        [position_of.get(identifier, -1) for identifier in identifiers]
    )
    known = positions >= 0
    positions_safe = np.clip(positions, 0, None)

    def fit_once(weight_vector: np.ndarray) -> dict[str, float]:
        design_weight = np.where(known, weight_vector[positions_safe], 0.0)
        keep = known & (design_weight > 0)
        data = {
            id_col: identifiers[keep],
            "tstart": tstart[keep],
            "tstop": tstop[keep],
            "fgstatus": status[keep],
            "row_weight": (
                transform_weight[keep] * design_weight[keep]
            ).astype("float32"),
        }
        for index, name in enumerate(feature_columns):
            data[name] = feature_matrix[keep, index]
        fitter = CoxTimeVaryingFitter(penalizer=penalizer)
        fitter.fit(
            pd.DataFrame(data),
            event_col="fgstatus",
            start_col="tstart",
            stop_col="tstop",
            weights_col="row_weight",
            id_col=id_col,
            robust=False,
            show_progress=False,
```

```
        )
        summary = fitter.summary
        return {
            name: (
                float(summary.loc[name, "coef"])
                if name in summary.index
                else float("nan")
            )
            for name in coefficients
        }

    def fit_guarded(weight_vector: np.ndarray) -> dict[str, float]:
        for _attempt in range(3):
            try:
                return fit_once(weight_vector)
            except MemoryError:
                gc.collect()
                continue
            except Exception:
                return {name: float("nan") for name in coefficients}
        return {name: float("nan") for name in coefficients}

    point_estimate = fit_guarded(design.weight.to_numpy())
    replicates, units = survey.jackknife_replicates(design)
    replicates, units, scale = survey._subset_strata(
        replicates, units, max_units
    )

    replicate_estimates = []
    for index, replicate in enumerate(replicates):
        replicate_estimates.append(fit_guarded(replicate))
        if index % 5 == 0:
            gc.collect()

    rows = []
    for name in coefficients:
        values = np.array(
            [estimate[name] for estimate in replicate_estimates], dtype=float
        )
        finite = np.isfinite(values)
        kept_units = [
            unit for unit, good in zip(units, finite, strict=False) if good
        ]
        by_stratum: dict = {}
        for value, (stratum, _psu) in zip(
            values[finite], kept_units, strict=False
        ):
            by_stratum.setdefault(stratum, []).append(value)
        variance = 0.0
        unit_count = 0
        for stratum_values in by_stratum.values():
            size = len(stratum_values)
            if size < 2:
                continue
            array = np.asarray(stratum_values, dtype=float)
            variance += (
                (size - 1) / size * float(np.sum((array - array.mean()) ** 2))
            )
            unit_count += size
        degrees = max(unit_count - len(by_stratum), 1)
        point = point_estimate[name]
        if finite.sum() < 2 or not np.isfinite(point):
            rows.append(
                {
                    "feature": name,
                    "coef": point,
                    "coef_lo": float("nan"),
                    "coef_hi": float("nan"),
                    "n_jackknife_units": int(finite.sum()),
                    "n_failed_replicates": int((~finite).sum()),
                    "n_strata": len(by_stratum),
                    "variance_scale": scale,
                    "df": degrees,
                }
            )
            continue
```

```
        half_width = float(_st.t.ppf(0.975, degrees)) * float(
            np.sqrt(variance * scale)
        )
        rows.append(
            {
                "feature": name,
                "coef": point,
                "coef_lo": point - half_width,
                "coef_hi": point + half_width,
                "n_jackknife_units": int(finite.sum()),
                "n_failed_replicates": int((~finite).sum()),
                "n_strata": len(by_stratum),
                "variance_scale": scale,
                "df": degrees,
            }
        )
    table = pd.DataFrame(rows)
    table["HR"] = np.exp(table["coef"])
    table["HR_lo"] = np.exp(table["coef_lo"])
    table["HR_hi"] = np.exp(table["coef_hi"])
    return table
```

16 src/left_truncation.py

```
from __future__ import annotations

import logging

import numpy as np
import pandas as pd

from src import survey
from src.audits import MODEL_FEATURES
from src.cardiotoxicity import cardiotoxicity_group
from src.config import (
    DATA_PROCESSED,
    N_JACKKNIFE_MAX_UNITS,
    RESULTS_TABLES,
    configure_logging,
)
from src.finegray_truncated import (
    design_ci_truncated,
    finegray_transform_truncated,
    fit_finegray_truncated,
)

logger = logging.getLogger(__name__)

ENTRY = "time_since_diagnosis_years"
EXIT = "time_since_diagnosis_at_exit_years"
RECENT_DIAGNOSIS_YEARS = 5.0

DIAGNOSIS_SCALE_FEATURES = [name for name in MODEL_FEATURES if name != ENTRY]
HEADLINE = [
    "age",
    "sex_female",
    "hypertension",
    "diabetes",
    "crp",
    "bmi",
    "n_cancer_sites",
    "comorbidity_index",
    "smoking_status_never",
]


def main() -> None:
    configure_logging()
    panel = load_panel()
    eligible = diagnosis_scale_frame(panel)
    write_cohort_summary(panel, eligible)

    complete = eligible.dropna(
```

```
        subset=DIAGNOSIS_SCALE_FEATURES + [ENTRY, EXIT, "event"]
    )
    design = build_design(complete)
    logger.info(
        "delayed-entry cohort n=%d covariate-complete of %d eligible "
        "(design n=%d)",
        len(complete),
        len(eligible),
        design.n,
    )

    for cause, name in ((1, "cvd"), (2, "cancer")):
        fitter, _ = fit_finegray_truncated(
            complete,
            DIAGNOSIS_SCALE_FEATURES,
            ENTRY,
            EXIT,
            "event",
            cause=cause,
            design=design,
            penalizer=0.5,
        )
        table = design_ci_truncated(
            complete,
            DIAGNOSIS_SCALE_FEATURES,
            ENTRY,
            EXIT,
            "event",
            cause=cause,
            design=design,
            coefficients=available(fitter.summary.index, HEADLINE),
            max_units=N_JACKKNIFE_MAX_UNITS,
            penalizer=0.5,
        )
        path = (
            RESULTS_TABLES
            / f"finegray_{name}_death_left_truncated_design_ci.csv"
        )
        table.to_csv(path, index=False)
        logger.info(
            "%s death, diagnosis time scale:\n%s", name, table.to_string()
        )

    cardiotoxicity_contrast(eligible)
    recent_diagnosis_subset(eligible)
    logger.info("left-truncation analyses written")


def load_panel() -> pd.DataFrame:
    panel = pd.read_parquet(DATA_PROCESSED / "feature_panel_final.parquet")
    if "cardiotox_group" not in panel.columns:
        panel["cardiotox_group"] = cardiotoxicity_group(panel)
    return panel


def diagnosis_scale_frame(panel: pd.DataFrame) -> pd.DataFrame:
    frame = panel.copy()
    frame[EXIT] = frame[ENTRY] + frame["followup_years"]
    keep = frame[ENTRY].notna() & frame["followup_years"].notna()
    keep &= frame[EXIT] > frame[ENTRY]
    return frame[keep].copy()


def build_design(frame: pd.DataFrame) -> survey.Design:
    renamed = frame.rename(
        columns={
            "psu": "SDMVPSU",
            "strata": "SDMVSTRA",
            "weight_mec": "WTMEC2YR",
        }
    )
    return survey.build(renamed, "mec", n_cycles=10)


def write_cohort_summary(panel: pd.DataFrame, eligible: pd.DataFrame) -> None:
    complete = eligible.dropna(subset=DIAGNOSIS_SCALE_FEATURES + ["event"])
```

```
    recent = complete[complete[ENTRY] <= RECENT_DIAGNOSIS_YEARS]
    very_recent = complete[complete[ENTRY] <= 2.0]
    transform = finegray_transform_truncated(
        complete, ENTRY, EXIT, "event", cause=1
    )
    rows = [
        summary_row("survivor cohort", panel),
        summary_row("with a computable time since diagnosis", eligible),
        summary_row("covariate-complete, delayed entry", complete),
        summary_row(
            f"examined within {RECENT_DIAGNOSIS_YEARS:.0f} years of diagnosis",
            recent,
        ),
        summary_row("examined within 2 years of diagnosis", very_recent),
    ]
    table = pd.DataFrame(rows)
    table.to_csv(
        RESULTS_TABLES / "left_truncation_cohort_summary.csv", index=False
    )

    entry_quantiles = complete[ENTRY].quantile([0.25, 0.5, 0.75])
    scale_rows = [
        {
            "quantity": "entry time (years since diagnosis)",
            "q25": round(float(entry_quantiles.loc[0.25]), 1),
            "median": round(float(entry_quantiles.loc[0.5]), 1),
            "q75": round(float(entry_quantiles.loc[0.75]), 1),
            "maximum": round(float(complete[ENTRY].max()), 1),
        },
        {
            "quantity": "exit time (years since diagnosis)",
            "q25": round(float(complete[EXIT].quantile(0.25)), 1),
            "median": round(float(complete[EXIT].median()), 1),
            "q75": round(float(complete[EXIT].quantile(0.75)), 1),
            "maximum": round(float(complete[EXIT].max()), 1),
        },
        {
            "quantity": "inverse-probability weight in the risk-set extension",
            "q25": round(float(transform["fgwt"].quantile(0.25)), 3),
            "median": round(float(transform["fgwt"].median()), 3),
            "q75": round(float(transform["fgwt"].quantile(0.75)), 3),
            "maximum": round(float(transform["fgwt"].max()), 3),
        },
    ]
    pd.DataFrame(scale_rows).to_csv(
        RESULTS_TABLES / "left_truncation_time_scale.csv", index=False
    )
    logger.info(
        "delayed-entry time scale:\n%s", pd.DataFrame(scale_rows).to_string()
    )


def summary_row(label: str, frame: pd.DataFrame) -> dict:
    events = frame["event"]
    return {
        "subset": label,
        "n": int(len(frame)),
        "cardiovascular_deaths": int((events == 1).sum()),
        "cancer_deaths": int((events == 2).sum()),
        "other_deaths": int((events == 3).sum()),
        "median_years_since_diagnosis": (
            round(float(frame[ENTRY].median()), 1)
            if ENTRY in frame and frame[ENTRY].notna().any()
            else np.nan
        ),
    }


def cardiotoxicity_contrast(eligible: pd.DataFrame) -> None:
    features = [
        name for name in DIAGNOSIS_SCALE_FEATURES if name != "n_cancer_sites"
    ] + ["cardiotox_high"]
    grouped = eligible[
        eligible["cardiotox_group"].isin(
            ["High expected cardiotoxicity", "Low expected cardiotoxicity"]
        )
```

```
    ].copy()
    grouped["cardiotox_high"] = (
        grouped["cardiotox_group"] == "High expected cardiotoxicity"
    )
    grouped = grouped.dropna(subset=features + [ENTRY, EXIT, "event"])
    design = build_design(grouped)
    logger.info(
        "cardiotoxicity contrast under delayed entry: n=%d (high=%d, low=%d)",
        len(grouped),
        int(grouped["cardiotox_high"].sum()),
        int((~grouped["cardiotox_high"]).sum()),
    )

    fitter, _ = fit_finegray_truncated(
        grouped,
        features,
        ENTRY,
        EXIT,
        "event",
        cause=1,
        design=design,
        penalizer=0.5,
    )
    names = [name for name in fitter.summary.index if "cardiotox" in name]
    table = design_ci_truncated(
        grouped,
        features,
        ENTRY,
        EXIT,
        "event",
        cause=1,
        design=design,
        coefficients=names,
        max_units=N_JACKKNIFE_MAX_UNITS,
        penalizer=0.5,
    )
    table.to_csv(
        RESULTS_TABLES
        / "cardiotoxicity_cvd_death_left_truncated_design_ci.csv",
        index=False,
    )
    logger.info(
        "cardiotoxicity contrast, diagnosis time scale:\n%s", table.to_string()
    )

    recent = grouped[grouped[ENTRY] <= RECENT_DIAGNOSIS_YEARS].copy()
    if int((recent["event"] == 1).sum()) >= 20:
        design_recent = build_design(recent)
        recent_table = design_ci_truncated(
            recent,
            features,
            ENTRY,
            EXIT,
            "event",
            cause=1,
            design=design_recent,
            coefficients=names,
            max_units=N_JACKKNIFE_MAX_UNITS,
            penalizer=0.5,
        )
        recent_table["n"] = len(recent)
        recent_table["cardiovascular_deaths"] = int(
            (recent["event"] == 1).sum()
        )
        recent_table.to_csv(
            RESULTS_TABLES
            / "cardiotoxicity_cvd_death_recent_diagnosis_design_ci.csv",
            index=False,
        )
        logger.info(
            "cardiotoxicity contrast, examined within %.0f years:\n%s",
            RECENT_DIAGNOSIS_YEARS,
            recent_table.to_string(),
        )
    else:
        logger.info(
```

```
            "cardiotoxicity contrast within %.0f years not estimable: "
            "%d cardiovascular deaths",
            RECENT_DIAGNOSIS_YEARS,
            int((recent["event"] == 1).sum()),
        )


def recent_diagnosis_subset(eligible: pd.DataFrame) -> None:
    recent = eligible[eligible[ENTRY] <= RECENT_DIAGNOSIS_YEARS].copy()
    recent = recent.dropna(
        subset=DIAGNOSIS_SCALE_FEATURES + [ENTRY, EXIT, "event"]
    )
    design = build_design(recent)
    logger.info(
        "examined within %.0f years of diagnosis: n=%d (%d cardiovascular, "
        "%d cancer deaths)",
        RECENT_DIAGNOSIS_YEARS,
        len(recent),
        int((recent["event"] == 1).sum()),
        int((recent["event"] == 2).sum()),
    )
    for cause, name in ((1, "cvd"), (2, "cancer")):
        if int((recent["event"] == cause).sum()) < 20:
            logger.info(
                "%s death not estimable in the recent-diagnosis subset", name
            )
            continue
        fitter, _ = fit_finegray_truncated(
            recent,
            DIAGNOSIS_SCALE_FEATURES,
            ENTRY,
            EXIT,
            "event",
            cause=cause,
            design=design,
            penalizer=0.5,
        )
        table = design_ci_truncated(
            recent,
            DIAGNOSIS_SCALE_FEATURES,
            ENTRY,
            EXIT,
            "event",
            cause=cause,
            design=design,
            coefficients=available(fitter.summary.index, HEADLINE),
            max_units=N_JACKKNIFE_MAX_UNITS,
            penalizer=0.5,
        )
        table["n"] = len(recent)
        table.to_csv(
            RESULTS_TABLES
            / f"finegray_{name}_death_recent_diagnosis_design_ci.csv",
            index=False,
        )
        logger.info(
            "%s death, examined within %.0f years:\n%s",
            name,
            RECENT_DIAGNOSIS_YEARS,
            table.to_string(),
        )


def available(fitted_names, desired: list[str]) -> list[str]:
    present = [name for name in desired if name in fitted_names]
    if not any(name.startswith("smoking_status") for name in present):
        smoking = [
            name
            for name in fitted_names
            if str(name).startswith("smoking_status")
        ]
        present += smoking[:1]
    return present


if __name__ == "__main__":
```

```python
    main()
```

17 src/negative_control.py

```python
from __future__ import annotations

import gc
import logging

import numpy as np
import pandas as pd
from lifelines import CoxPHFitter

from src import survey

from .cancer_items import NON_MELANOMA_SKIN_GROUP, cancer_events_long
from .config import (
    DATA_PROCESSED,
    LEADING_CAUSE_DETAILED_CYCLES,
    configure_logging,
)
from .covariates import build_features
from .lmf_loader import load_lmf
from .nhanes_loader import attach_covariates, base_population, pool_component

logger = logging.getLogger(__name__)

NC_CYCLES = LEADING_CAUSE_DETAILED_CYCLES

CONTROL_FEATURES_MINIMAL = ["age", "sex_female"]
CONTROL_FEATURES_FULL = [
    "age",
    "sex_female",
    "race_eth",
    "education",
    "pir",
    "bmi",
    "smoking_status",
    "alcohol_ever_heavy",
    "physically_active",
    "hypertension",
    "diabetes",
    "comorbidity_index",
    "healthcare_visits_12mo",
    "general_health_rating",
]

EXPOSURE_COLUMNS = {
    "survivor": "cancer_survivor",
    "skin_only": "skin_cancer_only",
}


def build_negative_control_frame(cache: bool = True) -> pd.DataFrame:
    cache_path = DATA_PROCESSED / "control_population_panel.parquet"
    if cache and cache_path.exists():
        return pd.read_parquet(cache_path)

    merged = base_population()
    merged["RIDAGEYR"] = pd.to_numeric(merged["RIDAGEYR"], errors="coerce")
    adults = merged[merged["RIDAGEYR"] >= 20].copy()
    examined = adults[
        pd.to_numeric(adults["WTMEC2YR"], errors="coerce") > 0
    ].copy()
    examined = examined.rename(columns={"SEQN": "seqn"})
    examined["seqn"] = examined["seqn"].astype(int)

    reported = pd.to_numeric(examined["MCQ220"], errors="coerce")
    examined["reported_cancer"] = reported.map({1: True, 2: False})

    events = cancer_events_long(pool_component("MCQ"))
    site_counts = events.groupby("seqn").size().rename("n_cancer_sites")
    non_skin = (
```

```
        events.groupby("seqn")["site_group"]
        .apply(lambda group: bool((group != NON_MELANOMA_SKIN_GROUP).any()))
        .rename("has_non_skin_cancer")
    )
    examined = examined.merge(site_counts, on="seqn", how="left")
    examined = examined.merge(non_skin, on="seqn", how="left")

    has_site = examined["has_non_skin_cancer"].notna()
    skin_only = (
        examined["reported_cancer"].fillna(False)
        & has_site
        & ~examined["has_non_skin_cancer"].astype("boolean").fillna(False)
    )
    examined["skin_cancer_only"] = skin_only
    examined["cancer_survivor"] = (
        examined["reported_cancer"].fillna(False) & ~skin_only
    )
    examined["cancer_group"] = np.where(
        examined["reported_cancer"].isna(),
        "unknown",
        np.where(
            skin_only,
            "skin_only",
            np.where(examined["cancer_survivor"], "survivor", "none"),
        ),
    )

    for column in (
        "site_group",
        "site_name",
        "age_at_diagnosis",
        "time_since_diagnosis_years",
    ):
        examined[column] = pd.NA

    with_covariates = attach_covariates(examined)
    panel = build_features(
        with_covariates, out_name="control_population_features.parquet"
    )
    panel["cancer_group"] = with_covariates["cancer_group"].to_numpy()
    panel["cancer_survivor"] = (
        with_covariates["cancer_survivor"].astype(bool).to_numpy()
    )
    panel["skin_cancer_only"] = (
        with_covariates["skin_cancer_only"]
        .astype("boolean")
        .fillna(False)
        .astype(bool)
        .to_numpy()
    )

    mortality = load_lmf()[
        [
            "SEQN",
            "MORTSTAT",
            "UCOD_LEADING",
            "cv_death",
            "cancer_death",
            "followup_months",
        ]
    ]
    mortality = mortality.rename(
        columns={"SEQN": "seqn", "cancer_death": "cancer_death_lmf"}
    )
    panel = panel.drop(
        columns=[
            column
            for column in (
                "event",
                "cvd_death",
                "cancer_death",
                "other_death",
                "any_death",
                "followup_years",
                "MORTSTAT",
            )
```

```python
            if column in panel.columns
        ]
    )
    panel = panel.merge(mortality, on="seqn", how="left")
    panel["followup_years"] = (
        pd.to_numeric(panel["followup_months"], errors="coerce") / 12.0
    )
    code = panel["UCOD_LEADING"].astype(str).str.strip().str.zfill(3)
    dead = panel["MORTSTAT"] == 1
    panel["accidental_death"] = (dead & (code == "004")).astype("int8")
    panel["cardiovascular_death"] = (
        dead & panel["cv_death"].astype("boolean").fillna(False)
    ).astype("int8")
    panel["cancer_death"] = (
        dead & panel["cancer_death_lmf"].astype("boolean").fillna(False)
    ).astype("int8")
    panel["detailed_cause_cycle"] = panel["cycle"].isin(NC_CYCLES)

    panel.to_parquet(cache_path, index=False)
    return panel


def main() -> None:
    configure_logging()
    panel = build_negative_control_frame()
    logger.info(
        "control population n=%d (%s)",
        len(panel),
        panel["cancer_group"].value_counts().to_dict(),
    )


def negative_control_test(
    frame: pd.DataFrame,
    features: list[str] | None = None,
    max_units: int | None = None,
    n_cycles: int = len(NC_CYCLES),
) -> dict:
    features = CONTROL_FEATURES_FULL if features is None else features
    table = contrast_table(
        frame,
        "accidental_death",
        features,
        restrict_to_detailed_cycles=True,
        max_units=max_units,
        n_cycles=n_cycles,
    )
    row = table[table["contrast"] == "survivor_vs_no_cancer"].iloc[0]
    return {
        "outcome": "accidental_death (negative control)",
        "adjustment": (
            "full covariate set"
            if features is CONTROL_FEATURES_FULL
            else ", ".join(features)
        ),
        "coef": float(row["coef"]),
        "coef_lo": float(row["coef_lo"]),
        "coef_hi": float(row["coef_hi"]),
        "HR": float(row["HR"]),
        "HR_lo": float(row["HR_lo"]),
        "HR_hi": float(row["HR_hi"]),
        "n_total": int(row["n_analysed"]),
        "n_cancer_survivors": int(row["n_survivors"]),
        "n_skin_cancer_only": int(row["n_skin_only"]),
        "n_accidental_deaths": int(row["n_events"]),
    }


def contrast_table(
    frame: pd.DataFrame,
    outcome: str,
    features: list[str],
    restrict_to_detailed_cycles: bool,
    max_units: int | None,
    n_cycles: int = 10,
) -> pd.DataFrame:
```

```python
    data = frame[frame["cancer_group"] != "unknown"].copy()
    if restrict_to_detailed_cycles:
        data = data[data["detailed_cause_cycle"]]
    needed = features + [
        outcome,
        "followup_years",
        "psu",
        "strata",
        "weight_mec",
    ]
    data = data.dropna(subset=needed)
    data = data[data["followup_years"] > 0]

    design = survey.build(
        data.rename(
            columns={
                "psu": "SDMVPSU",
                "strata": "SDMVSTRA",
                "weight_mec": "WTMEC2YR",
            }
        ),
        "mec",
        n_cycles=n_cycles,
    )
    data = data.loc[design.weight.index]

    model_frame = encode_control_features(data, features)
    model_frame["survivor"] = data["cancer_survivor"].astype(float).to_numpy()
    model_frame["skin_only"] = (
        data["skin_cancer_only"].astype(float).to_numpy()
    )
    model_frame["duration"] = data["followup_years"].astype(float).to_numpy()
    model_frame["event"] = data[outcome].astype(int).to_numpy()

    def statistics(weights: np.ndarray) -> dict[str, float]:
        for _attempt in range(3):
            try:
                return fit_contrasts(model_frame, weights)
            except MemoryError:
                gc.collect()
                continue
            except Exception:
                return {
                    "survivor": float("nan"),
                    "skin_only": float("nan"),
                    "calibrated": float("nan"),
                }
        return {
            "survivor": float("nan"),
            "skin_only": float("nan"),
            "calibrated": float("nan"),
        }

    estimates = survey.design_jackknife_ci_multi(
        statistics, design, max_units=max_units
    )

    labels = {
        "survivor": "survivor_vs_no_cancer",
        "skin_only": "skin_cancer_only_vs_no_cancer",
        "calibrated": "survivor_minus_skin_cancer_only",
    }
    rows = []
    for key, label in labels.items():
        point, low, high = estimates[key]
        rows.append(
            {
                "outcome": outcome,
                "contrast": label,
                "adjustment": ", ".join(features),
                "coef": point,
                "coef_lo": low,
                "coef_hi": high,
                "HR": float(np.exp(point)),
                "HR_lo": float(np.exp(low)) if np.isfinite(low) else np.nan,
                "HR_hi": float(np.exp(high)) if np.isfinite(high) else np.nan,
```

```
                "n_analysed": int(len(data)),
                "n_survivors": int(data["cancer_survivor"].sum()),
                "n_skin_only": int(data["skin_cancer_only"].sum()),
                "n_events": int(data[outcome].sum()),
                "n_events_survivors": int(
                    data.loc[data["cancer_survivor"], outcome].sum()
                ),
                "n_events_skin_only": int(
                    data.loc[data["skin_cancer_only"], outcome].sum()
                ),
                "n_cycles": int(data["cycle"].nunique()),
            }
        )
    return pd.DataFrame(rows)


def encode_control_features(
    data: pd.DataFrame, features: list[str]
) -> pd.DataFrame:
    selected = data[features].copy()
    categorical = [
        name
        for name in features
        if selected[name].dtype == "object"
        or str(selected[name].dtype) == "boolean"
    ]
    encoded = pd.get_dummies(selected, columns=categorical, drop_first=True)
    encoded = encoded.apply(pd.to_numeric, errors="coerce")
    return encoded.reset_index(drop=True)


def fit_contrasts(
    model_frame: pd.DataFrame, weights: np.ndarray
) -> dict[str, float]:
    frame = model_frame.copy()
    frame["weight"] = weights
    frame = frame[frame["weight"] > 0]
    fitter = CoxPHFitter(penalizer=0.1)
    fitter.fit(
        frame,
        duration_col="duration",
        event_col="event",
        weights_col="weight",
        robust=False,
        show_progress=False,
    )
    survivor = float(fitter.summary.loc["survivor", "coef"])
    skin_only = float(fitter.summary.loc["skin_only", "coef"])
    return {
        "survivor": survivor,
        "skin_only": skin_only,
        "calibrated": survivor - skin_only,
    }


if __name__ == "__main__":
    main()
```

18 src/relative_survival.py

```
from __future__ import annotations

import logging

import numpy as np
import pandas as pd
from lifelines import CoxTimeVaryingFitter

from src import survey
from src.config import DATA_PROCESSED, RESULTS_TABLES, configure_logging
from src.negative_control import build_negative_control_frame

logger = logging.getLogger(__name__)
```

```python

HORIZONS = (5.0, 10.0)

DEMOGRAPHIC_FEATURES = [
    "sex_female",
    "race_eth",
    "education",
    "pir",
    "smoking_status",
    "period_year",
]
EXTENDED_FEATURES = DEMOGRAPHIC_FEATURES + ["bmi", "hypertension", "diabetes"]


def main() -> None:
    configure_logging()
    control = build_negative_control_frame()
    control["period_year"] = control["cycle"].str.slice(0, 4).astype(float)

    survivors = load_survivors()
    rows = []
    for features, label in (
        (DEMOGRAPHIC_FEATURES, "demographic"),
        (EXTENDED_FEATURES, "demographic and baseline clinical"),
    ):
        rows.extend(
            relative_survival_rows(control, survivors, features, label)
        )
    table = pd.DataFrame(rows)
    table.to_csv(
        RESULTS_TABLES / "relative_survival_estimates.csv", index=False
    )
    logger.info("relative survival estimates:\n%s", table.to_string())


def load_survivors() -> pd.DataFrame:
    panel = pd.read_parquet(DATA_PROCESSED / "feature_panel_final.parquet")
    panel["period_year"] = panel["cycle"].str.slice(0, 4).astype(float)
    panel["any_death"] = (panel["event"] > 0).astype(int)
    keep = panel["followup_years"].notna() & (panel["followup_years"] > 0)
    return panel[keep].copy()


def relative_survival_rows(
    control: pd.DataFrame,
    survivors: pd.DataFrame,
    features: list[str],
    label: str,
) -> list[dict]:
    reference = control[control["cancer_group"] == "none"].copy()
    reference["any_death"] = (reference["MORTSTAT"] == 1).astype(int)
    needed = features + [
        "age",
        "followup_years",
        "any_death",
        "psu",
        "strata",
        "weight_mec",
    ]
    reference = reference.dropna(subset=needed)
    reference = reference[reference["followup_years"] > 0]
    reference_design = build_design(reference)
    reference = reference.loc[reference_design.weight.index]

    coefficients, columns = fit_expected_model(
        reference, features, reference_design
    )
    logger.info(
        "expected-mortality model (%s): n=%d, %d deaths, %d coefficients",
        label,
        len(reference),
        int(reference["any_death"].sum()),
        len(coefficients),
    )

    grid, baseline = breslow_baseline(
```

```python
        reference, reference_design, coefficients, features, columns
    )

    cohort = survivors.dropna(subset=features + ["age"]).copy()
    cohort_design = build_design(cohort)
    cohort = cohort.loc[cohort_design.weight.index]
    linear = linear_predictor(cohort, features, columns, coefficients)
    logger.info(
        "survivor cohort for relative survival (%s): n=%d, %d deaths",
        label,
        len(cohort),
        int(cohort["any_death"].sum()),
    )

    expected = expected_curves(cohort, linear, grid, baseline)

    def statistics(weights: np.ndarray) -> dict[str, float]:
        return horizon_statistics(cohort, expected, weights)

    estimates = survey.design_jackknife_ci_multi(statistics, cohort_design)

    rows = []
    for horizon in HORIZONS:
        for quantity in (
            "observed_survival",
            "expected_survival",
            "relative_survival_ratio",
            "net_survival",
            "cancer_death_incidence",
        ):
            key = f"{quantity}_{horizon:.0f}y"
            point, low, high = estimates[key]
            rows.append(
                {
                    "expected_model": label,
                    "horizon_years": horizon,
                    "quantity": quantity,
                    "estimate": point,
                    "ci_low": low,
                    "ci_high": high,
                    "n_survivors": int(len(cohort)),
                    "n_survivor_deaths": int(cohort["any_death"].sum()),
                    "n_reference_adults": int(len(reference)),
                    "n_reference_deaths": int(reference["any_death"].sum()),
                }
            )
    return rows


def build_design(frame: pd.DataFrame) -> survey.Design:
    renamed = frame.rename(
        columns={
            "psu": "SDMVPSU",
            "strata": "SDMVSTRA",
            "weight_mec": "WTMEC2YR",
        }
    )
    return survey.build(renamed, "mec", n_cycles=10)


def design_matrix(frame: pd.DataFrame, features: list[str]) -> pd.DataFrame:
    selected = frame[features].copy()
    categorical = [
        name
        for name in features
        if selected[name].dtype == "object"
        or str(selected[name].dtype) == "boolean"
    ]
    encoded = pd.get_dummies(selected, columns=categorical, drop_first=True)
    return encoded.apply(pd.to_numeric, errors="coerce").astype(float)


def fit_expected_model(
    reference: pd.DataFrame, features: list[str], design: survey.Design
) -> tuple[pd.Series, list[str]]:
    matrix = design_matrix(reference, features)
```

```
    frame = matrix.reset_index(drop=True)
    frame["identifier"] = np.arange(len(frame))
    frame["entry_age"] = reference["age"].to_numpy(dtype=float)
    frame["exit_age"] = reference["age"].to_numpy(dtype=float) + reference[
        "followup_years"
    ].to_numpy(dtype=float)
    frame["event"] = reference["any_death"].to_numpy(dtype=int)
    frame["weight"] = design.weight.to_numpy()
    frame = frame[frame["exit_age"] > frame["entry_age"]]
    fitter = CoxTimeVaryingFitter(penalizer=0.01)
    fitter.fit(
        frame,
        id_col="identifier",
        start_col="entry_age",
        stop_col="exit_age",
        event_col="event",
        weights_col="weight",
        robust=False,
        show_progress=False,
    )
    return fitter.params_, list(matrix.columns)


def linear_predictor(
    frame: pd.DataFrame,
    features: list[str],
    columns: list[str],
    coefficients: pd.Series,
) -> np.ndarray:
    built = design_matrix(frame, features)
    for column in columns:
        if column not in built.columns:
            built[column] = 0.0
    built = built[columns]
    return (
        built.to_numpy(dtype=float) @ coefficients.reindex(columns).to_numpy()
    )


def breslow_baseline(
    reference: pd.DataFrame,
    design: survey.Design,
    coefficients: pd.Series,
    features: list[str],
    columns: list[str],
) -> tuple[np.ndarray, np.ndarray]:
    entry = reference["age"].to_numpy(dtype=float)
    exit_age = entry + reference["followup_years"].to_numpy(dtype=float)
    died = reference["any_death"].to_numpy(dtype=int) == 1
    weights = design.weight.to_numpy()
    risk = np.exp(linear_predictor(reference, features, columns, coefficients))

    grid = np.unique(exit_age[died])
    increments = np.zeros(len(grid), dtype=float)
    for index, age in enumerate(grid):
        at_risk = (entry < age) & (exit_age >= age)
        denominator = float(np.sum(weights[at_risk] * risk[at_risk]))
        if denominator <= 0:
            continue
        numerator = float(np.sum(weights[died & (exit_age == age)]))
        increments[index] = numerator / denominator
    return grid, increments


def expected_curves(
    cohort: pd.DataFrame,
    linear: np.ndarray,
    grid: np.ndarray,
    baseline: np.ndarray,
) -> dict[str, np.ndarray]:
    age = cohort["age"].to_numpy(dtype=float)
    follow_up = cohort["followup_years"].to_numpy(dtype=float)
    relative_risk = np.exp(linear)
    cumulative = np.concatenate([[0.0], np.cumsum(baseline)])

    def cumulative_at(ages: np.ndarray) -> np.ndarray:
```

```python
        positions = np.searchsorted(grid, ages, side="right")
        return cumulative[positions]

    event_times = np.unique(follow_up[cohort["any_death"].to_numpy() == 1])
    times = np.unique(np.concatenate([event_times, np.array(HORIZONS)]))
    times = times[times > 0]

    survival = np.empty((len(cohort), len(times)), dtype=float)
    hazard = np.empty((len(cohort), len(times)), dtype=float)
    base_at_entry = cumulative_at(age)
    previous = np.zeros(len(cohort), dtype=float)
    previous_time = 0.0
    for index, time_point in enumerate(times):
        cumulative_hazard = (
            cumulative_at(age + time_point) - base_at_entry
        ) * relative_risk
        survival[:, index] = np.exp(-cumulative_hazard)
        step = time_point - previous_time
        hazard[:, index] = np.where(
            step > 0, (cumulative_hazard - previous) / step, 0.0
        )
        previous = cumulative_hazard
        previous_time = time_point
    return {"times": times, "survival": survival, "hazard": hazard}


def horizon_statistics(
    cohort: pd.DataFrame, expected: dict[str, np.ndarray], weights: np.ndarray
) -> dict[str, float]:
    times = expected["times"]
    survival = expected["survival"]
    hazard = expected["hazard"]
    follow_up = cohort["followup_years"].to_numpy(dtype=float)
    event = cohort["event"].to_numpy(dtype=int)
    died = (event > 0).astype(int)

    output: dict[str, float] = {}
    positive = weights > 0
    weight = np.where(positive, weights, 0.0)

    observed_hazard = np.zeros(len(times), dtype=float)
    net_hazard = np.zeros(len(times), dtype=float)
    cancer_hazard = np.zeros(len(times), dtype=float)
    all_cause_hazard = np.zeros(len(times), dtype=float)
    for index, time_point in enumerate(times):
        at_risk = follow_up >= time_point
        pohar_weight = weight / np.maximum(survival[:, index], 1e-12)
        denominator_pp = float(np.sum(pohar_weight * at_risk))
        denominator = float(np.sum(weight * at_risk))
        died_now = at_risk & (follow_up == time_point) & (died == 1)
        cancer_now = at_risk & (follow_up == time_point) & (event == 2)
        if denominator > 0:
            all_cause_hazard[index] = (
                float(np.sum(weight * died_now)) / denominator
            )
            cancer_hazard[index] = (
                float(np.sum(weight * cancer_now)) / denominator
            )
        if denominator_pp > 0:
            observed_hazard[index] = (
                float(np.sum(pohar_weight * died_now)) / denominator_pp
            )
            expected_part = float(
                np.sum(pohar_weight * at_risk * hazard[:, index])
            )
            step = time_point - (times[index - 1] if index else 0.0)
            net_hazard[index] = (
                observed_hazard[index] - expected_part / denominator_pp * step
            )

    overall_survival = np.cumprod(1.0 - all_cause_hazard)
    net_survival = np.exp(-np.cumsum(net_hazard))
    incidence = np.cumsum(
        cancer_hazard * np.concatenate([[1.0], overall_survival[:-1]])
    )
    for horizon in HORIZONS:
```

```python
        position = int(np.searchsorted(times, horizon, side="right") - 1)
        position = max(position, 0)
        expected_mean = float(
            np.sum(weight * survival[:, position])
            / max(float(np.sum(weight)), 1e-12)
        )
        observed = float(overall_survival[position])
        output[f"observed_survival_{horizon:.0f}y"] = observed
        output[f"expected_survival_{horizon:.0f}y"] = expected_mean
        output[f"relative_survival_ratio_{horizon:.0f}y"] = (
            observed / expected_mean if expected_mean > 0 else float("nan")
        )
        output[f"net_survival_{horizon:.0f}y"] = float(net_survival[position])
        output[f"cancer_death_incidence_{horizon:.0f}y"] = float(
            incidence[position]
        )
    return output


if __name__ == "__main__":
    main()
```

19 src/gbm_competing_risk.py

```python
from __future__ import annotations

import numpy as np
import pandas as pd
import xgboost as xgb
from sklearn.model_selection import KFold

from src import survey

from .config import RANDOM_SEED


def _encode(X: pd.DataFrame) -> pd.DataFrame:
    Xe = X.copy()
    for c in Xe.columns:
        if str(Xe[c].dtype) == "boolean":
            Xe[c] = Xe[c].astype("Int8").astype("float64")
        elif Xe[c].dtype == "object":
            Xe[c] = Xe[c].astype("category")
    return Xe


def _cox_label(followup_years: np.ndarray, is_event: np.ndarray) -> np.ndarray:
    return np.where(is_event, followup_years, -followup_years)


def fit_gbm_cause_specific(
    panel: pd.DataFrame,
    features: list[str],
    cause: int,
    n_folds: int = 5,
    seed: int = RANDOM_SEED,
) -> tuple[pd.Series, xgb.XGBRegressor]:
    d = panel.dropna(subset=features + ["followup_years", "event"]).copy()
    X = _encode(d[features])
    y = _cox_label(
        d["followup_years"].to_numpy(), (d["event"] == cause).to_numpy()
    )

    kf = KFold(n_folds, shuffle=True, random_state=seed)
    oof = pd.Series(np.nan, index=d.index)
    for tr, te in kf.split(X):
        model = xgb.XGBRegressor(
            objective="survival:cox",
            n_estimators=200,
            max_depth=3,
            learning_rate=0.05,
            subsample=0.8,
            colsample_bytree=0.8,
```

```
            enable_categorical=True,
            tree_method="hist",
            random_state=seed,
            n_jobs=-1,
            verbosity=0,
        )
        model.fit(X.iloc[tr], y[tr])
        oof.iloc[te] = model.predict(X.iloc[te])

    final_model = xgb.XGBRegressor(
        objective="survival:cox",
        n_estimators=200,
        max_depth=3,
        learning_rate=0.05,
        subsample=0.8,
        colsample_bytree=0.8,
        enable_categorical=True,
        tree_method="hist",
        random_state=seed,
        n_jobs=-1,
        verbosity=0,
    )
    final_model.fit(X, y)
    return oof, final_model


def weighted_concordance(
    time: np.ndarray,
    event: np.ndarray,
    risk: np.ndarray,
    weight: np.ndarray,
    cause: int,
) -> float:
    n = len(time)
    is_case = event == cause
    num = 0.0
    den = 0.0
    order = np.argsort(time)
    time_s, risk_s, w_s = time[order], risk[order], weight[order]
    is_case_s = is_case[order]
    for i in range(n):
        if not is_case_s[i]:
            continue
        later = time_s > time_s[i]
        if not later.any():
            continue
        w_pair = w_s[i] * w_s[later]
        concordant = risk_s[i] > risk_s[later]
        ties = risk_s[i] == risk_s[later]
        num += float(np.sum(w_pair * (concordant + 0.5 * ties)))
        den += float(np.sum(w_pair))
    return num / den if den > 0 else float("nan")


def design_ci_c_index(
    panel: pd.DataFrame,
    oof_risk: pd.Series,
    cause: int,
    design_frame_cols: dict,
    n_cycles: int,
    max_units: int = 60,
) -> dict:
    idx = oof_risk.dropna().index
    sub = panel.loc[idx]
    design_frame = pd.DataFrame(
        {
            "SDMVPSU": sub[design_frame_cols["psu"]].to_numpy(),
            "SDMVSTRA": sub[design_frame_cols["strata"]].to_numpy(),
            "WTMEC2YR": sub[design_frame_cols["weight"]].to_numpy(),
        },
        index=idx,
    )
    d = survey.build(design_frame, "mec", n_cycles=n_cycles)
    keep = d.weight.index
    time = sub.loc[keep, "followup_years"].to_numpy()
    event = sub.loc[keep, "event"].to_numpy()
```

```python
    risk = oof_risk.loc[keep].to_numpy()

    def statistic(w: np.ndarray) -> float:
        return weighted_concordance(time, event, risk, w, cause)

    point, lo, hi = survey.design_jackknife_ci(
        statistic, d, max_units=max_units
    )
    width = hi - lo if np.isfinite(hi) and np.isfinite(lo) else float("nan")
    suspect = np.isfinite(width) and width < 0.02

    _, all_units = survey.jackknife_replicates(d)
    strata_order = []
    for stratum, _psu in all_units:
        if stratum not in strata_order:
            strata_order.append(stratum)
    if max_units is not None and len(all_units) > max_units:
        sizes = {
            s: sum(1 for st, _ in all_units if st == s) for s in strata_order
        }
        mean_size = max(sum(sizes.values()) / len(sizes), 1.0)
        n_keep = max(int(max_units // mean_size), 2)
        used_strata = min(n_keep, len(strata_order))
        used_units = min(max_units, len(all_units))
    else:
        used_strata = len(strata_order)
        used_units = len(all_units)
    df_used = max(used_units - used_strata, 1)

    return {
        "c_index": point,
        "c_index_lo": lo,
        "c_index_hi": hi,
        "ci_width": width,
        "suspect_narrow_ci": bool(suspect),
        "n": len(keep),
        "n_jackknife_units_available": len(all_units),
        "n_jackknife_units_used": used_units,
        "n_strata_used": used_strata,
        "df": df_used,
    }
```

20 src/calibration_dca.py

```python
from __future__ import annotations

import numpy as np
import pandas as pd
from sklearn.linear_model import LogisticRegression
from sklearn.metrics import brier_score_loss


def pseudo_binary_outcome(
    time: np.ndarray, event: np.ndarray, cause: int, horizon: float = 10.0
) -> tuple[np.ndarray, np.ndarray]:
    had_cause_by_horizon = (event == cause) & (time <= horizon)
    survived_to_horizon_event_free = time >= horizon
    eligible = had_cause_by_horizon | survived_to_horizon_event_free
    outcome = had_cause_by_horizon.astype(int)
    return eligible, outcome


def calibrate_platt(
    risk_score: np.ndarray, outcome: np.ndarray
) -> tuple[np.ndarray, LogisticRegression]:
    lr = LogisticRegression(solver="lbfgs")
    lr.fit(risk_score.reshape(-1, 1), outcome)
    p = lr.predict_proba(risk_score.reshape(-1, 1))[:, 1]
    return p, lr


def calibration_table(
    p: np.ndarray, outcome: np.ndarray, n_bins: int = 10
```

```python
) -> pd.DataFrame:
    order = np.argsort(p)
    p_s, y_s = p[order], outcome[order]
    bins = np.array_split(np.arange(len(p_s)), n_bins)
    rows = []
    for i, b in enumerate(bins):
        if len(b) == 0:
            continue
        rows.append(
            {
                "decile": i + 1,
                "n": len(b),
                "mean_predicted": float(np.mean(p_s[b])),
                "observed_proportion": float(np.mean(y_s[b])),
            }
        )
    return pd.DataFrame(rows)


def brier_with_ci(
    p: np.ndarray, outcome: np.ndarray, n_boot: int = 1000, seed: int = 42
) -> dict:
    point = brier_score_loss(outcome, p)
    rng = np.random.default_rng(seed)
    n = len(p)
    boots = []
    for _ in range(n_boot):
        idx = rng.integers(0, n, n)
        boots.append(brier_score_loss(outcome[idx], p[idx]))
    lo, hi = np.quantile(boots, [0.025, 0.975])
    return {
        "brier": round(float(point), 3),
        "brier_lo": round(float(lo), 3),
        "brier_hi": round(float(hi), 3),
        "ci_method": "iid_bootstrap_NOT_design_based",
        "ci_note": (
            "Percentile bootstrap over independently resampled rows; "
            "ignores SDMVPSU/SDMVSTRA. Understates uncertainty relative "
            "to a design-based interval. Point estimate unaffected."
        ),
    }


def decision_curve(
    p: np.ndarray, outcome: np.ndarray, thresholds: np.ndarray | None = None
) -> pd.DataFrame:
    thresholds = (
        thresholds if thresholds is not None else np.linspace(0.01, 0.5, 50)
    )
    n = len(p)
    prevalence = outcome.mean()
    rows = []
    for pt in thresholds:
        predicted_pos = p >= pt
        tp = float(np.sum(predicted_pos & (outcome == 1)))
        fp = float(np.sum(predicted_pos & (outcome == 0)))
        nb_model = tp / n - fp / n * (pt / (1 - pt))
        nb_all = prevalence - (1 - prevalence) * (pt / (1 - pt))
        rows.append(
            {
                "threshold": round(float(pt), 3),
                "net_benefit_model": nb_model,
                "net_benefit_treat_all": nb_all,
                "net_benefit_treat_none": 0.0,
            }
        )
    return pd.DataFrame(rows)
```

21 src/shap_analysis.py

```
from __future__ import annotations

import re

import numpy as np
import pandas as pd
import shap
import shap.explainers._tree as _shap_tree_mod

from .config import RESULTS_METRICS, SHAP_MIN_STRATUM_N

_orig_decode_ubjson = _shap_tree_mod.decode_ubjson_buffer


def _patched_decode_ubjson(fd):
    jmodel = _orig_decode_ubjson(fd)
    try:
        lmp = jmodel["learner"]["learner_model_param"]
        bs = lmp.get("base_score")
        if isinstance(bs, str):
            m = re.match(r"\[?\s*([0-9.eE+-]+)\s*\]?", bs)
            if m:
                lmp["base_score"] = m.group(1)
    except Exception:
        pass
    return jmodel


_shap_tree_mod.decode_ubjson_buffer = _patched_decode_ubjson


def global_shap(model, X: pd.DataFrame, tag: str) -> pd.DataFrame:
    explainer = shap.TreeExplainer(model)
    sv = explainer.shap_values(X)
    if isinstance(sv, list):
        sv = sv[1]
    mean_abs = np.abs(sv).mean(axis=0)
    table = (
        pd.DataFrame({"feature": X.columns, "mean_abs_shap": mean_abs})
        .sort_values("mean_abs_shap", ascending=False)
        .reset_index(drop=True)
    )
    table["mean_abs_shap"] = table["mean_abs_shap"].round(3)
    table.to_csv(RESULTS_METRICS / f"shap_global_{tag}.csv", index=False)
    return table


def _filename_safe(label: str) -> str:
    return re.sub(r'[\\/:*?"<>|]', "-", str(label))


def stratified_shap(
    model, X: pd.DataFrame, strata: dict[str, pd.Series], tag: str
) -> tuple[dict[str, pd.DataFrame], pd.DataFrame]:
    explainer = shap.TreeExplainer(model)
    out: dict[str, pd.DataFrame] = {}
    n_rows = []
    for label, mask in strata.items():
        mask = mask.reindex(X.index).fillna(False).astype(bool)
        n = int(mask.sum())
        included = n >= SHAP_MIN_STRATUM_N
        n_rows.append({"stratum": label, "n": n, "included": included})
        if not included:
            continue
        sv = explainer.shap_values(X.loc[mask])
        if isinstance(sv, list):
            sv = sv[1]
        if sv.shape[0] != n:
            raise AssertionError(
                f"SHAP matrix rows ({sv.shape[0]}) != stratum n ({n}) "
                f"for {label!r}"
            )
        tbl = (
            pd.DataFrame(
```

```
                {
                    "feature": X.columns,
                    "mean_abs_shap": np.abs(sv).mean(axis=0),
                }
            )
            .sort_values("mean_abs_shap", ascending=False)
            .reset_index(drop=True)
        )
        tbl["mean_abs_shap"] = tbl["mean_abs_shap"].round(3)
        tbl.to_csv(
            RESULTS_METRICS / f"shap_{tag}_{_filename_safe(label)}.csv",
            index=False,
        )
        out[label] = tbl
    n_df = pd.DataFrame(n_rows)
    n_df.to_csv(RESULTS_METRICS / f"shap_strata_n_{tag}.csv", index=False)
    return out, n_df


def bootstrap_shap_rank_stability(
    model, X: pd.DataFrame, n_boot: int = 30, seed: int = 42
) -> float:
    from scipy.stats import spearmanr

    rng = np.random.default_rng(seed)
    explainer = shap.TreeExplainer(model)
    sv_full = explainer.shap_values(X)
    if isinstance(sv_full, list):
        sv_full = sv_full[1]
    full_rank = pd.Series(np.abs(sv_full).mean(axis=0), index=X.columns).rank(
        ascending=False
    )

    rhos = []
    n = len(X)
    for _ in range(n_boot):
        idx = rng.integers(0, n, n)
        sv = explainer.shap_values(X.iloc[idx])
        if isinstance(sv, list):
            sv = sv[1]
        rank = pd.Series(np.abs(sv).mean(axis=0), index=X.columns).rank(
            ascending=False
        )
        rho, _ = spearmanr(full_rank, rank)
        rhos.append(rho)
    return float(np.mean(rhos))
```

22 src/baseline_models.py

```
from __future__ import annotations

import argparse
import sys
import time
import warnings
from pathlib import Path

warnings.filterwarnings("ignore")

ROOT = Path(__file__).resolve().parents[1]

import numpy as np
import pandas as pd

from src import survey
from src.audits import MODEL_FEATURES
from src.config import RESULTS_METRICS, RESULTS_TABLES
from src.finegray import design_ci_for_coefficients, fit_finegray_cox, hr_table
from src.gbm_competing_risk import (
    design_ci_c_index,
    fit_gbm_cause_specific,
    weighted_concordance,
)
```

```python

T0 = time.time()
DESIGN_COLS = {"psu": "psu", "strata": "strata", "weight": "weight_mec"}
CAUSES = {1: "cvd_death", 2: "cancer_death"}


def log(msg: str) -> None:
    print(f"[{time.time() - T0:7.1f}s] {msg}", flush=True)


def load_panel() -> pd.DataFrame:
    p = pd.read_parquet(
        ROOT / "data" / "processed" / "feature_panel_final.parquet"
    )
    cvd5 = p["comorbidity_index"] - p["hypertension"] - p["diabetes"]
    if cvd5.min() < 0:
        raise AssertionError(
            "comorbidity decomposition produced a negative count"
        )
    if cvd5.max() > 5:
        raise AssertionError(
            "comorbidity decomposition exceeded 5 cardiovascular flags"
        )
    p["comorbidity_cvd5"] = cvd5.astype(float)
    log(
        f"panel n={len(p)}; cvd5 verified exact (range {cvd5.min():.0f}-{cvd5.max():.0f})"
    )
    return p


FEATURE_SETS = {
    "age_only": ["age"],
    "age_sex": ["age", "sex_female"],
    "full": MODEL_FEATURES,
}


def paired_delta_ci(panel, oof_a, oof_b, cause, max_units):
    idx = oof_a.dropna().index.intersection(oof_b.dropna().index)
    sub = panel.loc[idx]
    design_frame = pd.DataFrame(
        {
            "SDMVPSU": sub[DESIGN_COLS["psu"]].to_numpy(),
            "SDMVSTRA": sub[DESIGN_COLS["strata"]].to_numpy(),
            "WTMEC2YR": sub[DESIGN_COLS["weight"]].to_numpy(),
        },
        index=idx,
    )
    d = survey.build(design_frame, "mec", n_cycles=10)
    keep = d.weight.index
    time_v = sub.loc[keep, "followup_years"].to_numpy()
    event_v = sub.loc[keep, "event"].to_numpy()
    ra = oof_a.loc[keep].to_numpy()
    rb = oof_b.loc[keep].to_numpy()

    def statistic(w):
        return weighted_concordance(
            time_v, event_v, ra, w, cause
        ) - weighted_concordance(time_v, event_v, rb, w, cause)

    point, lo, hi = survey.design_jackknife_ci(
        statistic, d, max_units=max_units
    )
    return {"delta": point, "delta_lo": lo, "delta_hi": hi, "n": len(keep)}


def analysis_a(panel: pd.DataFrame, max_units) -> pd.DataFrame:
    rows = []
    for cause, cname in CAUSES.items():
        oof = {}
        for name, feats in FEATURE_SETS.items():
            log(f"A: GBM {cname} / {name} ({len(feats)} features)")
            oof[name], _ = fit_gbm_cause_specific(panel, feats, cause=cause)
            ci = design_ci_c_index(
                panel,
                oof[name],
```

```python
                cause=cause,
                design_frame_cols=DESIGN_COLS,
                n_cycles=10,
                max_units=max_units,
            )
            rows.append(
                {
                    "outcome": cname,
                    "model": name,
                    "n_features": len(feats),
                    **ci,
                }
            )
        common = oof["full"].dropna().index
        for other in FEATURE_SETS:
            common = common.intersection(oof[other].dropna().index)
        for name, feats in FEATURE_SETS.items():
            log(
                f"A: {cname} / {name} restricted to common complete-case sample"
            )
            ci = design_ci_c_index(
                panel.loc[common],
                oof[name].loc[common],
                cause=cause,
                design_frame_cols=DESIGN_COLS,
                n_cycles=10,
                max_units=max_units,
            )
            rows.append(
                {
                    "outcome": cname,
                    "model": f"{name}_common_sample",
                    "n_features": len(feats),
                    **ci,
                }
            )
        for base in ("age_only", "age_sex"):
            log(f"A: paired delta {cname} full - {base}")
            d = paired_delta_ci(
                panel, oof["full"], oof[base], cause, max_units
            )
            rows.append(
                {
                    "outcome": cname,
                    "model": f"full_minus_{base}",
                    "n_features": np.nan,
                    "c_index": np.nan,
                    "c_index_lo": np.nan,
                    "c_index_hi": np.nan,
                    **d,
                }
            )
    out = pd.DataFrame(rows)
    out.to_csv(
        RESULTS_METRICS / "baseline_c_index_comparison.csv", index=False
    )
    log(f"A: wrote {RESULTS_METRICS / 'baseline_c_index_comparison.csv'}")
    return out


def comorbidity_specs():
    base = list(MODEL_FEATURES)
    m2 = [f if f != "comorbidity_index" else "comorbidity_cvd5" for f in base]
    m3 = [f for f in base if f != "comorbidity_index"]
    return {
        "M1_published": (base, "comorbidity_index"),
        "M2_cvd5": (m2, "comorbidity_cvd5"),
        "M3_no_index": (m3, None),
    }


def analysis_b(
    panel: pd.DataFrame, max_units, design_ci: bool
) -> pd.DataFrame:
    rows = []
    for spec, (feats, term) in comorbidity_specs().items():
```

```
        cc = panel.dropna(
            subset=feats + ["followup_years", "event", "seqn"]
        ).copy()
        design_cc = survey.build(
            cc.rename(
                columns={
                    "psu": "SDMVPSU",
                    "strata": "SDMVSTRA",
                    "weight_mec": "WTMEC2YR",
                }
            ),
            "mec",
            n_cycles=10,
        )
        for cause, cname in CAUSES.items():
            log(
                f"B: Fine-Gray {spec} / {cname} (n={len(cc)}, {len(feats)} features)"
            )
            ctv, _ = fit_finegray_cox(
                cc,
                feats,
                "followup_years",
                "event",
                cause=cause,
                design=design_cc,
                penalizer=0.5,
            )
            hr = hr_table(ctv)
            want = [
                t
                for t in ([term] if term else [])
                + ["hypertension", "diabetes"]
                if t
            ]
            for t in want:
                r = hr[hr["feature"] == t]
                if r.empty:
                    continue
                r = r.iloc[0]
                rows.append(
                    {
                        "spec": spec,
                        "outcome": cname,
                        "term": t,
                        "n": len(cc),
                        "HR": r["HR"],
                        "HR_lo_model": r.get("HR_lo"),
                        "HR_hi_model": r.get("HR_hi"),
                        "p_model": r.get("p"),
                    }
                )
            if design_ci:
                want_ci = ([term] if term else []) + [
                    "hypertension",
                    "diabetes",
                ]
                log(
                    f"B: design jackknife CI {spec}/{cname} "
                    f"({len(want_ci)} coefficients, max_units={max_units})"
                )
                ci = design_ci_for_coefficients(
                    cc,
                    feats,
                    "followup_years",
                    "event",
                    cause=cause,
                    design=design_cc,
                    coefficients=want_ci,
                    max_units=max_units,
                    penalizer=0.5,
                )
                for r in rows:
                    if r["spec"] == spec and r["outcome"] == cname:
                        c = ci[ci["feature"] == r["term"]]
                        if not c.empty:
                            r["HR_lo_design"] = c.iloc[0].get("HR_lo")
```

```python
                            r["HR_hi_design"] = c.iloc[0].get("HR_hi")
    out = pd.DataFrame(rows)
    out.to_csv(
        RESULTS_TABLES / "comorbidity_index_decomposition.csv", index=False
    )
    log(f"B: wrote {RESULTS_TABLES / 'comorbidity_index_decomposition.csv'}")
    return out


def main() -> int:
    ap = argparse.ArgumentParser()
    ap.add_argument("--stage", default="all", choices=["A", "B", "all"])
    ap.add_argument("--max-units", type=int, default=None)
    ap.add_argument(
        "--design-ci",
        action="store_true",
        help="also run the expensive refitting jackknife in Analysis B",
    )
    a = ap.parse_args()
    if a.max_units == 0:
        a.max_units = None
    panel = load_panel()
    if a.stage in ("A", "all"):
        print(
            analysis_a(panel, a.max_units).to_string(index=False), flush=True
        )
    if a.stage in ("B", "all"):
        print(
            analysis_b(panel, a.max_units, a.design_ci).to_string(index=False),
            flush=True,
        )
    log("done")
    return 0


if __name__ == "__main__":
    sys.exit(main())
```

23 src/mi_sensitivity.py

```python
from __future__ import annotations

import numpy as np
import pandas as pd
from statsmodels.duration.hazard_regression import PHReg
from statsmodels.imputation.mice import MICEData

MI_FEATURES = [
    "age",
    "sex_female",
    "bmi",
    "sbp",
    "hba1c",
    "crp",
    "hemoglobin",
    "hypertension",
    "diabetes",
    "time_since_diagnosis_years",
    "n_cancer_sites",
]


def mi_trigger_note(
    panel: pd.DataFrame, features: list[str]
) -> tuple[float, str]:
    cc_n = panel[features].dropna().shape[0]
    pct_dropped = 100 * (1 - cc_n / len(panel))
    note = (
        f"Listwise deletion on the {len(features)}-feature model set drops "
        f"{pct_dropped:.1f}% of the survivor cohort (complete-case n={cc_n} of "
        f"{len(panel)}), exceeding the pre-specified 10% threshold for triggering "
        f"a multiple-imputation sensitivity analysis."
    )
```

```python
    return pct_dropped, note


def _fit_cox(
    data: pd.DataFrame, features: list[str]
) -> tuple[np.ndarray, np.ndarray]:
    model = PHReg(
        data["followup_years"].to_numpy(dtype=float),
        data[features].to_numpy(dtype=float),
        status=data["status"].to_numpy(dtype=float),
        ties="breslow",
    )
    res = model.fit()
    return np.asarray(res.params), np.asarray(res.bse)


def run_mi_sensitivity(
    panel: pd.DataFrame,
    cause: int,
    features: list[str] = None,
    n_imputations: int = 10,
    n_burnin: int = 10,
    seed: int = 42,
) -> pd.DataFrame:
    features = features or MI_FEATURES
    data = panel[features + ["followup_years", "event"]].copy()
    for c in data.columns:
        if str(data[c].dtype) == "boolean":
            data[c] = data[c].astype("float")
        data[c] = pd.to_numeric(data[c], errors="coerce")
    data = data.dropna(subset=["followup_years", "event"])
    data["status"] = (data["event"] == cause).astype(float)
    data = data.drop(columns=["event"])

    cc = data.dropna()
    cc_b, cc_se = _fit_cox(cc, features)
    cc_rows = pd.DataFrame(
        {"feature": features, "cc_coef": cc_b, "cc_se": cc_se, "cc_n": len(cc)}
    )

    np.random.seed(seed)
    imp = MICEData(data)
    imp.update_all(n_burnin)
    betas, variances = [], []
    for _ in range(n_imputations):
        imp.update_all(1)
        b, se = _fit_cox(imp.data, features)
        betas.append(b)
        variances.append(se**2)
    betas, variances = np.array(betas), np.array(variances)
    m = n_imputations
    qbar = betas.mean(axis=0)
    total_var = variances.mean(axis=0) + (1 + 1 / m) * betas.var(
        axis=0, ddof=1
    )
    mi_rows = pd.DataFrame(
        {
            "feature": features,
            "mi_coef": qbar,
            "mi_se": np.sqrt(total_var),
            "mi_n": len(data),
        }
    )

    out = cc_rows.merge(mi_rows, on="feature", how="outer")
    out["coef_shift"] = out["mi_coef"] - out["cc_coef"]
    out["same_sign"] = np.sign(out["mi_coef"]) == np.sign(out["cc_coef"])
    return out
```

24 src/crp_sensitivity.py

```
from __future__ import annotations

import time
import warnings
from pathlib import Path

warnings.filterwarnings("ignore")

ROOT = Path(__file__).resolve().parents[1]

import numpy as np
import pandas as pd
from scipy import stats
from statsmodels.stats.multitest import multipletests

from src import survey
from src.audits import MODEL_FEATURES
from src.cardiotoxicity import cardiotoxicity_group
from src.cohort import build_cohort
from src.config import N_JACKKNIFE_MAX_UNITS, RESULTS_TABLES
from src.covariates import build_features
from src.finegray import design_ci_for_coefficients
from src.outcomes import attach_outcomes

T0 = time.time()


def log(msg: str) -> None:
    print(f"[{time.time() - T0:7.1f}s] {msg}", flush=True)


def design_frame_of(df: pd.DataFrame) -> pd.DataFrame:
    return df.rename(
        columns={
            "psu": "SDMVPSU",
            "strata": "SDMVSTRA",
            "weight_mec": "WTMEC2YR",
        }
    )


def design_holm(ci: pd.DataFrame, coefficients: list[str]) -> pd.DataFrame:
    d = ci.set_index("feature").loc[coefficients]
    df_resid = d["df"].to_numpy()
    se = (d["coef_hi"].to_numpy() - d["coef_lo"].to_numpy()) / (
        2.0 * stats.t.ppf(0.975, df_resid)
    )
    t_stat = d["coef"].to_numpy() / se
    p_raw = 2.0 * stats.t.sf(np.abs(t_stat), df_resid)
    reject, p_adj, _, _ = multipletests(p_raw, alpha=0.05, method="holm")
    return pd.DataFrame(
        {
            "feature": coefficients,
            "coef": d["coef"].to_numpy(),
            "se_design": se,
            "df": df_resid,
            "t_stat": t_stat,
            "p_raw_design": p_raw,
            "p_adj_design": p_adj,
            "reject_h0": reject,
        }
    )


def main() -> None:
    cohort, _ = build_cohort()
    panel = build_features(attach_outcomes(cohort))
    panel["cardiotox_group"] = cardiotoxicity_group(panel)

    features = [f for f in MODEL_FEATURES if f != "crp"]
    keys = ["followup_years", "event", "seqn"]
    cc_with = panel.dropna(subset=MODEL_FEATURES + keys)
    cc = panel.dropna(subset=features + keys).copy()
    log(f"complete-case n without CRP = {len(cc)} (with CRP: {len(cc_with)})")
```

```python

    cov = (
        pd.DataFrame(
            {
                "cohort": panel["cycle"].value_counts(),
                "complete_case_with_crp": cc_with["cycle"].value_counts(),
                "complete_case_without_crp": cc["cycle"].value_counts(),
            }
        )
        .fillna(0)
        .astype(int)
        .sort_index()
    )
    cov.index.name = "cycle"
    cov.to_csv(RESULTS_TABLES / "crp_cycle_coverage.csv")
    print(cov.to_string())

    design_cc = survey.build(design_frame_of(cc), "mec", n_cycles=10)
    headline = [
        "age",
        "sex_female",
        "time_since_diagnosis_years",
        "hypertension",
        "diabetes",
        "bmi",
        "n_cancer_sites",
        "comorbidity_index",
        "smoking_status_never",
    ]
    for cause, name in ((1, "cvd"), (2, "cancer")):
        log(f"design-aware jackknife CI without CRP ({name})")
        ci = design_ci_for_coefficients(
            cc,
            features,
            "followup_years",
            "event",
            cause=cause,
            design=design_cc,
            coefficients=headline,
            max_units=N_JACKKNIFE_MAX_UNITS,
            penalizer=0.5,
        )
        ci.to_csv(
            RESULTS_TABLES / f"finegray_{name}_death_design_ci_no_crp.csv",
            index=False,
        )
        design_holm(ci, headline).to_csv(
            RESULTS_TABLES / f"holm_bonferroni_{name}_no_crp.csv", index=False
        )
        row = ci.set_index("feature").loc["comorbidity_index"]
        log(
            f"{name}: comorbidity index HR {row.HR:.3f} ({row.HR_lo:.3f}-{row.HR_hi:.3f})"
        )
    log("done")


if __name__ == "__main__":
    main()
```

25 src/design_based_intervals.py

```python
from __future__ import annotations

import time
import warnings
from pathlib import Path

warnings.filterwarnings("ignore")

ROOT = Path(__file__).resolve().parents[1]

import pandas as pd

```

```python
from src import survey
from src.audits import MODEL_FEATURES
from src.cardiotoxicity import cardiotoxicity_group
from src.cohort import build_cohort
from src.config import RESULTS_TABLES
from src.covariates import build_features
from src.finegray import design_ci_for_coefficients
from src.outcomes import attach_outcomes

T0 = time.time()


def log(msg: str) -> None:
    print(f"[{time.time() - T0:7.1f}s] {msg}", flush=True)


def design_frame_of(df: pd.DataFrame) -> pd.DataFrame:
    return df.rename(
        columns={
            "psu": "SDMVPSU",
            "strata": "SDMVSTRA",
            "weight_mec": "WTMEC2YR",
        }
    )


PAPER_DIR = ROOT
NAIVE_FILES = [
    "finegray_cvd_death_full_model.csv",
    "finegray_cancer_death_full_model.csv",
    "cardiotoxicity_cvd_death_model.csv",
    "cardiotoxicity_time_interaction_model.csv",
]


def main() -> None:
    log("rebuilding cohort/panel (cached)")
    cohort, _inv = build_cohort()
    df = attach_outcomes(cohort)
    panel = build_features(df)
    panel["cardiotox_group"] = cardiotoxicity_group(panel)

    coef_lists = {}
    for fname in NAIVE_FILES:
        coef_lists[fname] = pd.read_csv(RESULTS_TABLES / fname)[
            "feature"
        ].tolist()

    cc = panel.dropna(
        subset=MODEL_FEATURES + ["followup_years", "event", "seqn"]
    ).copy()
    design_cc = survey.build(design_frame_of(cc), "mec", n_cycles=10)
    log(
        f"Fine-Gray complete-case n={len(cc)}; computing design CI for ALL "
        f"{len(coef_lists['finegray_cvd_death_full_model.csv'])} coefficients (CVD)"
    )
    ci_cvd_full = design_ci_for_coefficients(
        cc,
        MODEL_FEATURES,
        "followup_years",
        "event",
        cause=1,
        design=design_cc,
        coefficients=coef_lists["finegray_cvd_death_full_model.csv"],
        max_units=None,
        penalizer=0.5,
    )
    ci_cvd_full.to_csv(
        RESULTS_TABLES / "finegray_cvd_death_full_model_design_ci.csv",
        index=False,
    )
    log("CVD full-model design CI done")

    log("computing design CI for ALL coefficients (cancer)")
    ci_cancer_full = design_ci_for_coefficients(
        cc,
```

```
        MODEL_FEATURES,
        "followup_years",
        "event",
        cause=2,
        design=design_cc,
        coefficients=coef_lists["finegray_cancer_death_full_model.csv"],
        max_units=None,
        penalizer=0.5,
    )
    ci_cancer_full.to_csv(
        RESULTS_TABLES / "finegray_cancer_death_full_model_design_ci.csv",
        index=False,
    )
    log("cancer full-model design CI done")

    ct_features = [f for f in MODEL_FEATURES if f != "n_cancer_sites"] + [
        "cardiotox_high"
    ]
    cc_ct = panel[
        panel["cardiotox_group"].isin(
            ["High expected cardiotoxicity", "Low expected cardiotoxicity"]
        )
    ].copy()
    cc_ct["cardiotox_high"] = (
        cc_ct["cardiotox_group"] == "High expected cardiotoxicity"
    )
    cc_ct = cc_ct.dropna(
        subset=ct_features + ["followup_years", "event", "seqn"]
    )
    design_ct = survey.build(design_frame_of(cc_ct), "mec", n_cycles=10)
    log(
        f"cardiotoxicity complete-case n={len(cc_ct)}; computing design CI for ALL "
        f"{len(coef_lists['cardiotoxicity_cvd_death_model.csv'])} coefficients"
    )
    ci_ct_full = design_ci_for_coefficients(
        cc_ct,
        ct_features,
        "followup_years",
        "event",
        cause=1,
        design=design_ct,
        coefficients=coef_lists["cardiotoxicity_cvd_death_model.csv"],
        max_units=None,
        penalizer=0.5,
    )
    ci_ct_full.to_csv(
        RESULTS_TABLES / "cardiotoxicity_cvd_death_model_design_ci.csv",
        index=False,
    )
    log("cardiotoxicity full-model design CI done")

    cc_ct2 = cc_ct.copy()
    cc_ct2["cardiotox_x_time"] = (
        cc_ct2["cardiotox_high"].astype(float)
        * cc_ct2["time_since_diagnosis_years"]
    )
    ct2_features = ct_features + ["cardiotox_x_time"]
    log(
        f"interaction model complete-case n={len(cc_ct2)}; computing design CI for ALL "
        f"{len(coef_lists['cardiotoxicity_time_interaction_model.csv'])} coefficients"
    )
    ci_ct2_full = design_ci_for_coefficients(
        cc_ct2,
        ct2_features,
        "followup_years",
        "event",
        cause=1,
        design=design_ct,
        coefficients=coef_lists["cardiotoxicity_time_interaction_model.csv"],
        max_units=None,
        penalizer=0.5,
    )
    ci_ct2_full.to_csv(
        RESULTS_TABLES / "cardiotoxicity_time_interaction_model_design_ci.csv",
        index=False,
    )
```

```
    log("interaction full-model design CI done")


if __name__ == "__main__":
    main()
```

26 src/condition_answer_codes.py

```
from __future__ import annotations

import warnings
from pathlib import Path

warnings.filterwarnings("ignore")

ROOT = Path(__file__).resolve().parents[1]

import pandas as pd

from src.audits import MODEL_FEATURES
from src.cohort import build_cohort
from src.config import RESULTS_TABLES
from src.covariates import build_features
from src.outcomes import attach_outcomes

ITEMS = [
    "BPQ020",
    "DIQ010",
    "MCQ160B",
    "MCQ160C",
    "MCQ160D",
    "MCQ160E",
    "MCQ160F",
]
LABEL = {1: "yes", 2: "no", 3: "borderline", 7: "refused", 9: "dont_know"}


def counts(frame: pd.DataFrame, sample: str) -> list[dict]:
    rows = []
    for item in ITEMS:
        v = (
            pd.to_numeric(frame[item], errors="coerce")
            if item in frame
            else pd.Series(dtype=float)
        )
        coded = v.map(LABEL).fillna("blank")
        vc = coded.value_counts()
        row = {"sample": sample, "n": len(frame), "item": item}
        for k in ["yes", "no", "borderline", "refused", "dont_know", "blank"]:
            row[k] = int(vc.get(k, 0))
        row["counted_not_reported_non_no"] = (
            row["borderline"]
            + row["refused"]
            + row["dont_know"]
            + row["blank"]
        )
        rows.append(row)
    return rows


def main() -> None:
    cohort, _ = build_cohort()
    df = attach_outcomes(cohort)
    panel = build_features(df)
    cc_mask = (
        panel[MODEL_FEATURES + ["followup_years", "event"]]
        .notna()
        .all(axis=1)
        .to_numpy()
    )
    rows = counts(df, "analytic_cohort") + counts(
        df.loc[cc_mask], "complete_case"
    )
```

```python
    out = pd.DataFrame(rows)
    out.to_csv(RESULTS_TABLES / "condition_item_answer_codes.csv", index=False)
    print(out.to_string(index=False))


if __name__ == "__main__":
    main()
```

27 src/fmt.py

```python
from __future__ import annotations

import math
import re

MINUS = "−"
EN_DASH = "–"
TIMES = "×"
ARROW = " → "

DECIMALS: dict[str, int] = {
    "auroc": 3,
    "auprc": 3,
    "c_index": 3,
    "r2": 3,
    "auc_diff": 4,
    "percent": 1,
    "sensitivity": 1,
    "specificity": 1,
    "accuracy": 1,
    "coef": 3,
    "beta": 3,
    "log_hr": 3,
    "shap": 3,
    "hr": 2,
    "or": 2,
    "rr": 2,
    "pvalue": 3,
    "brier": 3,
    "calibration_slope": 3,
    "clinical": 1,
}


def u(text: str) -> str:
    return re.sub(r"(?<![\w-])-(?=\d)", MINUS, text)


def fmt_n(n: float | int) -> str:
    n = int(round(n))
    return f"{n:,}" if abs(n) >= 10000 else str(n)


def num(value: float, kind: str = "coef", *, signed: bool = False) -> str:
    if value is None or (isinstance(value, float) and math.isnan(value)):
        return "NA"
    dp = DECIMALS.get(kind, 3)
    s = f"{value:+.{dp}f}" if signed else f"{value:.{dp}f}"
    return s.replace("-", MINUS)


def pct(
    value: float, *, already_fraction: bool = False, signed: bool = False
) -> str:
    v = value * 100 if already_fraction else value
    return num(v, "percent", signed=signed) + "%"


def pval(p: float) -> str:
    if p is None or (isinstance(p, float) and math.isnan(p)):
        return "NA"
    if p < 0.001:
        return "p < 0.001"
```

```
    return f"p = {p:.3f}"


def ci(
    lo: float, hi: float, kind: str = "auroc", *, prefix: str = "95% CI: "
) -> str:
    return f"{prefix}{num(lo, kind)}{EN_DASH}{num(hi, kind)}"


def est_ci(point: float, lo: float, hi: float, kind: str = "auroc") -> str:
    return f"{num(point, kind)} ({ci(lo, hi, kind)})"


def sci(value: float, sig: int = 1) -> str:
    if value == 0:
        return "0"
    exp = int(math.floor(math.log10(abs(value))))
    mant = value / (10**exp)
    mant_s = f"{mant:.{sig}f}".rstrip("0").rstrip(".").replace("-", MINUS)
    sup = str(exp).replace("-", "⁻")
    trans = str.maketrans("0123456789", "⁰¹²³⁴⁵⁶⁷⁸⁹")
    return f"{mant_s} {TIMES} 10{sup.translate(trans)}"


def unit(value: float, unit_str: str, kind: str = "clinical") -> str:
    return f"{num(value, kind)} {unit_str}"


def italic_n(n: float | int) -> str:
    return f"$\\it{{n}}$ = {fmt_n(n)}"


def check_text(text: str) -> list[str]:
    problems: list[str] = []
    masked = text
    for pat in (
        r"\bdoi:\s*\S+",
        r"https?://\S+",
        r"\bG(?:SE|PL|SM)\d+\b",
        r"\b10\.\d{4,9}/\S+",
        r"\bBG-RRP-\d\.\d{3}-\d{4}\b",
    ):
        masked = re.sub(pat, lambda m: " " * len(m.group(0)), masked)

    for m in re.finditer(r"(?<![\d,])\b(\d{4})\b(?!\d)", masked):
        if "," in m.group(0):
            problems.append(f"4-digit number with comma: {m.group(0)!r}")
    for m in re.finditer(r"(?<![\d,])\b\d{1,3},\d{3}\b(?![\d,])", masked):
        digits = m.group(0).replace(",", "")
        if len(digits) == 4:
            problems.append(f"comma on a 4-digit number: {m.group(0)!r}")
    for m in re.finditer(r"(?<![\w-])-\d", masked):
        problems.append(
            f"ASCII hyphen used as minus: {masked[max(0, m.start()-20):m.start()+10]!r}"
        )
    for m in re.finditer(r"\d[Ee][+-]?\d", masked):
        problems.append(f"E-notation, use {TIMES} 10^n: {m.group(0)!r}")
    for _m in re.finditer(r"p\s*=\s*0\.000\b", masked):
        problems.append("p = 0.000, use p < 0.001")
    for m in re.finditer(r"\d-\d", masked):
        window = masked[max(0, m.start() - 90) : m.end() + 24]
        if (
            re.search(r"\bORCID\b", window, re.I)
            or re.search(r"\b\d{4}-\d{2}-\d{2}\b", window)
            or re.search(r"\b\d{2}-\d{3}\s+[A-Z]", window)
        ):
            continue
        problems.append(
            f"hyphen in a numeric range, use en dash: {m.group(0)!r}"
        )
    for m in re.finditer(r"\b\d+(\.\d+)?(M|kg|mmHg|mg/dL|mmol/L|%)\b", masked):
        problems.append(f"missing space before unit: {m.group(0)!r}")
    return problems
```

28 src/viz.py

```python
from __future__ import annotations

import json
import re
from pathlib import Path

import matplotlib as mpl
import numpy as np

PALETTE: dict[str, str] = {
    "blue": "#0072B2",
    "orange": "#E69F00",
    "green": "#009E73",
    "pink": "#CC79A7",
    "red": "#D55E00",
    "sky": "#56B4E9",
    "yellow": "#F0E442",
    "grey": "#999999",
}
ORDER = ["blue", "orange", "green", "pink", "red", "sky", "yellow", "grey"]
LINESTYLES = [
    "-",
    "--",
    "-.",
    ":",
    (0, (3, 1, 1, 1)),
    (0, (5, 1)),
    (0, (1, 1)),
    (0, (3, 5, 1, 5)),
]

WIDTH_FULL = 5.5
WIDTH_NARROW = 4.5


def apply_style() -> None:
    mpl.rcParams.update(
        {
            "axes.unicode_minus": True,
            "font.family": "serif",
            "font.size": 10,
            "figure.dpi": 300,
            "savefig.dpi": 300,
            "mathtext.default": "it",
            "axes.grid": False,
            "axes.spines.top": False,
            "axes.spines.right": False,
            "legend.frameon": False,
            "axes.titlesize": 10,
        }
    )


def series(i: int) -> dict:
    return {
        "color": PALETTE[ORDER[i % len(ORDER)]],
        "linestyle": LINESTYLES[i % len(LINESTYLES)],
    }


def panel(ax, letter: str) -> None:
    ax.text(
        0.02,
        0.98,
        f"({letter})",
        transform=ax.transAxes,
        fontweight="bold",
        va="top",
        ha="left",
    )


def _collect(fig) -> dict:
    handles: list[dict] = []
```

```
    for ax in fig.get_axes():
        for line in ax.get_lines():
            lbl = line.get_label()
            if lbl and not lbl.startswith("_"):
                handles.append(
                    {
                        "type": "line",
                        "label": lbl,
                        "color": mpl.colors.to_hex(line.get_color()),
                        "linestyle": str(line.get_linestyle()),
                    }
                )
        for patch in ax.patches:
            lbl = patch.get_label()
            if lbl and not lbl.startswith("_"):
                handles.append(
                    {
                        "type": "patch",
                        "label": lbl,
                        "color": mpl.colors.to_hex(patch.get_facecolor()),
                    }
                )
        for coll in ax.collections:
            lbl = coll.get_label()
            if lbl and not lbl.startswith("_"):
                handles.append({"type": "collection", "label": lbl})
        seen = {h["label"] for h in handles}
        for container in getattr(ax, "containers", []):
            lbl = container.get_label()
            if not lbl or lbl.startswith("_") or lbl in seen:
                continue
            color = None
            for child in container.get_children():
                if hasattr(child, "get_facecolor"):
                    fc = np.asarray(child.get_facecolor())
                    if fc.ndim == 2:
                        if len(fc) == 0:
                            continue
                        fc = fc[0]
                    color = mpl.colors.to_hex(fc)
                    break
            entry = {"type": "container", "label": lbl}
            if color:
                entry["color"] = color
            handles.append(entry)
            seen.add(lbl)
    return {"handles": handles, "n_axes": len(fig.get_axes())}


def save(
    fig, outdir: Path | str, name: str, *, tight: bool = True
) -> dict[str, Path]:
    outdir = Path(outdir)
    outdir.mkdir(parents=True, exist_ok=True)
    kw = {"bbox_inches": "tight"} if tight else {}
    png = outdir / f"{name}.png"
    pdf = outdir / f"{name}.pdf"
    fig.savefig(png, dpi=300, **kw)
    fig.savefig(pdf, **kw)
    sidecar = outdir / f"{name}.colors.json"
    sidecar.write_text(json.dumps(_collect(fig), indent=2), encoding="utf-8")
    return {"png": png, "pdf": pdf, "sidecar": sidecar}


def forest_figsize(n_rows: int) -> tuple[float, float]:
    return (9.0, max(4.5, n_rows * 0.42))


def adjust_forest(fig) -> None:
    fig.subplots_adjust(left=0.28, right=0.97, top=0.94, bottom=0.10)


_LEADING_BOLD_LABEL = re.compile(r"^\s*\*\*(Figure|Table)\s+\d+\.\*\*\s*")


def check_caption(caption: str, sidecar: Path | str) -> list[str]:
```

```
    problems: list[str] = []
    data = json.loads(Path(sidecar).read_text(encoding="utf-8"))
    marker_scan_text = _LEADING_BOLD_LABEL.sub("", caption)
    marker_scan_text = re.sub(r"\*(\S(?:[^*]*\S)?)\*", "", marker_scan_text)
    caption_plain = re.sub(r"\*{1,3}(\S(?:[^*]*\S)?)\*{1,3}", r"\1", caption)
    for h in data["handles"]:
        if h["label"] not in caption and h["label"] not in caption_plain:
            problems.append(
                f"legend element {h['label']!r} not described in caption"
            )
    for marker in ("***", "**", "*"):
        if marker in marker_scan_text and not any(
            marker in h.get("label", "") for h in data["handles"]
        ):
            problems.append(
                f"caption claims significance marker {marker!r}; verify it is drawn"
            )
            break
    return problems
```

29 src/figures.py

```
from __future__ import annotations

import json
from pathlib import Path

import numpy as np
import pandas as pd

ROOT = Path(__file__).resolve().parents[1]

import matplotlib.pyplot as plt

from src import config as cfg, fmt, viz

FIGDIR = cfg.RESULTS_FIGURES

LABELS: dict[str, str] = {
    "age": "Age",
    "sex_female": "Female sex",
    "time_since_diagnosis_years": "Time since diagnosis",
    "hypertension": "Hypertension",
    "diabetes": "Diabetes",
    "crp": "C-reactive protein",
    "bmi": "Body-mass index",
    "n_cancer_sites": "Number of cancer sites",
    "comorbidity_index": "Comorbidity index",
    "smoking_status_never": "Never smoker",
    "smoking_status": "Smoking status",
    "sbp": "Systolic blood pressure",
    "dbp": "Diastolic blood pressure",
    "nlr": "Neutrophil-to-lymphocyte ratio",
    "pir": "Income-to-poverty ratio",
    "hba1c": "HbA1c",
    "hdl": "HDL cholesterol",
    "total_chol": "Total cholesterol",
    "serum_albumin": "Serum albumin",
    "hemoglobin": "Haemoglobin",
    "healthcare_visits_12mo": "Healthcare visits, 12 months",
    "general_health_rating": "Self-rated general health",
    "race_eth": "Race/ethnicity",
    "education": "Education",
    "alcohol_ever_heavy": "Alcohol, ≥12 drinks in any year",
    "physically_active": "Physically active",
}


def label(feature: str) -> str:
    return LABELS.get(feature, feature.replace("_", " ").capitalize())


def fig_study_flow() -> dict:
```

```
    inv = pd.read_csv(cfg.RESULTS_TABLES / "subsample_inventory.csv")
    lookup = dict(zip(inv["label"], inv["n"], strict=False))

    keep = [
        (
            "NHANES 1999-2018 participants",
            "NHANES 1999-2018, DEMO merged with MCQ on SEQN",
        ),
        ("Adults ≥ 20 y", "Adults >=20 y (all cycles)"),
        (
            "Valid MEC examination weight",
            "Adults >=20 y with a valid MEC exam weight",
        ),
        (
            "Physician-diagnosed cancer (MCQ220 = 1)",
            "... reporting a physician diagnosis of cancer (MCQ220 = 1)",
        ),
    ]
    steps = [(disp, int(lookup[key])) for disp, key in keep if key in lookup]

    n_final = int(inv[inv["label"].str.startswith("FINAL")]["n"].iloc[0])
    excl_row = inv[inv["label"].str.startswith("Excluding survivors")]
    n_excl = int(excl_row["n"].iloc[0]) if len(excl_row) else None
    n_site = int(
        lookup.get(
            "... with an identifiable cancer site (MCQ230/MCQ240 items)", 0
        )
    )
    n_tsd = int(
        lookup.get("Survivor cohort with a computable time since diagnosis", 0)
    )

    n_diagnosed = steps[-1][1]
    if n_excl is not None and n_diagnosed - n_excl != n_final:
        raise ValueError(
            f"study-flow arithmetic does not close: {n_diagnosed} - {n_excl} "
            f"= {n_diagnosed - n_excl}, but the final cohort is {n_final}. "
            "Fix the inventory or the cascade before publishing this figure."
        )
    steps.append(("Final analytic survivor cohort", n_final))

    viz.apply_style()
    fig, ax = plt.subplots(figsize=(viz.WIDTH_FULL, 5.8))
    ax.set_axis_off()

    box_h, gap = 0.9, 0.62
    y = float(len(steps)) * (box_h + gap)
    box_w, x0 = 6.6, 0.4
    for i, (text, n) in enumerate(steps):
        colour = (
            viz.PALETTE["blue"] if i < len(steps) - 1 else viz.PALETTE["green"]
        )
        ax.add_patch(
            plt.Rectangle(
                (x0, y),
                box_w,
                box_h,
                facecolor="white",
                edgecolor=colour,
                linewidth=1.4,
                zorder=2,
            )
        )
        ax.text(
            x0 + box_w / 2,
            y + box_h * 0.60,
            text,
            ha="center",
            va="center",
            fontsize=8.5,
            zorder=3,
        )
        ax.text(
            x0 + box_w / 2,
            y + box_h * 0.22,
            f"$n$ = {fmt.fmt_n(n)}",
```

```
            ha="center",
            va="center",
            fontsize=8.5,
            fontweight="bold",
            zorder=3,
        )
        if i < len(steps) - 1:
            ax.annotate(
                "",
                xy=(x0 + box_w / 2, y - gap * 0.05),
                xytext=(x0 + box_w / 2, y - gap * 0.95),
                arrowprops={
                    "arrowstyle": "<|-",
                    "color": viz.PALETTE["grey"],
                    "linewidth": 1.0,
                },
            )
        y -= box_h + gap

    if n_excl is not None:
        ax.text(
            x0 + box_w + 0.15,
            y + box_h + gap * 0.5,
            f"— {fmt.fmt_n(n_excl)} excluded:\nskin-cancer-only or\nunknown histology",
            ha="left",
            va="center",
            fontsize=7.5,
            color=viz.PALETTE["grey"],
        )

    notes = []
    if n_site:
        notes.append(
            f"cancer site identifiable in {fmt.fmt_n(n_site)} of the "
            f"{fmt.fmt_n(n_diagnosed)} diagnosis reports"
        )
    if n_tsd:
        notes.append(
            f"time since diagnosis computable for {fmt.fmt_n(n_tsd)} "
            f"of the {fmt.fmt_n(n_final)} survivors"
        )
    if notes:
        ax.text(
            x0,
            y + box_h * 0.30,
            "Field completeness (not exclusions):\n  " + "\n  ".join(notes),
            ha="left",
            va="top",
            fontsize=7.5,
            color=viz.PALETTE["grey"],
        )

    top = float(len(steps)) * (box_h + gap) + box_h
    ax.set_xlim(0, 10.4)
    ax.set_ylim(y - box_h * 1.1, top + 0.75)
    return viz.save(fig, FIGDIR, "fig1_study_flow")


def fig_cumulative_incidence() -> dict:
    from lifelines import AalenJohansenFitter

    df = pd.read_parquet(
        cfg.DATA_PROCESSED / "survivor_cohort_outcomes.parquet"
    )
    df = df[df["MORTSTAT"].notna()].copy()

    causes = [
        (1, "Cardiovascular death"),
        (2, "Cancer death"),
        (3, "Other-cause death"),
    ]

    viz.apply_style()
    fig, ax = plt.subplots(figsize=(viz.WIDTH_FULL, 4.2))
    counts, cif_at = {}, {}
    for i, (code, name) in enumerate(causes):
```

```
        ajf = AalenJohansenFitter(
            calculate_variance=False, seed=cfg.RANDOM_SEED
        )
        ajf.fit(
            df["followup_years"],
            df["event"],
            event_of_interest=code,
            weights=df["WTMEC2YR"],
        )
        cif = ajf.cumulative_density_
        ax.plot(
            cif.index,
            100.0 * cif.iloc[:, 0].to_numpy(),
            label=name,
            linewidth=1.4,
            **viz.series(i),
        )
        counts[name] = int((df["event"] == code).sum())
        cif_at[name] = {
            t: round(float(cif[cif.index <= t].iloc[-1, 0]) * 100.0, 2)
            for t in (10.0, 15.0, 20.0)
        }

    ax.set_xlabel("Years since NHANES examination")
    ax.set_ylabel("Cumulative incidence (%)")
    ax.set_xlim(0, 20)
    ax.set_ylim(0, None)
    ax.legend(fontsize=8, loc="upper left", bbox_to_anchor=(0.02, 0.92))
    out = viz.save(fig, FIGDIR, "fig2_cumulative_incidence")
    out["counts"] = counts
    out["cif_at"] = cif_at
    return out


def fig_finegray_forest() -> dict:
    cvd = pd.read_csv(
        cfg.RESULTS_TABLES / "finegray_cvd_death_design_ci_headline.csv"
    )
    can = pd.read_csv(
        cfg.RESULTS_TABLES / "finegray_cancer_death_design_ci_headline.csv"
    )

    order = list(cvd["feature"])
    can = can.set_index("feature").reindex(order).reset_index()

    viz.apply_style()
    n = len(order)
    fig, axes = plt.subplots(
        1, 2, figsize=(9.0, max(4.0, n * 0.40)), sharey=True
    )
    ypos = np.arange(n)[::-1]

    for ax, tab, name, letter, ci in (
        (axes[0], cvd, "Cardiovascular death", "A", 0),
        (axes[1], can, "Cancer death", "B", 2),
    ):
        colour = viz.PALETTE[viz.ORDER[ci]]
        ax.errorbar(
            tab["HR"],
            ypos,
            xerr=[tab["HR"] - tab["HR_lo"], tab["HR_hi"] - tab["HR"]],
            fmt="o",
            markersize=4,
            color=colour,
            ecolor=colour,
            elinewidth=1.1,
            capsize=2.5,
            linestyle="none",
        )
        ax.axvline(
            1.0, color=viz.PALETTE["grey"], linestyle=":", linewidth=1.0
        )
        ax.set_xscale("log")
        ax.set_xlabel(f"Subdistribution hazard ratio\n{name}")
        ax.set_xlim(0.1, 4.0)
        ax.set_xticks([0.125, 0.25, 0.5, 1, 2, 4])
```

```
        ax.set_xticklabels(["0.125", "0.25", "0.5", "1", "2", "4"])
        viz.panel(ax, letter)

    axes[0].set_yticks(ypos)
    axes[0].set_yticklabels([label(f) for f in order], fontsize=8.5)
    axes[0].set_ylim(-0.8, n - 0.2)
    fig.subplots_adjust(
        left=0.22, right=0.98, top=0.95, bottom=0.20, wspace=0.10
    )
    return viz.save(fig, FIGDIR, "fig3_finegray_forest", tight=False)


def fig_shap_importance(top_n: int = 12) -> dict:
    cvd = pd.read_csv(cfg.RESULTS_METRICS / "shap_global_cvd_death.csv")
    can = pd.read_csv(cfg.RESULTS_METRICS / "shap_global_cancer_death.csv")

    top = cvd.nlargest(top_n, "mean_abs_shap")["feature"].tolist()
    cvd_v = cvd.set_index("feature")["mean_abs_shap"].reindex(top).fillna(0.0)
    can_v = can.set_index("feature")["mean_abs_shap"].reindex(top).fillna(0.0)

    viz.apply_style()
    fig, ax = plt.subplots(figsize=(viz.WIDTH_FULL, max(3.6, top_n * 0.34)))
    ypos = np.arange(len(top))[::-1]
    h = 0.38
    ax.barh(
        ypos + h / 2,
        cvd_v.to_numpy(),
        height=h,
        color=viz.PALETTE["blue"],
        label="Cardiovascular death",
        edgecolor="none",
    )
    ax.barh(
        ypos - h / 2,
        can_v.to_numpy(),
        height=h,
        color=viz.PALETTE["green"],
        label="Cancer death",
        edgecolor="none",
    )
    ax.set_yticks(ypos)
    ax.set_yticklabels([label(f) for f in top], fontsize=8.5)
    ax.set_xlabel("Mean |SHAP| (log-hazard units)")
    ax.set_ylim(-0.8, len(top) - 0.2)
    ax.legend(fontsize=8, loc="lower right")
    return viz.save(fig, FIGDIR, "fig5_shap_importance")


def fig_calibration_dca() -> dict:
    cal = pd.read_csv(cfg.RESULTS_TABLES / "calibration_table_cvd_10y.csv")
    dca = pd.read_csv(cfg.RESULTS_TABLES / "decision_curve_cvd_10y.csv")
    brier = json.loads(
        (cfg.RESULTS_METRICS / "brier_cvd_10y.json").read_text()
    )

    viz.apply_style()
    fig, axes = plt.subplots(1, 2, figsize=(9.0, 3.9))

    ax = axes[0]
    hi = (
        float(
            max(cal["mean_predicted"].max(), cal["observed_proportion"].max())
        )
        + 0.05
    )
    ax.plot(
        [0, hi],
        [0, hi],
        color=viz.PALETTE["grey"],
        linestyle=":",
        linewidth=1.0,
        label="Perfect calibration",
    )
    ax.plot(
        cal["mean_predicted"],
        cal["observed_proportion"],
```

```
        marker="o",
        markersize=4,
        linewidth=1.3,
        label="Observed by risk decile",
        **viz.series(0),
    )
    ax.set_xlabel("Mean predicted 10-year risk")
    ax.set_ylabel("Observed proportion")
    ax.set_xlim(0, hi)
    ax.set_ylim(0, hi)
    ax.legend(fontsize=8, loc="upper left")
    ax.text(
        0.97,
        0.05,
        f"Brier = {fmt.num(brier['brier'], 'brier')}",
        transform=ax.transAxes,
        ha="right",
        va="bottom",
        fontsize=8,
    )
    viz.panel(ax, "A")

    ax = axes[1]
    ax.plot(
        dca["threshold"],
        dca["net_benefit_model"],
        linewidth=1.4,
        label="Competing-risk model",
        **viz.series(0),
    )
    ax.plot(
        dca["threshold"],
        dca["net_benefit_treat_all"],
        linewidth=1.1,
        label="Treat all",
        **viz.series(3),
    )
    ax.plot(
        dca["threshold"],
        dca["net_benefit_treat_none"],
        color="black",
        linewidth=0.9,
        label="Treat none",
    )
    ax.set_xlabel("Risk threshold probability")
    ax.set_ylabel("Net benefit")
    ax.set_xlim(float(dca["threshold"].min()), float(dca["threshold"].max()))
    ax.set_ylim(-0.02, float(dca["net_benefit_model"].max()) * 1.15)
    ax.legend(fontsize=8, loc="upper right")
    viz.panel(ax, "B")

    fig.subplots_adjust(
        left=0.09, right=0.98, top=0.95, bottom=0.16, wspace=0.30
    )
    return viz.save(
        fig, FIGDIR, "fig4_calibration_decision_curve", tight=False
    )


BUILDERS = [
    ("fig1_study_flow", fig_study_flow),
    ("fig2_cumulative_incidence", fig_cumulative_incidence),
    ("fig3_finegray_forest", fig_finegray_forest),
    ("fig4_calibration_decision_curve", fig_calibration_dca),
    ("fig5_shap_importance", fig_shap_importance),
]
```

30 src/make_figures.py

```python
from __future__ import annotations

import os

os.environ.setdefault("OMP_NUM_THREADS", "2")
os.environ.setdefault("OPENBLAS_NUM_THREADS", "2")
os.environ.setdefault("MKL_NUM_THREADS", "2")

import time
import traceback
from pathlib import Path

ROOT = Path(__file__).resolve().parents[1]

import matplotlib

matplotlib.use("Agg")

import pandas as pd

from src import config as cfg, figures, viz

CAPTIONS: dict[str, str] = {
    "fig1_study_flow": "**Figure 1.** Derivation of the analytic cancer-survivor cohort from pooled "
    "NHANES 1999-2018. Each box gives the sample remaining after the stated "
    "criterion. Survivors whose only reported malignancy was non-melanoma or "
    "unspecified skin cancer were excluded, following the registry convention "
    "that treats those as a distinct and much less lethal entity.",
    "fig2_cumulative_incidence": "**Figure 2.** Survey-weighted Aalen-Johansen cumulative incidence of "
    "Cardiovascular death, Cancer death and Other-cause death among cancer "
    "survivors, treating the three causes as competing events. Cumulative "
    "incidence rather than 1 — Kaplan-Meier: with three mutually exclusive "
    "causes the cause-specific complement of the survival function would "
    "overstate every curve.",
    "fig3_finegray_forest": "**Figure 3.** Fine-Gray subdistribution hazard ratios with 95% "
    "design-based confidence intervals from the stratified delete-one-PSU "
    "jackknife, for (A) cardiovascular death and (B) cancer death. The axis is "
    "logarithmic, so that reciprocal hazard ratios are equidistant from the "
    "null. The dotted vertical reference marks a hazard ratio of 1.",
    "fig5_shap_importance": "**Figure 5.** Mean absolute SHAP value per predictor in the "
    "gradient-boosted competing-risk models for Cardiovascular death and "
    "Cancer death, for the twelve predictors ranked highest by the "
    "cardiovascular model. Both series share that ordering so the two "
    "outcomes are read against each other rather than each against its own "
    "ranking.",
    "fig4_calibration_decision_curve": "**Figure 4.** (A) Calibration of the 10-year cardiovascular-death model by "
    "risk decile, showing Observed by risk decile against mean predicted risk, "
    "with the Perfect calibration diagonal for reference and the Brier score "
    "inset. (B) Decision-curve analysis: net benefit of the Competing-risk "
    "model against the Treat all and Treat none default strategies across "
    "risk thresholds.",
}


def main() -> None:
    cfg.RESULTS_FIGURES.mkdir(parents=True, exist_ok=True)
    built, failed, caption_problems = [], [], {}

    for name, builder in figures.BUILDERS:
        t0 = time.time()
        try:
            out = builder()
        except Exception:
            failed.append(name)
            print(f"[FAIL] {name}\n{traceback.format_exc()}", flush=True)
            continue
        built.append(name)
        extra = ""
        if "counts" in out:
            extra = "  events: " + ", ".join(
                f"{k}={v}" for k, v in out["counts"].items()
            )
        print(
```

```
            f"[ok]   {name} in {time.time()-t0:.1f}s -> {out['png'].name}, "
            f"{out['pdf'].name}{extra}",
            flush=True,
        )
        if "cif_at" in out:
            cif_rows = []
            for cause, at in out["cif_at"].items():
                print(
                    "       CIF "
                    + cause
                    + ": "
                    + ", ".join(f"{t:.0f}y={v:.2f}%" for t, v in at.items()),
                    flush=True,
                )
                cif_rows += [
                    {
                        "cause": cause,
                        "years": float(t),
                        "cif_pct": round(float(v), 1),
                    }
                    for t, v in at.items()
                ]
            pd.DataFrame(cif_rows).to_csv(
                cfg.RESULTS_TABLES / "cumulative_incidence_horizons.csv",
                index=False,
            )

        problems = viz.check_caption(CAPTIONS[name], out["sidecar"])
        if problems:
            caption_problems[name] = problems

    print("\n" + "=" * 70)
    print(f"figures built: {len(built)} / {len(figures.BUILDERS)}")
    if failed:
        print(f"FAILED: {', '.join(failed)}")
    if caption_problems:
        print(
            "\nCAPTION AUDIT PROBLEMS (fix the caption or the figure, not the audit):"
        )
        for name, probs in caption_problems.items():
            for p in probs:
                print(f"  {name}: {p}")
    else:
        print("caption audit: all built figures clean")

    captions_out = cfg.RESULTS_FIGURES / "captions.md"
    captions_out.write_text(
        "\n\n".join(CAPTIONS[n] for n in built), encoding="utf-8"
    )
    print(f"\ncaptions for the built figures written to {captions_out}")

    if failed:
        raise SystemExit(1)


if __name__ == "__main__":
    main()
```

31 src/pipeline.py

```
from __future__ import annotations

import json
import time
import warnings
from pathlib import Path

warnings.filterwarnings("ignore")

ROOT = Path(__file__).resolve().parents[1]

import numpy as np
import pandas as pd
```

```python
from scipy import stats
from statsmodels.stats.multitest import multipletests

from src import survey
from src.audits import MODEL_FEATURES, leakage_audit, table1_sanity, vif_table
from src.calibration_dca import (
    brier_with_ci,
    calibrate_platt,
    calibration_table,
    decision_curve,
    pseudo_binary_outcome,
)
from src.cardiotoxicity import cardiotoxicity_group, e_value, interpret_e_value
from src.cohort import build_cohort
from src.config import N_JACKKNIFE_MAX_UNITS, RESULTS_METRICS, RESULTS_TABLES
from src.covariates import build_features
from src.finegray import design_ci_for_coefficients, fit_finegray_cox, hr_table
from src.gbm_competing_risk import design_ci_c_index, fit_gbm_cause_specific
from src.mi_sensitivity import MI_FEATURES, mi_trigger_note, run_mi_sensitivity
from src.negative_control import (
    CONTROL_FEATURES_FULL,
    CONTROL_FEATURES_MINIMAL,
    build_negative_control_frame,
    contrast_table,
)
from src.outcomes import (
    attach_outcomes,
    broad_cvd_outcome_summary,
    leading_cause_coding_by_cycle,
    outcome_summary,
)
from src.shap_analysis import (
    bootstrap_shap_rank_stability,
    global_shap,
    stratified_shap,
)
from src.stratify import site_strata, stratum_report, time_since_dx_strata
from src.table1 import build_table1

T0 = time.time()


def log(msg: str) -> None:
    print(f"[{time.time() - T0:7.1f}s] {msg}", flush=True)


def design_frame_of(df: pd.DataFrame) -> pd.DataFrame:
    return df.rename(
        columns={
            "psu": "SDMVPSU",
            "strata": "SDMVSTRA",
            "weight_mec": "WTMEC2YR",
        }
    )


def main() -> None:
    log("building cohort")
    cohort, inv = build_cohort()
    df = attach_outcomes(cohort)
    panel = build_features(df)
    panel["cardiotox_group"] = cardiotoxicity_group(panel)
    panel.to_parquet(
        ROOT / "data" / "processed" / "feature_panel_final.parquet",
        index=False,
    )
    log(f"cohort n={len(panel)}; inventory + outcomes saved")

    outc = outcome_summary(panel.rename(columns={}))
    outc.to_csv(RESULTS_TABLES / "outcome_summary.csv", index=False)
    print(inv.to_string())
    print(outc.to_string())

    coding = leading_cause_coding_by_cycle(df)
    coding.to_csv(
        RESULTS_TABLES / "leading_cause_coding_by_cycle.csv", index=False
```

```
    )
    cvd_defs = broad_cvd_outcome_summary(panel)
    cvd_defs.to_csv(RESULTS_TABLES / "cvd_definition_counts.csv", index=False)
    log("leading-cause coding by cycle:\n" + coding.to_string())
    log("cardiovascular-outcome definitions:\n" + cvd_defs.to_string())

    log("table1 sanity + design-weighted table1")
    problems = table1_sanity(panel)[1]
    (RESULTS_TABLES / "table1_sanity_problems.txt").write_text(
        "\n".join(problems) if problems else "no problems found"
    )
    log(f"table1 sanity problems: {problems if problems else 'NONE'}")
    build_table1(panel)

    log("leakage audit + VIF")
    leakage_audit()
    vif = vif_table(panel)
    log(f"max VIF = {vif['vif'].max():.2f}")

    log("MI sensitivity")
    pct_dropped, mi_note = mi_trigger_note(panel, MI_FEATURES)
    (RESULTS_TABLES / "mi_trigger.txt").write_text(mi_note)
    mi_cvd = run_mi_sensitivity(panel, cause=1)
    mi_cvd.to_csv(RESULTS_TABLES / "mi_sensitivity_cvd_death.csv", index=False)
    mi_cancer = run_mi_sensitivity(panel, cause=2)
    mi_cancer.to_csv(
        RESULTS_TABLES / "mi_sensitivity_cancer_death.csv", index=False
    )
    log(
        f"MI trigger: {pct_dropped:.1f}% dropped by listwise deletion on the 11-feature MI set"
    )

    log("Fine-Gray: fitting full-feature CVD and cancer models")
    cc = panel.dropna(
        subset=MODEL_FEATURES + ["followup_years", "event", "seqn"]
    ).copy()
    design_cc = survey.build(design_frame_of(cc), "mec", n_cycles=10)
    log(f"Fine-Gray complete-case n={len(cc)} (design n={design_cc.n})")

    ctv_cvd, ldf_cvd = fit_finegray_cox(
        cc,
        MODEL_FEATURES,
        "followup_years",
        "event",
        cause=1,
        design=design_cc,
        penalizer=0.5,
    )
    hr_cvd = hr_table(ctv_cvd)
    hr_cvd.to_csv(
        RESULTS_TABLES / "finegray_cvd_death_full_model.csv", index=False
    )

    ctv_cancer = fit_finegray_cox(
        cc,
        MODEL_FEATURES,
        "followup_years",
        "event",
        cause=2,
        design=design_cc,
        penalizer=0.5,
    )[0]
    hr_cancer = hr_table(ctv_cancer)
    hr_cancer.to_csv(
        RESULTS_TABLES / "finegray_cancer_death_full_model.csv", index=False
    )
    log("Fine-Gray full models fit")

    headline = [
        "age",
        "sex_female",
        "time_since_diagnosis_years",
        "hypertension",
        "diabetes",
        "crp",
        "bmi",
```

```
        "n_cancer_sites",
        "comorbidity_index",
        (
            "smoking_status_current"
            if "smoking_status_current" in ldf_cvd.columns
            else "smoking_status_never"
        ),
    ]
    log(
        f"Fine-Gray: design-aware jackknife CI for headline coefficients (CVD), "
        f"max_units={N_JACKKNIFE_MAX_UNITS}"
    )
    ci_cvd = design_ci_for_coefficients(
        cc,
        MODEL_FEATURES,
        "followup_years",
        "event",
        cause=1,
        design=design_cc,
        coefficients=headline,
        max_units=N_JACKKNIFE_MAX_UNITS,
        penalizer=0.5,
    )
    ci_cvd["narrow_ci_flag"] = (ci_cvd["HR_hi"] - ci_cvd["HR_lo"]) < 0.02
    ci_cvd.to_csv(
        RESULTS_TABLES / "finegray_cvd_death_design_ci_headline.csv",
        index=False,
    )
    log(
        f"CVD headline CI done; any suspiciously narrow? {ci_cvd['narrow_ci_flag'].any()}"
    )

    log(
        "Fine-Gray: design-aware jackknife CI for headline coefficients (cancer)"
    )
    ci_cancer = design_ci_for_coefficients(
        cc,
        MODEL_FEATURES,
        "followup_years",
        "event",
        cause=2,
        design=design_cc,
        coefficients=headline,
        max_units=N_JACKKNIFE_MAX_UNITS,
        penalizer=0.5,
    )
    ci_cancer["narrow_ci_flag"] = (
        ci_cancer["HR_hi"] - ci_cancer["HR_lo"]
    ) < 0.02
    ci_cancer.to_csv(
        RESULTS_TABLES / "finegray_cancer_death_design_ci_headline.csv",
        index=False,
    )

    def design_holm(ci: pd.DataFrame) -> pd.DataFrame:
        d = ci.set_index("feature").loc[headline]
        df_resid = d["df"].to_numpy()
        t_crit = stats.t.ppf(0.975, df_resid)
        se = (d["coef_hi"].to_numpy() - d["coef_lo"].to_numpy()) / (
            2.0 * t_crit
        )
        t_stat = d["coef"].to_numpy() / se
        p_raw = 2.0 * stats.t.sf(np.abs(t_stat), df_resid)
        reject, p_adj, _, _ = multipletests(p_raw, alpha=0.05, method="holm")
        return pd.DataFrame(
            {
                "feature": headline,
                "coef": d["coef"].to_numpy(),
                "se_design": se,
                "df": df_resid,
                "t_stat": t_stat,
                "p_raw_design": p_raw,
                "p_adj_design": p_adj,
                "reject_h0": reject,
            }
        )
```

```
    design_holm(ci_cvd).to_csv(
        RESULTS_TABLES / "holm_bonferroni_cvd_headline.csv", index=False
    )
    design_holm(ci_cancer).to_csv(
        RESULTS_TABLES / "holm_bonferroni_cancer_headline.csv", index=False
    )
    log("Holm-Bonferroni (design-based) written for both causes")

    parsimonious = [
        "age",
        "sex_female",
        "time_since_diagnosis_years",
        "hypertension",
        "diabetes",
        "crp",
        "bmi",
        "n_cancer_sites",
        "comorbidity_index",
        "smoking_status",
    ]
    for cause, name in ((1, "cvd"), (2, "cancer")):
        ctv_p, _ = fit_finegray_cox(
            cc,
            parsimonious,
            "followup_years",
            "event",
            cause=cause,
            design=design_cc,
            penalizer=0.5,
        )
        log(f"Fine-Gray parsimonious ({name}): design-aware jackknife CI")
        ci_p = design_ci_for_coefficients(
            cc,
            parsimonious,
            "followup_years",
            "event",
            cause=cause,
            design=design_cc,
            coefficients=list(ctv_p.summary.index),
            max_units=N_JACKKNIFE_MAX_UNITS,
            penalizer=0.5,
        )
        ci_p.to_csv(
            RESULTS_TABLES
            / f"finegray_{name}_death_design_ci_parsimonious.csv",
            index=False,
        )
        design_holm(ci_p).to_csv(
            RESULTS_TABLES / f"holm_bonferroni_{name}_parsimonious.csv",
            index=False,
        )
    log("parsimonious Fine-Gray models written for both causes")

    log(
        "cardiovascular outcome: heart-or-cerebrovascular sensitivity, 1999-2014"
    )
    cc_broad = cc[cc["leading_cause_detailed_cycle"].astype(bool)].copy()
    design_broad = survey.build(design_frame_of(cc_broad), "mec", n_cycles=8)
    log(
        f"broad-definition subset n={len(cc_broad)} "
        f"({int((cc_broad['event_broad_cvd'] == 1).sum())} cardiovascular deaths, "
        f"{int((cc_broad['event'] == 1).sum())} under the primary definition)"
    )
    ci_broad = design_ci_for_coefficients(
        cc_broad,
        MODEL_FEATURES,
        "followup_years",
        "event_broad_cvd",
        cause=1,
        design=design_broad,
        coefficients=headline,
        max_units=N_JACKKNIFE_MAX_UNITS,
        penalizer=0.5,
    )
    ci_broad.to_csv(
```

```python
        RESULTS_TABLES / "finegray_cvd_death_design_ci_broad_definition.csv",
        index=False,
    )
    ci_primary_detailed = design_ci_for_coefficients(
        cc_broad,
        MODEL_FEATURES,
        "followup_years",
        "event",
        cause=1,
        design=design_broad,
        coefficients=headline,
        max_units=N_JACKKNIFE_MAX_UNITS,
        penalizer=0.5,
    )
    ci_primary_detailed.to_csv(
        RESULTS_TABLES / "finegray_cvd_death_design_ci_detailed_cycles.csv",
        index=False,
    )
    log("broad-definition sensitivity written")

    pen_rows = []
    for cause, name, ctv_main in (
        (1, "cvd", ctv_cvd),
        (2, "cancer", ctv_cancer),
    ):
        ctv_lo, _ = fit_finegray_cox(
            cc,
            MODEL_FEATURES,
            "followup_years",
            "event",
            cause=cause,
            design=design_cc,
            penalizer=0.01,
        )
        for f in headline:
            hr_main = float(np.exp(ctv_main.summary.loc[f, "coef"]))
            hr_lo = float(np.exp(ctv_lo.summary.loc[f, "coef"]))
            pen_rows.append(
                {
                    "cause": name,
                    "feature": f,
                    "HR_penalizer_0.5": hr_main,
                    "HR_penalizer_0.01": hr_lo,
                    "abs_log_hr_diff": abs(np.log(hr_main) - np.log(hr_lo)),
                }
            )
    pd.DataFrame(pen_rows).to_csv(
        RESULTS_TABLES / "finegray_penalty_sensitivity.csv", index=False
    )
    log("ridge-penalty sensitivity written")

    log(
        "cardiotoxicity contrast: high vs low expected cardiotoxicity site groups"
    )
    ct_features = [f for f in MODEL_FEATURES if f != "n_cancer_sites"] + [
        "cardiotox_high"
    ]
    cc_ct = panel[
        panel["cardiotox_group"].isin(
            ["High expected cardiotoxicity", "Low expected cardiotoxicity"]
        )
    ].copy()
    cc_ct["cardiotox_high"] = (
        cc_ct["cardiotox_group"] == "High expected cardiotoxicity"
    )
    cc_ct = cc_ct.dropna(
        subset=ct_features + ["followup_years", "event", "seqn"]
    )
    log(
        f"cardiotoxicity high-vs-low complete-case n={len(cc_ct)} "
        f"(high={int(cc_ct['cardiotox_high'].sum())}, low={int((~cc_ct['cardiotox_high']).sum())})"
    )
    design_ct = survey.build(design_frame_of(cc_ct), "mec", n_cycles=10)

    ctv_ct, _ = fit_finegray_cox(
        cc_ct,
```

```
        ct_features,
        "followup_years",
        "event",
        cause=1,
        design=design_ct,
        penalizer=0.5,
    )
    hr_ct = hr_table(ctv_ct)
    hr_ct.to_csv(
        RESULTS_TABLES / "cardiotoxicity_cvd_death_model.csv", index=False
    )

    ci_ct = design_ci_for_coefficients(
        cc_ct,
        ct_features,
        "followup_years",
        "event",
        cause=1,
        design=design_ct,
        coefficients=(
            ["cardiotox_high_True"]
            if "cardiotox_high_True" in ctv_ct.summary.index
            else [c for c in ctv_ct.summary.index if "cardiotox" in c]
        ),
        max_units=N_JACKKNIFE_MAX_UNITS,
        penalizer=0.5,
    )
    ci_ct.to_csv(
        RESULTS_TABLES / "cardiotoxicity_cvd_death_design_ci.csv", index=False
    )
    log(f"cardiotoxicity HR (design CI): {ci_ct.to_dict('records')}")

    if len(ci_ct):
        row = ci_ct.iloc[0]
        ci_bound = row["HR_lo"] if row["HR"] >= 1 else row["HR_hi"]
        ev = e_value(row["HR"], ci_bound)
        ev_text = interpret_e_value(
            ev["e_value_point"], ev.get("e_value_ci"), row["HR"]
        )
        (RESULTS_TABLES / "cardiotoxicity_e_value.txt").write_text(
            json.dumps(ev, indent=2) + "\n\n" + ev_text
        )
        log(f"E-value: {ev}")

    log(
        "late-onset probe: cardiotox_high x time_since_diagnosis_years interaction"
    )
    cc_ct2 = cc_ct.copy()
    cc_ct2["cardiotox_x_time"] = (
        cc_ct2["cardiotox_high"].astype(float)
        * cc_ct2["time_since_diagnosis_years"]
    )
    ct2_features = ct_features + ["cardiotox_x_time"]
    ctv_ct2, _ = fit_finegray_cox(
        cc_ct2,
        ct2_features,
        "followup_years",
        "event",
        cause=1,
        design=design_ct,
        penalizer=0.5,
    )
    hr_ct2 = hr_table(ctv_ct2)
    hr_ct2.to_csv(
        RESULTS_TABLES / "cardiotoxicity_time_interaction_model.csv",
        index=False,
    )

    log("stratification: time since diagnosis and site")
    tsd_strata = time_since_dx_strata(panel)
    stratum_report(
        panel,
        tsd_strata,
        "time_since_diagnosis",
        shap_features=list(MODEL_FEATURES),
    )
```

```python
    tsd_rates = []
    for label, mask in tsd_strata.items():
        sub = panel.loc[mask.reindex(panel.index).fillna(False)]
        if len(sub) < 10:
            tsd_rates.append(
                {
                    "stratum": label,
                    "n": len(sub),
                    "cvd_pct": np.nan,
                    "cancer_pct": np.nan,
                }
            )
            continue
        d = survey.build(design_frame_of(sub), "mec", n_cycles=10)
        cvd_p = survey.wprop(
            sub.loc[d.weight.index, "cvd_death"].astype(bool), d
        )
        cancer_p = survey.wprop(
            sub.loc[d.weight.index, "cancer_death"].astype(bool), d
        )
        tsd_rates.append(
            {
                "stratum": label,
                "n": len(sub),
                "cvd_pct": round(100 * cvd_p, 1),
                "cancer_pct": round(100 * cancer_p, 1),
            }
        )
    pd.DataFrame(tsd_rates).to_csv(
        RESULTS_TABLES / "time_since_diagnosis_cvd_vs_cancer_rates.csv",
        index=False,
    )

    site_strata_d = site_strata(panel)
    site_report = stratum_report(
        panel, site_strata_d, "cancer_site", shap_features=list(MODEL_FEATURES)
    )
    log(
        f"site strata supporting a model (n>=100): "
        f"{site_report.loc[site_report.supports_model, 'stratum'].tolist()}"
    )

    log("GBM cause-specific ensembles")
    oof_cvd, model_cvd = fit_gbm_cause_specific(panel, MODEL_FEATURES, cause=1)
    oof_cancer, model_cancer = fit_gbm_cause_specific(
        panel, MODEL_FEATURES, cause=2
    )

    cidx_cvd = design_ci_c_index(
        panel,
        oof_cvd,
        cause=1,
        design_frame_cols={
            "psu": "psu",
            "strata": "strata",
            "weight": "weight_mec",
        },
        n_cycles=10,
        max_units=N_JACKKNIFE_MAX_UNITS,
    )
    cidx_cancer = design_ci_c_index(
        panel,
        oof_cancer,
        cause=2,
        design_frame_cols={
            "psu": "psu",
            "strata": "strata",
            "weight": "weight_mec",
        },
        n_cycles=10,
        max_units=N_JACKKNIFE_MAX_UNITS,
    )
    pd.DataFrame(
        [
            {"model": "cvd_death", **cidx_cvd},
            {"model": "cancer_death", **cidx_cancer},
```

```
        ]
    ).to_csv(RESULTS_METRICS / "gbm_c_index_design_ci.csv", index=False)
    log(f"C-index CVD: {cidx_cvd}; cancer: {cidx_cancer}")

    Xg = panel.dropna(subset=MODEL_FEATURES).copy()
    from src.gbm_competing_risk import _encode

    Xg_enc = _encode(Xg[MODEL_FEATURES])
    shap_cvd = global_shap(model_cvd, Xg_enc, tag="cvd_death")
    global_shap(model_cancer, Xg_enc, tag="cancer_death")
    log("global SHAP:\n" + shap_cvd.head(10).to_string())

    rank_stability_cvd = bootstrap_shap_rank_stability(
        model_cvd, Xg_enc, n_boot=30
    )
    rank_stability_cancer = bootstrap_shap_rank_stability(
        model_cancer, Xg_enc, n_boot=30
    )
    (RESULTS_METRICS / "shap_rank_stability.json").write_text(
        json.dumps(
            {
                "cvd_death_spearman": rank_stability_cvd,
                "cancer_death_spearman": rank_stability_cancer,
            },
            indent=2,
        )
    )
    log(
        f"SHAP rank stability: CVD={rank_stability_cvd:.3f}, cancer={rank_stability_cancer:.3f}"
    )

    tsd_strata_x = {
        k: v.reindex(Xg_enc.index).fillna(False) for k, v in tsd_strata.items()
    }
    site_strata_x = {
        k: v.reindex(Xg_enc.index).fillna(False)
        for k, v in site_strata_d.items()
    }
    _, tsd_shap_n = stratified_shap(
        model_cvd, Xg_enc, tsd_strata_x, tag="cvd_by_time_since_dx"
    )
    _, site_shap_n = stratified_shap(
        model_cvd, Xg_enc, site_strata_x, tag="cvd_by_site"
    )
    log("SHAP stratum n (time-since-dx):\n" + tsd_shap_n.to_string())
    log("SHAP stratum n (site):\n" + site_shap_n.to_string())

    log("calibration + DCA (CVD death, 10-year horizon)")
    cal_cycles = [
        "1999-2000",
        "2001-2002",
        "2003-2004",
        "2005-2006",
        "2007-2008",
    ]
    idx = oof_cvd.dropna().index
    idx = idx[panel.loc[idx, "cycle"].isin(cal_cycles).to_numpy()]
    time_arr = panel.loc[idx, "followup_years"].to_numpy()
    event_arr = panel.loc[idx, "event"].to_numpy()
    eligible, y10 = pseudo_binary_outcome(
        time_arr, event_arr, cause=1, horizon=10.0
    )
    risk_elig = oof_cvd.loc[idx].to_numpy()[eligible]
    y10_elig = y10[eligible]
    log(
        f"calibration/DCA evaluable n={eligible.sum()} of {len(idx)} in 1999-2008 "
        f"(dropped: censored or competing-event before 10y, true 10y status unknown)"
    )
    pd.DataFrame(
        [
            {
                "cycles": "1999-2008",
                "n_complete_case_in_cycles": len(idx),
                "n_evaluable": int(eligible.sum()),
                "n_cvd_by_10y": int(y10[eligible].sum()),
                "min_followup_censored_years": float(
```

```python
                    time_arr[event_arr == 0].min()
                ),
            }
        ]
    ).to_csv(RESULTS_TABLES / "calibration_sample_cvd_10y.csv", index=False)
    p_cal, _ = calibrate_platt(risk_elig, y10_elig)
    cal_table = calibration_table(p_cal, y10_elig, n_bins=10)
    cal_table.to_csv(
        RESULTS_TABLES / "calibration_table_cvd_10y.csv", index=False
    )
    brier = brier_with_ci(p_cal, y10_elig)
    (RESULTS_METRICS / "brier_cvd_10y.json").write_text(
        json.dumps(brier, indent=2)
    )
    dca = decision_curve(p_cal, y10_elig)
    dca.to_csv(RESULTS_TABLES / "decision_curve_cvd_10y.csv", index=False)
    log(f"Brier (10y CVD): {brier}")

    log(
        "control analyses: negative-control outcome and negative-control exposure"
    )
    nc_frame = build_negative_control_frame()
    log(
        "control population: "
        + str(nc_frame["cancer_group"].value_counts().to_dict())
    )
    contrast_rows = []
    for outcome, detailed in (
        ("accidental_death", True),
        ("cardiovascular_death", False),
    ):
        for features, label in (
            (CONTROL_FEATURES_MINIMAL, "age and sex"),
            (CONTROL_FEATURES_FULL, "full covariate set"),
        ):
            table = contrast_table(
                nc_frame,
                outcome,
                features,
                restrict_to_detailed_cycles=detailed,
                max_units=N_JACKKNIFE_MAX_UNITS,
                n_cycles=8 if detailed else 10,
            )
            table["adjustment_label"] = label
            contrast_rows.append(table)
            log(f"{outcome}, {label}:\n" + table.to_string())
    contrasts = pd.concat(contrast_rows, ignore_index=True)
    contrasts.to_csv(RESULTS_TABLES / "control_contrasts.csv", index=False)

    primary = contrasts[
        (contrasts["outcome"] == "accidental_death")
        & (contrasts["adjustment_label"] == "full covariate set")
        & (contrasts["contrast"] == "survivor_vs_no_cancer")
    ].iloc[0]
    nc_res = {
        "outcome": "accidental_death (negative control)",
        "adjustment": "full covariate set",
        "coef": float(primary["coef"]),
        "coef_lo": float(primary["coef_lo"]),
        "coef_hi": float(primary["coef_hi"]),
        "HR": float(primary["HR"]),
        "HR_lo": float(primary["HR_lo"]),
        "HR_hi": float(primary["HR_hi"]),
        "n_total": int(primary["n_analysed"]),
        "n_cancer_survivors": int(primary["n_survivors"]),
        "n_skin_cancer_only": int(primary["n_skin_only"]),
        "n_accidental_deaths": int(primary["n_events"]),
    }
    (RESULTS_TABLES / "negative_control_accidental_death.json").write_text(
        json.dumps(nc_res, indent=2)
    )
    log(f"negative control result: {nc_res}")

    claim_ceiling = f"""SCOPE OF CLAIMS AND LIMITATIONS.

NHANES records no cancer TREATMENT (chemotherapy, anthracyclines, trastuzumab,
```

```
radiotherapy field, androgen-deprivation therapy). This paper may report that
BASELINE CHARACTERISTICS (age, sex, comorbidity burden, inflammatory markers,
time since diagnosis, expected treatment cardiotoxicity of the reported site) are
ASSOCIATED WITH the relative hazard of cardiovascular versus cancer death among
survivors. It must NOT claim that cancer TREATMENT causes cardiovascular death --
treatment exposure is never observed.

Cancer diagnosis, site, and age at diagnosis are all SELF-REPORTED (NHANES MCQ
items), with no registry (SEER, tumor board, pathology) confirmation of site,
stage, or histology. Site-level misclassification is possible and not
quantifiable from these data.

NEGATIVE CONTROL RESULT (cycles 1999-2014, where accidental death is coded): cancer survivors have a HIGHER hazard of accidental
(external-cause) death than non-survivors of the same age and sex
(HR {nc_res['HR']:.2f}, 95% CI {nc_res['HR_lo']:.2f}-{nc_res['HR_hi']:.2f}, n={nc_res['n_total']:,},
{nc_res['n_accidental_deaths']} accidental deaths) -- an outcome with no plausible
mechanistic link to cancer or its treatment. This means the survivor-vs-non-
survivor contrast in this dataset partly reflects GENERAL FRAILTY, comorbidity,
or differential healthcare/surveillance contact, not a cancer- or treatment-
specific pathway. Every survivor-population result in this paper should be read
with that caveat; it does not by itself invalidate the WITHIN-SURVIVOR
cardiotoxicity-group contrast (which does not compare survivors to non-
survivors), but it is a reason for caution about any implied specificity.

CARDIOTOXICITY CONTRAST: the high-vs-low expected-cardiotoxicity site grouping is
an INDIRECT, SITE-LEVEL, ECOLOGICAL proxy for an individual-level exposure
(treatment) that was never observed. Report it as SUGGESTIVE, not confirmatory.
Prostate's inclusion in the high-cardiotoxicity group is the weakest of the three
members (androgen-deprivation therapy is common but not universal for prostate
cancer); state this explicitly wherever prostate-driven results are discussed.
"""
    (RESULTS_TABLES / "claim_ceiling_and_limitations.txt").write_text(
        claim_ceiling
    )

    log(
        "comparing jackknife CIs against the pre-fix (biased leading-strata) run"
    )
    old_dir = ROOT / "results" / "_v1_biased_maxunits30"
    if old_dir.exists():
        compare_rows = []
        pairs = [
            ("finegray_cvd_death_design_ci_headline.csv", ci_cvd, "feature"),
            (
                "finegray_cancer_death_design_ci_headline.csv",
                ci_cancer,
                "feature",
            ),
            ("cardiotoxicity_cvd_death_design_ci.csv", ci_ct, "feature"),
        ]
        for fname, new_df, key in pairs:
            old_path = old_dir / fname
            if not old_path.exists():
                continue
            old_df = pd.read_csv(old_path)
            m = old_df.merge(
                new_df, on=key, suffixes=("_old_biased", "_new_fixed")
            )
            for _, r in m.iterrows():
                compare_rows.append(
                    {
                        "source": fname,
                        "feature": r[key],
                        "HR_old_biased": r.get("HR_old_biased"),
                        "HR_new_fixed": r.get("HR_new_fixed"),
                        "HR_lo_old_biased": r.get("HR_lo_old_biased"),
                        "HR_lo_new_fixed": r.get("HR_lo_new_fixed"),
                        "HR_hi_old_biased": r.get("HR_hi_old_biased"),
                        "HR_hi_new_fixed": r.get("HR_hi_new_fixed"),
                        "n_units_old_biased": r.get(
                            "n_jackknife_units_old_biased"
                        ),
                        "n_units_new_fixed": r.get(
                            "n_jackknife_units_new_fixed"
                        ),
```

```
                    }
                )
        if compare_rows:
            cmp_df = pd.DataFrame(compare_rows)
            cmp_df.to_csv(
                RESULTS_TABLES / "jackknife_fix_old_vs_new_comparison.csv",
                index=False,
            )
            log(
                "old (biased, max_units=30, leading strata) vs new (fixed, evenly-spaced, "
                f"max_units={N_JACKKNIFE_MAX_UNITS}) comparison:\n"
                + cmp_df.to_string()
            )

    log("DONE")


if __name__ == "__main__":
    main()
```

32 scripts/run_all.py

```
from __future__ import annotations

import subprocess
import sys
from pathlib import Path

ROOT = Path(__file__).resolve().parents[1]

STEPS: list[tuple[str, list[str]]] = [
    ("cohort, models, controls", ["-m", "src.pipeline"]),
    ("condition answer codes", ["-m", "src.condition_answer_codes"]),
    (
        "delayed entry on the diagnosis time scale",
        ["-m", "src.left_truncation"],
    ),
    ("relative survival", ["-m", "src.relative_survival"]),
    ("design-based intervals", ["-m", "src.design_based_intervals"]),
    (
        "simple-baseline benchmarks",
        ["-m", "src.baseline_models", "--design-ci"],
    ),
    ("C-reactive-protein sensitivity", ["-m", "src.crp_sensitivity"]),
    ("figures", ["-m", "src.make_figures"]),
]


def main() -> None:
    requested = sys.argv[1:]
    for label, command in STEPS:
        if requested and command[1] not in requested:
            continue
        print(f"=== {label} ({command[1]}) ===", flush=True)
        result = subprocess.run(
            [sys.executable, *command], cwd=str(ROOT), check=False
        )
        if result.returncode != 0:
            print(
                f"=== failed: {label} (exit {result.returncode}) ===",
                flush=True,
            )
            sys.exit(result.returncode)
    print("=== all steps complete ===", flush=True)


if __name__ == "__main__":
    main()
```
