# Supplementary material for: Negative-Control Calibration and E-Value Bounding of Cancer-Survivor Mortality Contrasts: A Design-Aware Competing-Risks Analysis
Source: Cancers (Basel). 2026 Nov 4;18(21):3544. doi: 10.3390/cancers18213544 (PMC13762343; doi:10.3390/cancers18213544)
Supplement: Supplementary file 1 [file cancers-18-03544-s001.zip › Supplementary File S2.pdf]

Supplementary Tables

Table S1. Sequential subsample inventory for the NHANES 1999–2018 cancer-survivor cohort.

Step	n	Definition
NHANES 1999-2018, DEMO merged with MCQ on SEQN	101,316	full population, both sexes, all ages
Adults >=20 y (all cycles)	55,081	RIDAGEYR >= 20, interview-eligible
Adults >=20 y with a valid MEC exam weight	52,398	WTMEC2YR > 0 (examined subsample; required for the lab/anthropometric panel)
… reporting a physician diagnosis of cancer (MCQ220 = 1)	4864	self-reported 'ever told you had cancer or malignancy'
… with an identifiable cancer site (MCQ230/MCQ240 items)	4830	site recoverable from the harmonized MCQ230/MCQ240 (or MCD240) items
Excluding survivors whose only reported cancer is skin, non-melanoma or unknown histology (MCQ240W/X or code 32/33)	1038	removed: registry convention treats non-melanoma/unspecified skin cancer as a distinct, much less lethal entity that is excluded from most cancer-survivorship cohorts and from SEER incidence counts
… with an implausible age at diagnosis (> current age; dropped to missing)	0	age_at_diagnosis > RIDAGEYR at the same or a later cycle; set to missing rather than excluding the person
Survivor cohort with a computable time since diagnosis	3773	age at MEC exam minus valid age at diagnosis
FINAL analytic survivor cohort (n_survivor)	3826	all cancer-diagnosis-reporting, MEC-examined adults >=20y, skin-cancer-only cases excluded; site and/or time-since-diagnosis may be missing for a documented subset

Table S1 note. The identifiable-cancer-site and computable-time-since-diagnosis rows describe field completeness within the cohort rather than exclusions.

Table S2. NCHS leading-cause-of-death recode by survey cycle among decedents of the survivor cohort.

Cycle	Deaths	Diseases of heart (001)	Cerebrovascular (005)	Malignant neoplasms (002)	Accidents (004)	Residual (010)
1999-2000	196	39	5	69	4	43
2001-2002	221	37	16	76	5	49
2003-2004	197	50	11	61	4	31
2005-2006	155	32	6	46	5	39
2007-2008	186	37	7	69	2	49
2009-2010	164	38	8	56	9	32
2011-2012	105	21	3	37	6	25
2013-2014	90	16	7	35	2	18
2015-2016	65	9	0	32	0	24
2017-2018	37	9	0	16	0	12

Table S2 note. In 2015–2016 and 2017–2018 only codes 001, 002, and the residual category 010 carry deaths, so cerebrovascular and accidental death cannot be identified in those two cycles.

Table S3. Effect of the cardiovascular-death definition: event counts under each definition, and the headline covariates of the fully adjusted model under both definitions on the eight cycles in which cerebrovascular death is coded separately.

Cardiovascular-death definition	Cohort n	Cardiovascular deaths	Cancer deaths	Other deaths	Censored
all ten cycles, diseases of heart	3826	288	497	631	2410
all ten cycles, heart or cerebrovascular	3826	351	497	568	2410
cycles 1999-2014, diseases of heart	2953	270	449	595	1639
cycles 1999-2014, heart or cerebrovascular	2953	333	449	532	1639

Covariate	Diseases of heart, 1999–2014	Heart or cerebrovascular, 1999–2014
Age (per year)	1.08 (1.06–1.10)	1.08 (1.06–1.10)
Female sex	0.77 (0.51–1.16)	0.75 (0.51–1.10)
Time since diagnosis (per year)	1.00 (0.98–1.01)	1.00 (0.99–1.01)
Hypertension	0.80 (0.50–1.27)	0.80 (0.52–1.21)
Diabetes	0.92 (0.49–1.73)	0.93 (0.55–1.58)
C-reactive protein	1.00 (0.98–1.03)	1.00 (0.97–1.02)
BMI	0.99 (0.95–1.04)	0.98 (0.94–1.02)
Number of cancer sites	0.97 (0.60–1.56)	1.01 (0.68–1.51)
Comorbidity index	1.39 (1.21–1.60)	1.38 (1.22–1.56)
Smoking: never (ref. current)	0.71 (0.38–1.32)	0.86 (0.49–1.50)

Table S3 note. Hazard ratios with design-aware jackknife 95% confidence intervals. The two columns differ only in which deaths count as the event of interest.

Table S4. Negative-control outcome and negative-control exposure in the examined adult population: both exposure indicators entered together with participants reporting no cancer as the reference, under two adjustment sets.

Outcome	Adjustment	Contrast	HR (95% CI)	n	Events
Accidental death	age and sex	Survivor cohort vs. no reported cancer	1.77 (0.98–3.19)	41,544	250
Accidental death	age and sex	Skin cancer only vs. no reported cancer	1.59 (0.72–3.49)	41,544	250
Accidental death	age and sex	Calibrated: survivor minus skin cancer only	1.12 (0.44–2.83)	41,544	250
Accidental death	full covariate set	Survivor cohort vs. no reported cancer	1.78 (0.89–3.54)	34,141	199
Accidental death	full covariate set	Skin cancer only vs. no reported cancer	2.01 (0.87–4.66)	34,141	199
Accidental death	full covariate set	Calibrated: survivor minus skin cancer only	0.88 (0.33–2.33)	34,141	199
Cardiovascular death	age and sex	Survivor cohort vs. no reported cancer	0.92 (0.79–1.09)	52,224	2126
Cardiovascular death	age and sex	Skin cancer only vs. no reported cancer	0.81 (0.62–1.06)	52,224	2126
Cardiovascular death	age and sex	Calibrated: survivor minus skin cancer only	1.14 (0.84–1.55)	52,224	2126
Cardiovascular death	full covariate set	Survivor cohort vs. no reported cancer	0.84 (0.71–1.00)	42,728	1700
Cardiovascular death	full covariate set	Skin cancer only vs. no reported cancer	0.87 (0.65–1.18)	42,728	1700
Cardiovascular death	full covariate set	Calibrated: survivor minus skin cancer only	0.96 (0.70–1.32)	42,728	1700

Table S4 note. The calibrated row is the difference of the two log hazard ratios, computed inside every jackknife replicate. Accidental death is restricted to the eight cycles that code it separately (Table S2).

Table S5. Target-leakage pre-flight audit: every covariate screened for an algebraic or definitional relationship to the outcome, with the decision and its reason.

Covariate	Status and reason
age_at_diagnosis	Algebraically redundant with age and time_since_diagnosis_years (age = age_at_diagnosis + time_since_diagnosis_years exactly); including all three gives infinite VIF
followup_years	The survival TIME variable itself – using it as a predictor of the event that defines it is direct leakage
MORTSTAT	Identical information to the event/outcome columns
UCOD_LEADING	The raw cause-of-death code the outcome is derived from
cv_death	Component of the outcome definition
cancer_death_flag_raw	Component of the outcome definition (note: ‘cancer_death’ the LMF flag and ‘cancer_death’ the outcome column share a name pre-merge in outcomes.py; the LMF flag is dropped, only the outcome column survives under that name)
site_group	Categorical version of the site information; used for STRATIFICATION, not as a model feature, to avoid a 10+ level one-hot block dominating a cohort of n<4000
site_name	Same reason as site_group, at even higher cardinality
n_cancer_sites	Kept – reviewed and retained: a count of reported diagnoses is not definitionally downstream of which cause a person eventually dies of

Table S6. Fully adjusted survey-weighted Fine–Gray subdistribution-hazard model for cardiovascular death and cancer death (32 coefficients; race/ethnicity and education entered as indicator variables), with design-aware jackknife 95% confidence intervals (238 jackknife units, 118 strata, 120 degrees of freedom; complete-case $n = 2225$).

Covariate	CVD death HR (95% CI)	Cancer death HR (95% CI)
Age (per year)	1.08 (1.06–1.10)	1.03 (1.01–1.05)
Female sex	0.74 (0.50–1.08)	0.50 (0.34–0.74)
Income-to-poverty ratio	0.86 (0.74–1.00)	0.97 (0.87–1.09)
BMI	0.99 (0.95–1.03)	1.00 (0.96–1.03)
Systolic BP	1.01 (1.00–1.02)	1.00 (0.99–1.01)
Diastolic BP	1.00 (0.99–1.01)	1.00 (0.99–1.01)
Total cholesterol	1.00 (1.00–1.01)	1.00 (1.00–1.00)
HDL cholesterol	1.00 (0.98–1.01)	1.00 (0.99–1.01)
HbA1c	0.99 (0.78–1.25)	0.98 (0.72–1.33)
C-reactive protein	1.00 (0.98–1.02)	1.02 (1.00–1.04)
Neutrophil-to-lymphocyte ratio	1.09 (0.97–1.22)	0.99 (0.89–1.09)
Hemoglobin	0.97 (0.88–1.08)	0.85 (0.74–0.98)
Serum albumin	0.78 (0.42–1.47)	1.34 (0.72–2.48)
Hypertension	0.81 (0.51–1.28)	1.17 (0.81–1.70)
Diabetes	0.89 (0.52–1.54)	1.06 (0.63–1.78)
Comorbidity index	1.40 (1.24–1.59)	0.89 (0.80–1.00)
Healthcare visits, past 12 mo (ordinal category)	0.96 (0.84–1.11)	1.16 (1.04–1.29)
Self-rated general health	1.11 (0.93–1.33)	0.97 (0.83–1.14)
Time since diagnosis (per year)	1.00 (0.99–1.01)	0.99 (0.98–1.00)
Number of cancer sites	0.92 (0.59–1.45)	2.18 (1.54–3.08)
Race/ethnicity: Other Hispanic (ref. Mexican American)	0.33 (0.07–1.52)	0.78 (0.35–1.74)
Race/ethnicity: Non-Hispanic White (ref. Mexican American)	1.54 (1.03–2.30)	0.86 (0.58–1.27)
Race/ethnicity: Non-Hispanic Black (ref. Mexican American)	1.33 (0.81–2.18)	1.52 (0.92–2.49)
Race/ethnicity: Other or multiracial (ref. Mexican American)	0.32 (0.07–1.49)	0.45 (0.16–1.27)
Education: 9th-11th grade (ref. less than 9th grade)	0.73 (0.39–1.38)	1.44 (0.90–2.30)
Education: High school or GED (ref. less than 9th grade)	0.99 (0.60–1.63)	1.22 (0.83–1.80)
Education: Some college (ref. less than 9th grade)	1.03 (0.61–1.74)	1.12 (0.74–1.68)
Education: College graduate or above (ref. less than 9th grade)	1.04 (0.60–1.81)	0.99 (0.61–1.58)
Smoking: former (ref. current)	0.78 (0.46–1.31)	0.66 (0.41–1.07)
Smoking: never (ref. current)	0.74 (0.44–1.26)	0.48 (0.32–0.72)
Alcohol, ≥12 drinks in any year	0.90 (0.65–1.26)	0.96 (0.62–1.48)
Physically active	0.98 (0.63–1.51)	0.75 (0.52–1.08)

Table S7. Delayed-entry analysis on the time-since-diagnosis scale: subsets analyzed, and the distribution of entry times, exit times, and inverse-probability weights.

Subset	n	Cardiovascular deaths	Cancer deaths	Other deaths	Median years since diagnosis
survivor cohort	3826	288	497	631	8.0
with a computable time since diagnosis	3771	281	493	624	8.0
covariate-complete, delayed entry	2224	173	277	385	8.0
examined within 5 years of diagnosis	813	68	120	134	2.0
examined within 2 years of diagnosis	412	41	60	72	1.0

Quantity	25th percentile	Median	75th percentile	Maximum
entry time (years since diagnosis)	4.00	8.00	16.00	78.00
exit time (years since diagnosis)	11.40	17.70	26.00	93.00
inverse-probability weight in the risk-set extension	0.67	0.96	1.26	12.09

Table S8. Observed, expected, and net survival among survivors against expected mortality modeled internally in the examined adults with no reported cancer ($n = 43{,}025$), survivor cohort $n = 3473$.

Expected-mortality model	Horizon	Quantity	Estimate (95% CI)
demographic	5 y	Observed all-cause survival	0.849 (0.835–0.863)
demographic	5 y	Expected survival	0.896 (0.892–0.901)
demographic	5 y	Relative survival (observed / expected)	0.947 (0.933–0.961)
demographic	5 y	Net survival (Pohar-Perme)	0.945 (0.930–0.960)
demographic	5 y	Recorded cancer-death incidence	0.068 (0.059–0.077)
demographic	10 y	Observed all-cause survival	0.701 (0.682–0.720)
demographic	10 y	Expected survival	0.771 (0.761–0.780)
demographic	10 y	Relative survival (observed / expected)	0.909 (0.889–0.930)
demographic	10 y	Net survival (Pohar-Perme)	0.897 (0.869–0.924)
demographic	10 y	Recorded cancer-death incidence	0.115 (0.102–0.127)
demographic and baseline clinical	5 y	Observed all-cause survival	0.855 (0.842–0.868)
demographic and baseline clinical	5 y	Expected survival	0.899 (0.894–0.904)
demographic and baseline clinical	5 y	Relative survival (observed / expected)	0.951 (0.938–0.965)
demographic and baseline clinical	5 y	Net survival (Pohar-Perme)	0.954 (0.939–0.970)
demographic and baseline clinical	5 y	Recorded cancer-death incidence	0.066 (0.057–0.075)
demographic and baseline clinical	10 y	Observed all-cause survival	0.711 (0.692–0.729)
demographic and baseline clinical	10 y	Expected survival	0.776 (0.767–0.786)
demographic and baseline clinical	10 y	Relative survival (observed / expected)	0.916 (0.896–0.936)

Expected-mortality model	Horizon	Quantity	Estimate (95% CI)
demographic and baseline clinical	10 y	Net survival (Pohar-Perme)	0.947 (0.877–1.016)
demographic and baseline clinical	10 y	Recorded cancer-death incidence	0.112 (0.099–0.125)

Table S8 note. Expected mortality is treated as known, so the intervals carry sampling variability in the survivor cohort only. The recorded cancer-death incidence row is the only quantity here that uses cause of death.

Table S9. Calibration by risk decile and decision-curve net benefit for the 10-year cardiovascular-death score.

Risk decile	Mean predicted risk	Observed proportion	n
1	0.065	0.011	90
2	0.065	0.011	90
3	0.066	0.000	90
4	0.066	0.022	90
5	0.067	0.033	90
6	0.069	0.044	90
7	0.072	0.033	90
8	0.080	0.167	90
9	0.104	0.267	90
10	0.400	0.467	90

Threshold probability	Net benefit: model	Net benefit: treat-all	Net benefit: treat-none
0.05	0.058	0.058	0.000
0.10	0.053	0.006	0.000
0.15	0.037	−0.052	0.000
0.20	0.028	−0.118	0.000
0.30	0.021	−0.278	0.000
0.40	0.016	−0.491	0.000
0.50	0.012	−0.789	0.000

Table S10. Ten globally most influential features by mean absolute SHAP value in the cause-specific gradient-boosted ensembles (complete-case $n = 2225$).

Rank	CVD death feature	Mean |SHAP|	Cancer death feature	Mean |SHAP|
1	Age	1.159	Age	0.511
2	Comorbidity index	0.318	Female sex	0.235
3	Income-to-poverty ratio	0.261	C-reactive protein	0.191
4	Race/ethnicity	0.235	Smoking status	0.190
5	Neutrophil-to-lymphocyte ratio	0.182	Neutrophil-to-lymphocyte ratio	0.171
6	Systolic BP	0.160	Healthcare visits, past 12 mo	0.152
7	Serum albumin	0.155	Number of cancer sites	0.152
8	Female sex	0.153	BMI	0.137
9	Diastolic BP	0.143	HDL cholesterol	0.133
10	Self-rated general health	0.139	Time since diagnosis	0.114

Table S11. Stratum sample sizes for cancer site and time since diagnosis: full stratum n against the smaller subset eligible for SHAP decomposition (minimum 50 covariate-complete cases).

Stratum	Full n	SHAP-eligible n	SHAP computed?
Prostate	725	442	Yes
Breast	714	388	Yes
Gynecologic	631	403	Yes
Other/rare	407	239	Yes
Colorectal	344	197	Yes
Skin (melanoma)	278	173	Yes
Genitourinary (other)	226	133	Yes
Hematologic	168	100	Yes
Respiratory (other)	132	69	Yes
Digestive (other)	95	36	No (< 50)
Skin (non-melanoma/unknown)	72	45	No (< 50)
Time since diagnosis < 2 y	547	274	Yes
Time since diagnosis 2 to < 5 y	690	399	Yes
Time since diagnosis 5 to < 10 y	864	539	Yes
Time since diagnosis 10 y or more	1672	1013	Yes

Table S11 note. Site is the earliest reported diagnosis, so the non-melanoma or unknown-type skin stratum holds survivors who also reported another malignancy; survivors whose only reported cancer was of that kind are not in the cohort.

Table S12. Complete-case against multiple-imputation (MICE, ten imputations, Rubin's rules) cause-specific Cox log-hazard ratios for cardiovascular death and cancer death, 11-covariate sensitivity set. CC = complete-case (n = 2554); MI = multiply imputed (n = 3825).

Covariate	CVD CC β (SE)	CVD MI β (SE)	Cancer CC β (SE)	Cancer MI β (SE)
Age	0.132 (0.012)	0.113 (0.009)	0.038 (0.006)	0.038 (0.005)
BMI	0.007 (0.014)	0.005 (0.012)	−0.022 (0.011)	−0.034 (0.009)
C-reactive protein	0.019 (0.008)	0.016 (0.007)	0.025 (0.005)	0.028 (0.005)
Diabetes	0.606 (0.213)	0.460 (0.169)	0.243 (0.178)	0.185 (0.144)
HbA1c	−0.062 (0.105)	−0.010 (0.081)	−0.021 (0.081)	−0.036 (0.069)
Hemoglobin	−0.117 (0.055)	−0.134 (0.047)	−0.142 (0.042)	−0.113 (0.037)
Hypertension	0.167 (0.162)	0.236 (0.135)	0.115 (0.125)	0.044 (0.099)
Number of cancer sites	−0.010 (0.186)	0.019 (0.153)	0.662 (0.115)	0.720 (0.090)
Systolic BP	0.004 (0.003)	0.001 (0.003)	0.001 (0.003)	−0.001 (0.002)
Female sex	−0.334 (0.155)	−0.396 (0.128)	−0.629 (0.129)	−0.537 (0.102)
Time since diagnosis	−0.002 (0.006)	−0.003 (0.005)	−0.009 (0.006)	−0.015 (0.005)

Table S13. Headline covariates with and without C-reactive protein. The model without it is fit on all ten cycles; the primary model excludes the two cycles in which C-reactive protein was not measured.

Covariate	CVD, primary	CVD, without CRP	Cancer, primary	Cancer, without CRP
Age (per year)	1.08 (1.06–1.10)	1.08 (1.06–1.10)	1.03 (1.01–1.05)	1.04 (1.02–1.05)
Female sex	0.74 (0.50–1.08)	0.66 (0.46–0.94)	0.50 (0.34–0.74)	0.52 (0.36–0.76)
Time since diagnosis (per year)	1.00 (0.99–1.01)	1.00 (0.99–1.01)	0.99 (0.98–1.00)	0.98 (0.97–1.00)
Hypertension	0.81 (0.51–1.28)	0.83 (0.54–1.25)	1.17 (0.81–1.70)	1.20 (0.88–1.64)
Diabetes	0.89 (0.52–1.54)	0.96 (0.57–1.61)	1.06 (0.63–1.78)	1.04 (0.64–1.68)
BMI	0.99 (0.95–1.03)	1.00 (0.97–1.04)	1.00 (0.96–1.03)	0.99 (0.96–1.02)
Number of cancer sites	0.92 (0.59–1.45)	0.87 (0.56–1.34)	2.18 (1.54–3.08)	2.00 (1.41–2.82)
Comorbidity index	1.40 (1.24–1.59)	1.41 (1.26–1.58)	0.89 (0.80–1.00)	0.91 (0.81–1.02)
Smoking: never (ref. current)	0.74 (0.44–1.26)	0.72 (0.45–1.14)	0.48 (0.32–0.72)	0.58 (0.40–0.84)
