# Supplementary material for: Negative-Control Calibration and E-Value Bounding of Cancer-Survivor Mortality Contrasts: A Design-Aware Competing-Risks Analysis
Source: Cancers (Basel). 2026 Nov 4;18(21):3544. doi: 10.3390/cancers18213544 (PMC13762343; doi:10.3390/cancers18213544)
Supplement: Supplementary file 1 [file cancers-18-03544-s001.zip › Supplementary File S3.pdf]

Supplement S3. Extended Methods

This file carries the implementation detail of the analysis whose purpose and design are stated in the manuscript's Materials and Methods. Section numbers refer to the manuscript. Nothing here changes a reported estimate; it records how each estimate was computed.

2.5. Covariate harmonization across cycles

The source items and codings are as follows. Race/ethnicity is RIDRETH1 and educational attainment DMDEDUC2, each with five categories entered as indicator variables. Blood pressure is the mean of up to four MEC readings (BPXSY1–4 and BPXDI1–4). C-reactive protein is harmonized across the conventional and high-sensitivity assay eras. Smoking status is never, former, or current, derived from the lifetime and current-use items. Alcohol use is at least 12 drinks in any one year, and in 2017–2018 any drink. Physical activity is harmonized across the 2007 questionnaire redesign. All continuous laboratory and anthropometric variables were Winsorized at the 1st and 99th percentiles.

Blood pressure was measured with the same auscultatory mercury-sphygmomanometer protocol throughout 1999-2018. NHANES moved to an oscillometric protocol only in 2021, after this study's window, so no era stratification was needed.

Hypertension, diabetes, and the comorbidity index count a condition only when the participant answered "yes"; every other answer counts as not reported. In the analytic cohort this affected 119 "borderline" diabetes answers and a small number of "don't know" answers: 8 for hypertension, 1 for diabetes, and 10 to 26 for each of the five cardiovascular items.

Healthcare visits were asked with six answer categories in 1999-2012 (HUQ050) and nine in 2013-2018 (HUQ051), so the later categories were collapsed onto the earlier ones (0, 1, 2-3, 4-9, 10-12 and 13 or more visits) and the result was treated as an ordinal score.

2.6. Cardiotoxicity groups and the negative-control models

Survivors were assigned to an expected-cardiotoxicity group by their reported primary site. The **high** group is breast, where anthracyclines with or without trastuzumab are standard for most non-early-stage disease; hematologic/blood, leukemia, or lymphoma, where anthracycline-containing regimens such as CHOP or ABVD are first-line for most lymphomas and many leukemias; and prostate, included cautiously, since androgen-deprivation therapy is common but, unlike the other two members of the group, not universal, as localized low-risk prostate cancer is often managed with surveillance or surgery alone. The **low** group is non-melanoma or unknown-type skin, melanoma, thyroid, and cervix, all of which receive surgical or non-cardiotoxic systemic management for most early-stage disease. The **moderate/unclassified** group is colorectal, other genitourinary, other digestive, other respiratory, other gynecologic, and other/rare.

The full adjustment set of the negative-control models is age, sex, race/ethnicity, education, income-to-poverty ratio, body-mass index, smoking status, alcohol use, physical activity, hypertension, diabetes, the comorbidity index, healthcare visits, and self-rated general health. The calibrated contrast is the difference between the survivor and skin-cancer-only log hazard ratios, recomputed inside each jackknife replicate, so that the difference and its interval come from the same design-based resampling as the two contrasts themselves.

2.7. Fine–Gray implementation

No accessible Python package implements a Fine–Gray subdistribution-hazard fitter directly (checked: *lifelines* 0.30.3 has no `crr`/subdistribution fitter, and *scikit-survival* was not installed in this environment). We therefore used the data-transform route that R's `survival::finegray()` followed by `coxph()` uses, mathematically equivalent to Fine and Gray's original construction [10]: for each subject who fails from the *competing* event, that subject is kept in the risk set past their own event time, down-weighted by the inverse probability that they would still have been under observation at each later time, estimated from the reverse Kaplan–Meier distribution of censoring times [11]. The resulting long-format risk table was fit with a time-varying Cox model, weighted by the product of the Fine–Gray inverse-probability-of-censoring weight and the NHANES survey weight.

Every Fine–Gray fit carried a ridge (L2) penalty of 0.5 on the internally standardized coefficients (the *lifelines* `penalizer` argument), which stabilizes the weighted time-varying fit. A ridge penalty shrinks estimates toward the null. Refitting the primary model with a near-zero penalty of 0.01 changed the headline log hazard ratios by at most 0.08, so the shrinkage is small relative to the reported intervals. The negative-control Cox model (Section 2.6) used a penalty of 0.1.

2.9. Relative survival implementation

Expected mortality was estimated with a Cox model on the attained-age scale, with delayed entry at the age at examination, fit for all-cause death in the 47,482 examined adults with no reported cancer. Its Breslow baseline hazard was accumulated by attained age. Each survivor's expected survival is that baseline hazard accumulated over the attained ages they pass through, scaled by their own covariates. The demographic specification holds sex, race/ethnicity,

education, income-to-poverty ratio, smoking status, and survey period; the second adds body-mass index, hypertension, and diabetes.

Expected mortality is treated as known, as life-table rates are in a conventional relative-survival analysis, so the jackknife intervals carry sampling variability in the survivor cohort and not uncertainty in the expected-mortality model.

2.10. Gradient-boosted ensemble and SHAP

As a second, model-agnostic view of the same question, a cause-specific gradient-boosted ensemble (XGBoost [12], `survival:cox` objective, 200 trees, maximum depth 3, learning rate 0.05, subsample and column-subsample fraction 0.8) was fit separately for cardiovascular and cancer death on the 25-covariate panel, under 5-fold cross-validation with shuffled folds and a fixed seed. This is explicitly a **cause-specific hazard** model (the competing cause is treated as censored at its own event time), a different estimand from the Fine–Gray **subdistribution** hazard of Section 2.7. Pairing the two is a standard, explicitly labeled triangulation strategy in the competing-risks literature, not a silent substitution of one for the other. Discrimination was summarized by a survey-weighted Harrell's C-index on the out-of-fold risk scores. Global and stratified SHapley Additive exPlanations (SHAP) [13] were computed with `shap.TreeExplainer`, the tree-specific exact algorithm [14], on the model fit to the full covariate-complete panel, with rank stability assessed as the Spearman correlation between the full-sample SHAP ranking and each of 30 bootstrap-resampled rankings. Stratified SHAP was run separately by cancer site and by time-since-diagnosis category, with every stratum's cell size reported honestly and separately from the smaller subset that meets the pre-specified minimum-n threshold (50) for a SHAP decomposition to be reported at all (Section 3.9). Reporting stratum cell sizes alongside every SHAP decomposition guards against attributing a stratified attribution to a sample larger than the true complete-case count.

2.11. Calibration, decision curves, and simple baselines

For the cardiovascular-death gradient-boosted score, a fixed 10-year horizon was used to construct a pseudo-binary outcome: subjects with a cardiovascular death by 10 years were coded as events, subjects followed event-free (from any cause) to at least 10 years were coded as non-events, and subjects censored or removed by a competing event before 10 years were excluded. Their true 10-year status is not knowable without inverse-probability-of-censoring-weighted or pseudo-observation machinery not built here, so they were dropped from this specific calibration/decision-curve step (a documented simplification, not silently assumed away). The raw risk score was Platt-recalibrated, binned into deciles for a reliability table, and summarized with the Brier score (1000-replicate bootstrap 95% CI) and a Vickers–Elkin decision-curve analysis of net benefit against a treat-all and a treat-none strategy across threshold probabilities 0.01–0.50.

The distinction matters here for a specific reason. The full model is estimable only on covariate-complete subjects, while the reduced models are estimable on the whole cohort, so their marginal C-indices are computed on different samples and must not be differenced directly. Every paired difference is therefore evaluated on the intersection of the three models' estimable subjects, under the same delete-one-primary-sampling-unit replicate weights applied to both risk scores at once. That makes the difference a paired quantity rather than a contrast between two independently computed intervals. Marginal C-indices restricted to the same intersection are also reported, so that the three models can be read against each other on a common sample.

Two quantities are reported. The first is each model's survey-weighted C-index with a design-aware jackknife interval, computed as in Section 2.12. The second is the paired difference between the full panel and each reduced baseline, and it is the inferential comparison of the two.

2.12. Complex-survey design

NHANES is a complex, multistage probability sample, and its design was applied to every estimator reported in this article, including the machine-learning discrimination and calibration metrics and not only the descriptive statistics of Tables 1 and 2, using the masked primary-sampling-unit identifier SDMVPSU, the masked stratum identifier SDMVSTRA, and the two-year MEC examination weight WTMEC2YR, divided by ten to reflect the pooling of ten two-year cycles, per NCHS pooling guidance. Descriptive statistics used weighted proportions and quantiles. Every regression coefficient, hazard ratio, and C-index reported with a "design-aware" interval used a stratified delete-one-primary-sampling-unit jackknife: every masked PSU is dropped exactly once, with the remaining PSUs in its stratum up-weighted by $n\text{h}/(n\text{h}-1)$, and the stratified jackknife variance estimator gives a t-distribution 95% confidence interval with degrees of freedom equal to the number of jackknife units minus the number of strata. Because the Fine–Gray inverse-probability-of-censoring weight is itself a function of the survey design, design-aware Fine–Gray intervals were obtained by refitting the entire transform-plus-regression pipeline once per jackknife replicate, not by reweighting a fixed point estimate. Percentiles of the raw jackknife replicates were not used for any interval, since replicates differ from the full-sample estimate by an order smaller than the true sampling variability and their spread alone understates the interval by roughly an order of magnitude. No fasting-subsample weight was required, since no fasting-glucose variable entered the final covariate panel (Section 2.13).

2.13. Model diagnostics

Every candidate covariate was screened for target leakage, meaning algebraic or definitional downstream-ness relative to the outcome, before any model was fit (Table S1). Age at diagnosis was excluded because age equals age at diagnosis plus time since diagnosis exactly, so retaining all three would be perfectly collinear. Follow-up time, the mortality-status flag, the raw cause-of-death code, and the two cause-specific death flags were excluded as components or direct restatements of the outcome. The fine-grained site variables (`site_group`, `site_name`) were reserved for stratification rather than entered as model covariates, to avoid a ten-plus-level categorical block dominating a model fit on fewer than 4000 people. Variance-inflation factors were computed on the standardized design matrix of the covariate-complete sample ($n = 2225$). The maximum observed VIF was 3.32 (the non-Hispanic white indicator of race/ethnicity), under the conventional flag threshold of 5, so multicollinearity was not a material concern for any reported coefficient. Multiple-comparison correction (Holm–Bonferroni) was applied as described in Section 2.7.

2.14. Missing data

NHANES did not measure C-reactive protein in 2011–2012 or 2013–2014. Because every model is complete-case and C-reactive protein is in the fully adjusted panel, no participant from those two cycles enters the fully adjusted models: 382 survivors from 2011–2012 and 397 from 2013–2014, so the analytic sample of those models covers eight of the ten pooled cycles rather than ten. Section 3.11 reports a sensitivity analysis that drops C-reactive protein, in which all ten cycles contribute. Listwise deletion on the fully adjusted 25-covariate Fine–Gray model set retained a covariate-complete analytic sample of $n = 2225$ of 3826 (Section 3.3). On a compact 11-covariate set used specifically for this sensitivity check (age, sex, body-mass index, systolic blood pressure, glycated hemoglobin, C-reactive protein, hemoglobin, hypertension, diabetes, time since diagnosis, and number of cancer sites), listwise deletion dropped 33.2% of the survivor cohort (complete-case $n = 2555$ of 3826). That exceeds the 10% threshold we set for a multiple-imputation sensitivity analysis. Multiple imputation by chained equations (MICE, ten imputations, ten burn-in iterations, fixed seed) [15] with Rubin's-rules pooling was run for a compact logistic model of each cause-specific death indicator on this 11-covariate set, unweighted by survey design (the available MICE/Rubin implementation does not carry survey weights through pooling in a design-consistent way), and is reported as a sensitivity check on the *direction and magnitude* of listwise-deletion bias, not as a replacement for the design-weighted primary Fine–Gray analysis (Section 3.11).

2.15. Decomposition of the comorbidity index

The five-component count was reconstructed from the analysis panel as the index minus the hypertension and diabetes indicators, which are derived from the same two items (BPQ020 and DIQ010) that supply the index's two non-cardiovascular components. The reconstruction is therefore exact rather than approximate, and it was verified on load to lie between 0 and 5 with no negative values.
